# Deazaflavin reductive photocatalysis involves excited semiquinone radicals

**DOI:** 10.1038/s41467-020-16909-y

**Published:** 2020-06-23

**Authors:** Andreas Graml, Tomáš Neveselý, Roger Jan Kutta, Radek Cibulka, Burkhard König

**Affiliations:** 1 Institute of Organic Chemistry, University of Regensburg, 93040 Regensburg, Germany; 2 Department of Organic Chemistry, University of Chemistry and Technology, Prague, 16628 Prague, Czech Republic; 3 Institute of Physical and Theoretical Chemistry, University of Regensburg, 93040 Regensburg, Germany

**Keywords:** Enzymes, Photocatalysis, Reaction mechanisms

## Abstract

Flavin-mediated photocatalytic oxidations are established in synthetic chemistry. In contrast, their use in reductive chemistry is rare. Deazaflavins with a much lower reduction potential are even better suited for reductive chemistry rendering also deazaflavin semiquinones as strong reductants. However, no direct evidence exists for the involvement of these radical species in reductive processes. Here, we synthesise deazaflavins with different substituents at C5 and demonstrate their photocatalytic activity in the dehalogenation of *p*-halogenanisoles with best performance under basic conditions. Mechanistic investigations reveal a consecutive photo-induced electron transfer via the semiquinone form of the deazaflavin as part of a triplet-correlated radical pair after electron transfer from a sacrificial electron donor to the triplet state. A second electron transfer from the excited semiquinone to *p*-halogenanisoles triggers the final product formation. This study provides first evidence that the reductive power of excited deazaflavin semiquinones can be used in photocatalytic reductive chemistry.

## Abstract (web summary)

Flavins and deazaflavins are well suited for photoredox processes but their application in photoreductions is challenging. Here, the authors provide direct evidence of the high reductive power of excited deazaflavin semiquinones and their application in catalytic photodehalogenations.

## Introduction

**Fig. 1:**
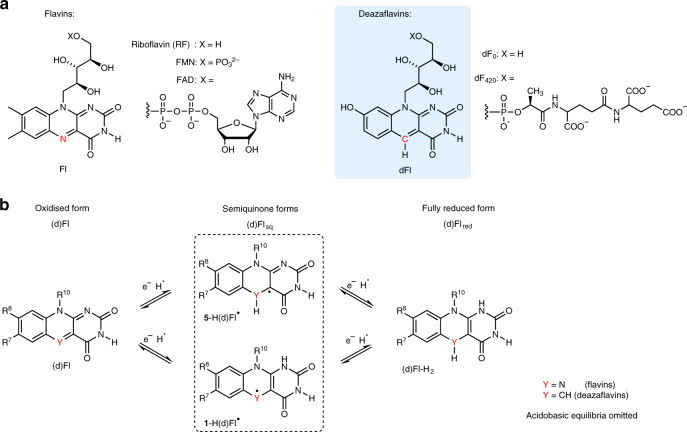
Flavins, deazaflavins, and their redox forms. **a** Structure of flavin and deazaflavin cofactors. **b** Redox states of flavins and deazaflavins. In contrast to flavins, semiquinone deazaflavins are untypical intermediates in redox chemistry.

Flavins, in the form of flavin mononucleotide (FMN) or flavin adenine dinucleotide (FAD), act as cofactors in various types of enzymes^1,2^. Some organisms also use deazaflavins (dFls; dF_0_ or dF_420_, see Fig. 1a) where the *N*-5 atom of the isoalloxazine ring is replaced by a carbon atom^3,4^. Despite their structural similarity, their roles in biological systems differ substantially^3,5^. Flavins (Fls) form semiquinones (Fl_sq_s) (Fig. 1b), which are transiently stable in solution^6^ and more stable in protein environments^7,8^. Fl_sq_s are typically involved in one-electron redox processes^1,9^. Fully reduced flavin (Fl_red_) participates in two-electron reactions or, similarly as Fl_sq_, interacts readily with molecular oxygen (O_2_), forming reactive oxygen species (ROS) or flavin hydroperoxide (FlOOH)^10–13^. FlOOH is the key agent in oxygenations mediated by flavin-dependent monooxygenases^14,15^. In contrast to Fls, dFls serve exclusively as two-electron carriers, thus behaving rather like nicotinamide adenine dinucleotide (phosphate) (NAD(P)H)^3,5^. Fully reduced deazaflavins (dFl_red_s) are considerably stable against oxidation by oxygen, thus avoiding formation of ROS^10^. This is one reason why dFls have been tested as native coenzyme (FAD or FMN) substitutes^16–18^.

Both Fls and dFls absorb visible light enabling their involvement in light-dependent processes^19–22^. Fl cofactors in their fully reduced form participate in photo-induced electron transfer (PET) reactions in DNA photolyases repairing pyrimidine dimers formed upon exposure to ultraviolet (UV) light^23,24^. Fls in their oxidised form are responsible for bacterial bioluminescence^25^ or perform various functions in photoreceptors^7,26–28^. dFl dF_0_ in its oxidised form serves as a light-harvesting antenna in photolyases^29^. Inspired by nature, artificial Fl derivatives as well as flavoenzymes have been applied in photoredox catalysis and photobiocatalysis, mainly in oxidative transformations or in energy transfer processes^6,20,30–44^. Photoenzymatic radical polymerisation employing highly reducing excited FADH*^−^ known from photolyases is rare and a very recent example of a photoreductive process with Fl^45^. In the dFl series, dFl_red_ has been found to be generated from dFl by PET from ethylenediaminetetraacetic acid used as sacrificial reductant^46^. This procedure has been utilised in the NAD(P)H-independent regeneration of FADH_2_ from FAD^16,47^.

dFl belong to the redox cofactors with the lowest redox potential; the first reduction potential of dFl [*E*(dFl/dFl_sq_)] is even more negative than that of Fls by 0.5 V^3^. Therefore, dFls are better suited for reductive chemistry than Fls. The very negative first reduction potential renders the dFl semiquinone (dFl_sq_) as a strong reducing agent. Despite this fact, there is no precedence of reductive processes involving this radical species. This might result from the very low stability of the dFl_sq_ free in the solution^3,5,48^.

Here we demonstrate that the reducing power of excited dFl radicals can be used in the presence of two substrates: (i) a sacrificial electron donor, e.g. diisopropyl(ethyl)amine (DIPEA), allowing the photochemical formation of the radical dFl_sq_ and (ii) a substrate for reduction, *p*-halogenanisole, undergoing dehalogenation via the excited radical dFl_sq_*.

## Results

### Design and synthesis

**Fig. 2:**
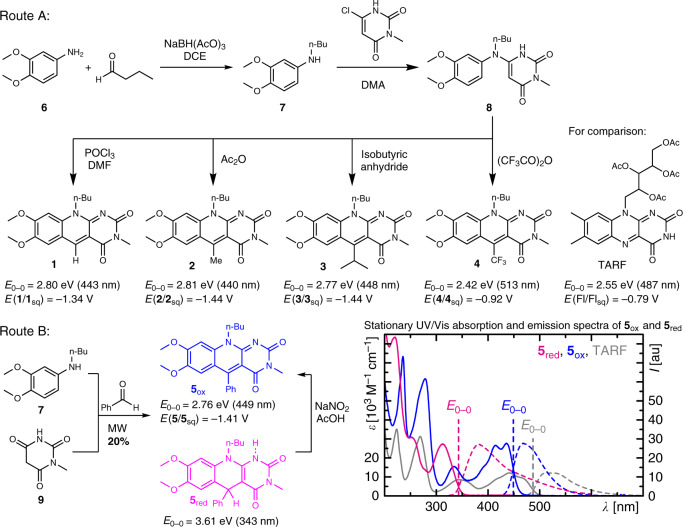
Synthesis and spectral and electrochemical properties. Electronic transition energies, *E*_0-0_, and first reduction potentials vs. saturated calomel electrode (SCE) are given for all deazaflavins. Full spectra are shown for **5**_ox_ and **5**_red_. Data for tetraacetyl riboflavin (TARF) are given for comparison. Further details on synthesis and characterisation are given in Supplementary Notes 1 and 2, respectively.

A series of 5-dFls **1**–**5** was synthesised with different substituents in position 5 (H-, Me-, *i*Pr-, CF_3_-, and Ph-) via two different routes depending on the substituent (Fig. 2 and Supplementary Notes 1 and 2). Derivatives **1**–**4** were obtained following route A starting from 3,4-dimethoxyaniline (**6**). Reductive amination yielded compound **7**, which was subsequently coupled with 6-chloro-3-methylpyrimidine-2,4(1*H*,3*H*)-dione to give access to the key intermediate **8**. Depending on the reaction conditions for the following step, the corresponding products **1**–**4** were obtained. As this synthetic method did not allow the synthesis of the phenyl derivative **5**, route B was developed. In this three-component microwave-assisted procedure, adapted from Tu et al.^49^, compound **5** was obtained from a reaction of benzaldehyde with *N*-butyl-3,4-dimethoxyaniline (**7**) and *N*-methylbarbituric acid (**9**). Following this procedure, the phenyl derivative could be isolated in both the oxidised (**5**_ox_) and fully reduced form (**5**_red_), which reflects the low sensitivity of the dFl_red_ against O_2_^3,50^. This observation is further supported by incubation of **5**_red_ in darkness for 19 h in non-degassed acetonitrile (ACN) where no considerable formation of **5**_ox_ is observed via absorption spectroscopy (Supplementary Note 3). However, light excitation of **5**_red_ allows to overcome the activation barrier for re-oxidation by O_2_ resulting in almost stoichiometric conversion to **5**_ox_ (Supplementary Note 3).

### Photophysical and electrochemical characterisation

Compounds **1**–**3** have similar UV/visible (Vis) absorption and emission spectra in ACN giving similar electronic transition energies of these dFls with the usual blue-shift of around 40 nm compared to isoalloxazines (Fig. 2 and Supplementary Fig. 1)^51^. In contrast, derivative **4** with the strong electron withdrawing group -CF_3_ shows a red-shifted electronic transition by 26 nm in ACN compared to isoalloxazines. The compounds **1**–**4** are all highly emitting with fluorescence quantum yields ranging from 67% to 85% in ACN. Their emission lifetimes in ACN range between 6.8 and 11.1 ns (Supplementary Note 2). In agreement with literature reports^4^, cyclo-voltammetry (CV) shows more negative redox potential of the first reduction step of the dFls compared to tetraacetyl riboflavin (TARF) (*E*(TARF/TARF_sq_) = −0.79 V, ref. ^39^) by 0.13–0.52 V (Supplementary Note 4).

The phenyl derivative **5** shows deviating characteristics compared to the other dFls. While the electronic transition energies are also blue-shifted compared to isoalloxazines and the redox potential of the first reduction step is also more negative compared to TARF, its emission properties are significantly different. The fluorescence quantum yield reaches only 16% and its excited singlet state lifetime is considerably reduced being only 1.7 ns (Supplementary Note 2). Furthermore, compound **5** is the only derivative that forms a thermally stable fully reduced form **5**_red_, which is only re-oxidised by light as discussed above (Supplementary Note 3).

### Photodehalogenation

Owing to the strong reduction potential of dFl derivatives compared to their Fl analogues, we investigated their use in the reductive photoredox catalysis for the dehalogenation of aryl halides, which is considered as a benchmark reaction because of their very negative redox potentials. For instance, with a one-electron reduction potential of −2.75 V^52^, *p*-bromoanisole (*p*-BA) is beyond the limits of most organic photoredox catalysts^53,54^, or it requires combination of electrocatalysis and photocatalysis for dehalogenation^55,56^.

We achieved an almost quantitative yield of *p*-BA dehalogenation using the phenyl derivative **5** (6 mM, 8 mol% relative to substrate), when irradiating at 385 nm in the presence of a sacrificial reducing agent, here we used DIPEA, and caesium carbonate (Cs_2_CO_3_) (Table 1). The anisole yield is independent on the oxidation state of the photocatalyst, i.e. **5**_ox_ or **5**_red_, since the dFl_red_ **5**_red_ is re-oxidised by O_2_ and light as described above (Supplementary Note 3). Noteworthy, **5**_ox_ also showed photo-dehalogenation of *p*-chloroanisole, whose redox potential is even more negative (*E*_red_ = −2.88 V, ref. ^52^).

**Table 1:** Photocatalytic dehalogenation of *p*-halogenanisole by deazaflavin **5**_ox_ and **5**_red_.


Substrate	*E*_red_ vs. SCE [V]	Photocatalyst	Product yield [%]^a,b^
X = Br	−2.75	**5**_ox_	79
X = Br	−2.75	**5**_red_	80
X = Cl	−2.88	**5**_red_	80

^a^Reaction conditions: *p*-XA (75 mM (0.15 mmol)), **5**_ox_ or **5**_red_ (6 mM (8 mol%)), DIPEA (150 mM (0.3 mmol)), Cs_2_CO_3_ (75 mM (0.15 mmol)), ACN (2 mL), *λ*_exc_ = 385 nm, 25 °C, nitrogen atmosphere, 18 h.

^b^Yields determined via calibrated GC analysis with 4-methylanisole as internal standard.

We next investigated the influence of the solvent and the excitation wavelength on the total product yield (Supplementary Note 5). Interestingly, when dimethylformamide was used as solvent under otherwise identical conditions the product yield decreased by almost 50%. With respect to the excitation wavelength, the best yield, ca. 80%, was obtained for wavelengths ≤400 nm. When using light of 455 nm, only 50% yield could be obtained. As a control, in the absence of photocatalyst, DIPEA, or irradiation, no reaction occurs. Recording the product build-up in case of **5**_ox_ showed that 60% conversion was observed already after 6 h.

Furthermore, we investigated the effect of basicity on the dehalogenation efficiency of *p*-BA (Table 2). Without any base, only yields between 10% and 30% are observed for all photocatalysts. In the presence of Cs_2_CO_3_, under otherwise identical conditions, the yield was increased to >60% for the dFls **3** and **5**, while the reactivity of dFls **1** and **4** remained unchanged or increased to only 38% for the methyl derivative **2**. For the dFls **1** and **5**, we then tested bases with increasing basicity, i.e. NaHCO_3_, Cs_2_CO_3_, and KO^t^Bu. While the activity of **5** was significantly enhanced by Cs_2_CO_3_ or KO^t^Bu, the reactivity of **1** only increased in case of KO^t^Bu. These findings already indicate an important acidobasic equilibrium of a potential intermediate species of the photocatalyst that affects the overall performance. Thus a deprotonated form of one key intermediate seems to be beneficial (A more detailed discussion can be found in Supplementary Note 6).

**Table 2:** Photocatalytic dehalogenation of *p*-bromoanisole by deazaflavin in dependence on the basicity.


Base	Product yield [%]^a,b^ using photocatalyst					
	1	2	3	4	5_ox_	5_red_
None	22	12	15	14	24	27
NaHCO_3_	20	–	–	–	22	–
Cs_2_CO_3_	19	38	67	15	60	64
KO^t^Bu	50	–	–	–	59	–

^a^Reaction conditions: *p*-BA (75 mM (0.15 mmol)), dFl (3 mM (4 mol%)), DIPEA (150 mM (0.3 mmol)), base (75 mM (0.15 mmol)), ACN (2 mL), *λ*_exc_ = 385 nm, 25 °C, nitrogen atmosphere, 6 h.

^b^Yields determined via calibrated GC analysis with 4-methylanisole as internal standard.

### Excited singlet state reaction results in a loss channel

**Fig. 3:**
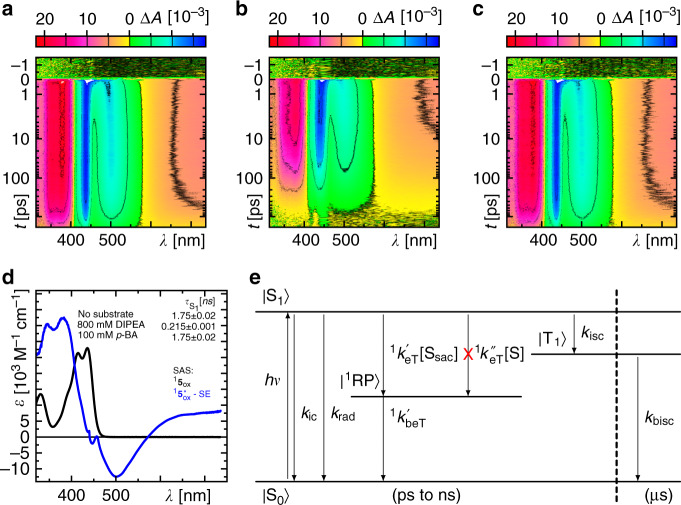
Excited singlet state dynamics of 5_ox_. **a**–**c** False colour representation of the time-resolved absorption spectra of **5**_ox_ (360 μM) in non-degassed ACN in the absence (**a**) or presence of either 800 mM DIPEA (**b**) or 100 mM *p*-BA (**c**) exciting at *λ*_exc_ = 450 nm. **d** Species-associated spectra (SAS) that contribute to the time-resolved absorption signals in **a**–**c** with excited singlet state lifetimes as indicated. **e** Model used to describe the time-resolved absorption data that results in physically reasonable SAS. On this time window, *k*_isc_ is negligible, and since no further species spectrum is detectable, the assumption ^1^*k*'_beT_ ≫ ^1^*k*′_eT_ [S_sac_] is justified. SE stimulated emission, rad radiative, ic internal conversion, (b)eT (back) electron transfer, (b)isc (back) intersystem crossing, S_0_ singlet ground state, S_1_ excited singlet state, T_1_ triplet state, ^1^RP singlet born radical pair, S_sac_ sacrificial electron donor, S substrate.

The reactivity of the excited states of the compounds **1** and **5**_ox_ with the sacrificial substrate DIPEA was investigated by time-resolved absorption and emission spectroscopy in the absence of any additional base, since the main focus was on the understanding of the underlying photocatalytic reaction mechanism (Further studies on the role of specific deprotonation states is currently ongoing in our laboratories and will be presented elsewhere.). In both cases, the excited state quenching was observed without the formation of any additional transient species (Fig. 3 and Supplementary Note 7). This indicates the formation of a singlet born radical pair that recombines faster than it is formed owing to spin-allowed radical pair recombination (Fig. 3e and Supplementary Note 7). Such a fast recombination reaction was already observed in the Fl photo-oxidation of aromatic alcohols and represents a pure loss channel for photocatalytic applications^6^. In case of the reaction between DIPEA and the excited singlet of the dFl, the bi-molecular reactions rates for **1** and **5**_ox_ under low substrate concentrations (<200 mM DIPEA) are (1.05 ± 0.01) × 10^10^ M^−1^ s^−1^ and (9.0 ± 0.1) × 10^9^ M^−1^ s^−1^, respectively (see Stern–Volmer analysis in Supplementary Note 7). These values are smaller by 56% and 47%, respectively, compared to the theoretical diffusion limited bi-molecular reaction limit (see Supplementary Note 7 for a detailed discussion), which might indicate a geometrical preference for reactive encounters allowing for necessary interactions, and thus enabling a high probability for the reaction to proceed.

In the presence of only the actual substrate *p*-BA (with a concentration of 100 mM, which is sufficiently high for diffusion controlled encounters with the excited singlet state of the photocatalyst), the transient absorption (TA) data of **1** and **5**_ox_ resemble those of the photocatalysts alone demonstrating no reaction between *p*-BA and the corresponding excited states (Fig. 3c and Supplementary Note 7). In addition, in case of the stable fully reduced form, **5**_red_, also no reaction between the excited singlet state and the substrate *p*-BA is observed (Supplementary Note 7).

### Triplet state reaction forms transiently stable semiquinone

**Fig. 4:**
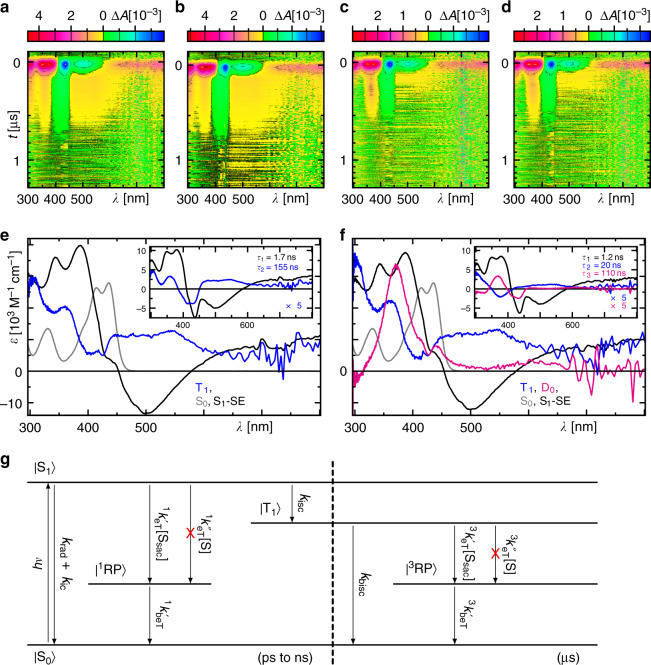
Excited singlet and triplet state dynamics of 5_ox_. **a**–**d** False colour representation of the time-resolved absorption spectra of **5**_ox_ (60 μM) in non-degassed ACN in the absence (**a**) or presence of either 50 mM *p*-BA (**b**), 50 mM DIPEA (**c**), or both 50 mM *p*-BA and 50 mM DIPEA (**d**) exciting at *λ*_exc_ = 430 nm. **e** Species-associated spectra (SAS) that contribute to the time-resolved absorption signals in **a**–**b**, which were generated by applying the model in **g** to the decay-associated difference spectra (DADS) shown in the inset. **f** Same as **e** but for the data in **c**, **d**. SE stimulated emission, rad radiative, ic  internal conversion, (b)eT (back) electron transfer, (b)isc (back) intersystem crossing, superscript 1/3 singlet or triplet state, RP spin correlated radical pair containing the **5**_sq_ (D doublet state), S_sac_ sacrificial electron donor, S substrate.

**Fig. 5:**
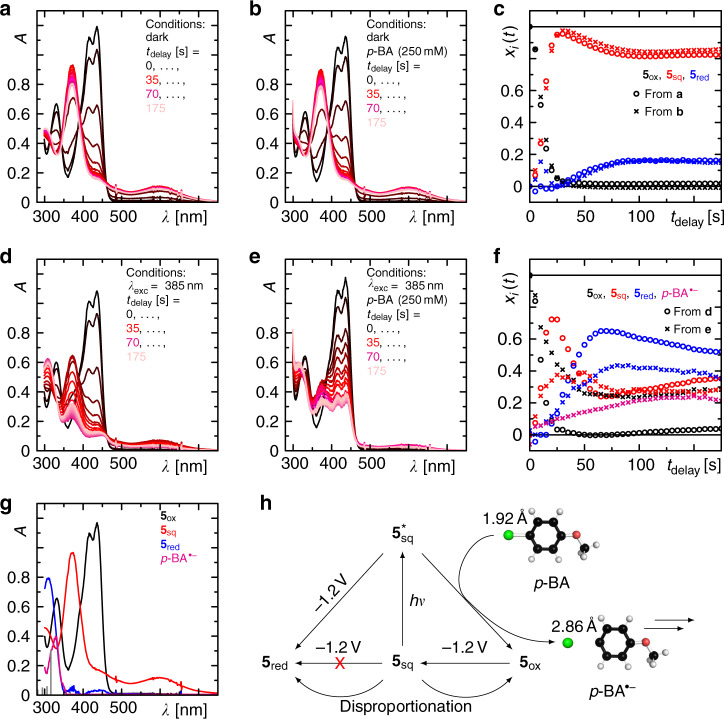
Reactivity of electrochemically formed semiquinone 5_sq_. **a**, **b**, **d**, **e** Sequences of UV/Vis absorption spectra after application of −1.2 V to the sample at conditions as indicated. **c**, **f** Concentration–time profiles from fitting the species spectra shown in **g** to the data as indicated. The grey vertical lines in **g** correspond to the quantum chemically calculated stick spectrum of *p*-BA^•‒^ as described in the text. **h** Summary of all the observed processes. The stick and ball structure of *p*-BA and *p*-BA^•‒^ are the result of quantum chemical calculations as described in the main text and Supplementary Note 11. The significantly reduced Br^−^ dissociation energy in *p*-BA^•−^ demonstrates the final bromine dissociation upon one electron reduction.

TA spectroscopy in the ns to μs range was used in order to study the triplet state formation of **5**_ox_ (Fig. 4a, e as well as further details in Supplementary Notes 2 and 8). In comparison to isoalloxazines, its yield is low being only ca. 11% (Supplementary Table 1). In the presence of the substrate *p*-BA (50 mM), no reaction is observed with either the excited singlet or triplet state of **5**_ox_ (Fig. 4b, e). In contrast, in the presence of the sacrificial electron donor DIPEA (50 mM) electron transfer (eT) from DIPEA to the triplet state occurs forming the semiquinone **5**_sq_ (Fig. 4c, f), whose spectrum agrees well with the electrochemically generated spectrum (Fig. 5g and Supplementary Note 9). The yield for eT can be determined from the corresponding decay rates to $\Phi _{{\mathrm{eT}}} = 1 - k_0k_{\mathrm{q}}^{ - 1} = 87\%$, where *k*_0_ is the total decay rate constant of the triplet in the absence and *k*_q_ in the presence of DIPEA. Considering the concentration of DIPEA (50 mM), the corresponding bi-molecular reaction rate with the triplet state is given to be (8.7 ± 0.1) × 10^8^ M^−1^ s^−1^ (*k*_eT_ = *Φ*_eT_*k*_q_ ([DIPEA])^−1^). However, in the available spectral window of our experiment, we do not observe spectral signatures of the corresponding DIPEA radical cation. The assignment as the semiquinone radical is further proven by recording its electron paramagnetic resonance spectrum under photo-stationary conditions (Supplementary Note 10). In the presence of both *p*-BA and DIPEA, again, only the triplet reaction with DIPEA is observed, but no indication for a reaction between *p*-BA and **5**_sq_ is found (Fig. 4d, f). In summary, while the excited singlet state reaction with DIPEA results in a singlet born spin correlated radical pair, ^1^[**5**_sq_, DIPEA^•+^], which recombines faster than it is formed owing to spin allowance, the triplet reaction with DIPEA involves a triplet born radical pair, ^3^[**5**_sq_, DIPEA^•+^], which is spin forbidden for recombination, thus allowing its accumulation (Fig. 4g).

### Reactivity of the excited dFl_sq_

In the next step, we investigated the reactivity of **5**_sq_ in its excited state **5**_sq_* towards the dehalogenation of *p*-BA. For this purpose, we generated the semiquinone form **5**_sq_ electrochemically without the need of the light-induced reaction with DIPEA by applying constantly −1.2 V to the sample in ACN and followed its reactivity by UV/Vis absorption over time under the following four conditions: (1) alone in the dark, (2) in the dark and the presence of *p*-BA (250 mM), (3) alone in the light (*λ*_exc_ = 385 nm), and (4) in the light (*λ*_exc_ = 385 nm) and in the presence of *p*-BA (250 mM). Alone in the dark, initially the concentration of **5**_ox_ depletes completely and **5**_sq_ is formed simultaneously (Fig. 5a). On longer delays, the concentration of **5**_sq_ reduces by ca. 18% with the simultaneous formation of **5**_red_ indicating partial disproportionation of two **5**_sq_ molecules into **5**_ox_ and **5**_red_^19^. The former does not recover considerably because the electrode-driven one electron reduction immediately forms **5**_sq_. Using the species spectra obtained from the spectro-electrochemical experiment (Fig. 5g and Supplementary Note 9), the sequence of spectra can be perfectly decomposed in order to obtain the corresponding concentration–time profiles of the contributing species (Fig. 5c). Repeating this experiment in the dark but now in the presence of *p*-BA, exactly the same behaviour is observed within the experimental error (Fig. 5b, c) demonstrating no reaction between *p*-BA and either of the dFl redox species in their ground states. This additionally confirms the results of the TA spectroscopy.

However, in the experiments with simultaneous illumination of the sample, significantly different kinetics are observed compared to the situation in the dark. In the absence of *p*-BA but presence of light, again, a rapid reduction of **5**_ox_ is observed (Fig. 5d). However, under these conditions **5**_sq_ does not accumulate to the same extent (here only to 75% in the transient maximum) as in the case without illumination. Instead, a significantly enhanced formation of **5**_red_ up to 70% in the transient maximum is observed, indicating a light-induced second eT from the electrode to the excited **5**_sq_*. Again, these data can be decomposed well with the known species spectra of the three redox states of **5** (Fig. 5f). In the final experiment with light and *p*-BA, again, different kinetics are observed compared to the three experiments described before. In this case, **5**_ox_ is only converted to ca. 75%. This is accompanied by a significantly reduced accumulation of **5**_sq_ and **5**_red_ (Fig. 5e), thus indicating a reaction between *p*-BA and **5**_sq_ in its excited state reforming **5**_ox_. Using only the known species-associated spectra (SAS) of the dFl intermediates, this time, the decomposition of the data shows significant deviations. Closer inspection of the arising absorption band at 300 nm reveals an asymmetric shape compared to the expected absorption band of **5**_red_ indicating some underlying spectral contributions of a different species than those already known from the dFls. After subtraction of a fitted linear combination of the known spectral features of dFl, an absorption spectrum with a single absorption band peaking at 330 nm remains (magenta spectrum in Fig. 5g). Considering a potential radical anion of *p*-BA as an intermediate arising after eT from excited **5**_sq_* to *p*-BA, we calculated the absorption spectrum of *p*-BA^•‒^ quantum chemically using a high level of theory, i.e. state-averaged XMCQDPT-CASSCF(12,12), considering also the solvent environment by the polarisable continuum model (PCM) for ACN (see Supplementary Note 11). As seen in Fig. 5g and Supplementary Fig. 12a, the calculated spectrum matches the measured spectrum accurately, so that we assign this spectrum to *p*-BA^•‒^. Considering the *p*-BA^•‒^ spectrum together with the already determined species spectra for all dFl intermediates now results in a good fit with deviation within the experimental error. Since the fit does not depend on the scaling of the *p-*BA^•‒^ spectrum, we scaled the spectrum arbitrarily so that the resulting concentration–time profile stays below all Fl contributions. Furthermore, the quantum chemical calculation revealed a significantly longer bond length between the bromine and the adjacent carbon atom in *p*-BA^•‒^ (2.86 Å) than in *p*-BA (1.92 Å) as well as a significantly reduced dissociation energy being close to thermal energy fluctuations (Supplementary Fig. 12b), which explains well the observed final dissociation of the bromine during the dehalogenation, which is triggered by a single electron reduction step as summarised in Fig. 5h (Further details are given in Supplementary Note 11.).

Analogous experiments were conducted with the non-substituted dFl **1**. However, in contrast to the experiments with **5**, the semi-reduced form could not be observed on the time scale of the measurements. The failure of detecting the semi-reduced form **1**_sq_ indicates that its lifetime is significantly shorter compared to that of **5**_sq_. This agrees with the observation of a significantly lower yield in the dehalogenation using **1** compared to **5**, since less **1**_sq_ may be accumulated.

### Photocatalytic reaction mechanism based on consecutive PET (conPET)

**Fig. 6:**
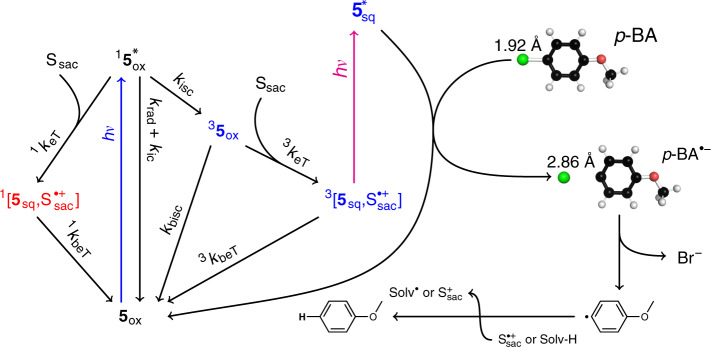
Photocatalytic reaction mechanism. A sacrificial electron donor, e.g. DIPEA, reacts either with the excited singlet or triplet state of the deazaflavin. In the excited singlet state reaction, a singlet born spin correlated radical pair, ^1^[**5**_sq_, ${\mathrm{S}}_{{\mathrm{sac}}}^ {•+}$], is formed after eT. This radical pair recombines faster than it is formed. In contrast, eT to the triplet state results in a triplet born radical pair, ^3^[**5**_sq_, ${\mathrm{S}}_{{\mathrm{sac}}}^ {•+}$], allowing the accumulation of the considerably stable **5**_sq_. **5**_sq_ can be excited by a second photon enabling a consecutive photo-induced electron transfer from **5**_sq_* to the substrate, *p*-BA, regenerating **5**_ox_ in its ground state and closing the photocatalytic cycle. Finally, anisole is formed via hydrogen abstraction from either a ${\mathrm{S}}_{{\mathrm{sac}}}^ {•+}$ or a solvent molecule.

In summary, our results can be compiled to the photocatalytic reaction mechanism depicted in Fig. 6. With respect to the redox potential neither **5**_red_ (*E*(**5**_sq_/**5**_red_) = −1.6 V) nor **5**_sq_ (*E*(**5**_ox_/**5**_sq_) = −1.41 V) can reduce *p*-BA (*E*_red_ = −2.75 V) in their ground states. The participation of the excited fully reduced form **5**_red_* is not observed most likely due to very efficient intrinsic deactivation of the excitation energy as seen in fast deactivation kinetics on a ps time scale (Supplementary Note 7 and Supplementary Fig. 8). In the presence of a sacrificial electron donor DIPEA, we observe different reactivities for either the excited singlet or triplet state of **5**_ox_. In the excited singlet state reaction, a singlet born spin correlated radical pair, ^1^[**5**_sq_, DIPEA^•+^], is formed, which recombines faster than it is formed. Accordingly, this reaction represents a pure loss channel. In contrast, eT to the triplet state results in a triplet born radical pair, ^3^[**5**_sq_, DIPEA^•+^], that is spin forbidden for recombination, thus allowing the accumulation of the considerably stable **5**_sq_. As a consequence, **5**_sq_ can be excited by a second photon (E*(**5**_ox_/**5**_sq_) = −3.3 V), which enables a conPET^57^ from **5**_sq_* to the substrate, *p*-BA, regenerating **5**_ox_ in its ground state and closing the photocatalytic cycle. The dissociation energy for the bond between the bromine and the adjacent carbon in *p*-BA^•‒^ is significantly reduced leading to thermally driven dissociation into Br^–^ and the aryl radical. Finally, anisole is formed via hydrogen abstraction from either a DIPEA radical cation or a solvent molecule^57^. Owing to the lack of the observation of any protonated **5**_sq_ form (see Supplementary Note 6), the radical anion of **5**_sq_ is the active species.

## Discussion

We have described the detailed photocatalytic mechanism of a reductive dehalogenation of *p*-halogenanisole by dFls. The key point is the conPET via the anionic semiquinone form of the dFl. In doing so, we have revealed that dFls are valuable photocatalysts for reductive chemistry. In summary, after photoexcitation of dFl followed by intersystem crossing a sacrificial electron donor, at a moderate concentration, will enable a first eT to the triplet state resulting in a considerably stable anionic semiquinone. Additional excitation of the anionic semiquinone provides sufficient energy for a second eT process from the semiquinone to compounds with highly negative redox potentials, such as *p*-halogenanisoles. Finally, downstream reactions resulting in product formation occur spontaneously.

The action spectrum of the conPET reaction comprises the absorption of two different coloured photons by the two key photocatalytic forms of dFl, where one is formed from the other having a distinct lifetime in the ns regime. Therefore, a concrete timed illumination of the photocatalytic system with short and intense pulses at the appropriate excitation wavelengths, which are temporally optimised to the lifetime of the key intermediates, should allow further improvement on the efficiency. This aspect is of general importance for all conPET-type reactions and should be addressed in future work.

To our knowledge, this study shows for the first time that the reductive power of excited dFl_sq_ can be used in photocatalytic reductive chemistry. Moreover, because of its very negative potential, this species seems to belong to the most powerful reductants^53,55,56,58–61^. Therefore, this study expands the photocatalytic toolbox for new chemical transformations and should serve as a guide for engineering photocatalysts of the next generation.

## Methods

### Materials

Starting materials and reagents were purchased from commercial suppliers (Sigma Aldrich, Alfa Aesar, Acros, Fluka, VWR, or Fluorochem) and were used without further purification. Solvents were used as p.a. grade or dried and distilled according to literature known procedures.

### Nuclear magnetic resonance (NMR) spectroscopy

NMR spectra were recorded at room temperature using a Bruker Avance 300 (300 MHz for ^1^H, 75 MHz for ^13^C), a Bruker Avance 400 (400 MHz for ^1^H, 101 MHz for ^13^C), an Agilent 400-MR DDR2 (400 MHz for ^1^H and 101 MHz for ^13^C), or a Varian Mercury Plus 300 (300 MHz for ^1^H, 75 MHz for ^13^C) with internal solvent signal as reference. All chemical shifts are reported in δ-scale as parts per million (multiplicity, coupling constant *J*, number of protons) relative to the solvent residual peaks as the internal standard (IS). NMR spectra are available in Supplementary Figs. 13–22.

### Mass spectrometry

The mass spectrometric measurements were performed at the Central Analytical Laboratory of the University of Regensburg or at the Central Analytical Laboratory of UCT, Prague on a Finnigan MAT 95, ThermoQuest Finnigan TSQ 7000, Finnigan MATSSQ 710A, Agilent Q-TOF 6540 UHD, or a LTQ Orbitrap Velos (Thermo Fisher Scientific).

### Gas chromatography (GC)

GC measurements were performed on a GC 7890 from Agilent Technologies. Data acquisition and evaluation was done with Agilent ChemStation Rev.C.01.04. A capillary column HP-5MS/30 m × 0.25 mm/0.25 μM film and helium as carrier gas (flow rate of 1 mL/min) were used. The injector temperature (split injection: 40:1 split) was 280 °C, detection temperature 300 °C (FID). The GC oven temperature programme was adjusted as follows: the initial temperature of 40 °C was kept for 3 min and was increased at a rate of 15 °C/min until the injection temperature of 280 °C was reached. After 5 min, the temperature was further increased at a rate of 25 °C/min until the final temperature of 300 °C was reached and kept for 5 min. Using 4-methylanisole as an IS, we obtained the GC calibration given in Supplementary Fig. 23 for the quantitative analysis.

### Cyclo-voltammetry

CV measurements were performed with the three-electrode potentiostat galvanostat (PGSTAT302N, Metrohm Autolab). The control of the measurement instrument, the acquisition, and processing of the CV data were performed with the software Metrohm Autolab NOVA 1.10.4. Electrochemical studies were carried out under argon atmosphere in ACN containing 0.1 M tetra-*N*-butylammonium hexafluorophosphate using ferrocene/ferrocenium (Fc/Fc^+^) as an internal reference. A glassy carbon electrode (working electrode), platinum wire counter electrode, and Ag quasi-reference electrode were employed.

### Spectro-electrochemistry

Measurements were performed in an Ottle Cell (Optically transparent thin-layer electro-chemical cell), pathlength = 0.02 cm, working electrode: Pt minigrid, counter electrode: Pt minigrid, pseudo reference electrode: Ag wire. Samples were prepared by degassing a solution of **5**_ox_ in ACN (3 mM) via bubbling of Argon for several minutes. The substrate, *p*-BA, was added via a Hamilton syringe resulting in a solution of 250 mM. A constant electric field of −1.2 V was applied to the cell, and UV/Vis absorption spectra were recorded every 5 s (using an Agilent 8453 spectrometer). Irradiation was done via a handheld LED (*λ*_max_ = 385 nm) for a time of ca. 2 s in between each spectrum. Spectro-electrochemical data are given in Fig. 5 and Supplementary Note 9.

### General irradiation conditions

Samples were irradiated at 254 nm (UV handlamp from Herolab GmbH Laborgeräte, 254 nm, 8 W), at 365 nm (UV handlamp from Herolab GmbH Laborgeräte, 365 nm, 8 W), at 370 nm (Opulent Starboard, Luminus SST-10-UV-A130, *λ*_max_ = 370 nm, *I*_typ_ = 500 mA, *I*_max_ = 1.0 A, *Φ*_typ_ = 875 mW), at 385 nm (Opulent Starboard, Luminus SST-10-UV-A130, *λ*_max_ = 385 nm, *I*_typ_ = 500 mA, *I*_max_ = 1.5 A, *Φ*_typ_ = 1015 mW), or at 455 nm (CREE XLamp Me-C LED, *λ*_max_ = 455 nm, *I*_max_ = 700 mA). The corresponding emission spectra of each LED are given in Supplementary Fig. 24 or in the corresponding figures containing sequences of UV/Vis absorption spectra.

### Stationary UV/Vis absorption and emission spectroscopy

UV/Vis absorption (Cary 60 or Cary 100 UV-Vis spectrophotometer; Agilent) and UV/Vis emission spectra (Horiba FluoroMax-4 spectrofluorometer) were recorded at room temperature. Fluorescence quantum yields were determined with a Hamamatsu C9920-02 system equipped with a Spectralon integrating sphere. The quantum yield accuracy is <10% according to the manufacturer.

### Stepwise illumination and recording of absorption spectra

Stationary UV/Vis absorption spectra were recorded using an Agilent Cary 50 UV/Vis spectrophotometer in a sample cell of 10 × 10 mm that can be flanged to the freeze pump and thaw apparatus and closed after degassing. A sample of **5**_**red**_ was illuminated using an UV handlamp (UV handlamp from Herolab GmbH Laborgeräte, 254 nm, 365 nm, 8 W) providing either 254 or 365 nm. Continuous light was delivered onto the sample that was reproducibly positioned in ca. 2 cm distance without any further optics allowing for direct comparison of data sets recorded under different sample preparation conditions. After each illumination period, an absorption spectrum was recorded directly afterwards. The sample volume of 3 mL was continuously stirred during the illumination periods providing a homogeneously illuminated sample.

### Time-resolved UV/Vis emission spectroscopy

A self-constructed time correlated single photon counting set-up^62^ was used to record emission decay data at single detection wavelength. A quartz cuvette with four optical windows of the dimension 2 mm × 10 mm was used. The sample was excited along the 2 mm pathlength, and the emission was recorded orthogonally to this. The optical density of the sample was set to ca. 0.1 at the excitation wavelength over 2 mm pathlength.

### Ns to ms time-resolved UV/Vis absorption spectroscopy

The streak camera set-up described first in refs. ^62,63^ was adapted as follows. The third harmonic of a Nd:YAG laser (10 Hz, Surelite II, Continuum) pumping an Optical Parametric Oscillator (OPO, Continuum) tuned to 450 nm (10 mJ, ca. 10 ns) was used for sample excitation. As a probe light, a pulsed 150 W Xe-flash lamp (Applied Photophysics) was used that was focussed three times via toric mirror optics: (i) before probe shutter, (ii) into sample, and (iii) into spectrograph. The entire white light probe pulse was analysed by a combination of a spectrograph (200is, Bruker) and a streak camera (C7700, Hamamatsu Photonics). The use of mechanical shutters enabled the recording of a sequence of three individual data sets: (i) an image (*D*_FL_) with both flash lamp and laser, (ii) an image (*D*_0_) without any incoming light, and (iii) an image (*D*_F_) only with the flash lamp. One hundred such sequences were recorded, and corresponding data sets were averaged. Then the TA was calculated as:

$$\Delta {\mathrm{OD = log}}\left( {\frac{{D_{\mathrm{F}} - D_0}}{{D_{{\mathrm{FL}}} - D_0}}} \right).$$

A 10-mL sample was stepwise cycled by a peristaltic pump (ecoline, ISMATEC) through a flow cell with a pathlength of 2 mm for pump and 10 mm for probe beams (dimensions: 2 mm × 10 mm × 30 mm, Starna) ensuring a total replacement of the sample prior to each individual measurement. No photocatalyst degradation was observed under the used conditions.

### Sub-ps and sub-ns pump/supercontinuum probe spectroscopy

Ultrafast broadband TA experiments were carried out using UV/Vis pump–supercontinuum probe spectroscopy at 1 kHz repetition frequency. In short, a Ti-sapphire amplifier system (Coherent Libra) was used to generate 800 nm with 1.2 mJ pulses at 1 kHz. The output was split into three parts of which only two were used: (1) Ca. 50% of the 800 nm pulses were used to pump a collinear Optical Parametric Amplifier (OPA, TOPAS-800-fs, Light Conversion) tuned to pump pulses centred at ca. 450 nm (ca. 100 fs, ca. 400 nJ at the sample position) for sample excitation. (2) Ca. 10% were used to pump a non-colinear OPA (in-house build) tuned to pulses centred at ca. 530 nm (ca. 100 fs, ca. 5 μJ at the CaF_2_ position) for generation of supercontinuum white light probe pulses by focussing into a moving CaF_2_ disc of 1-mm thickness giving a probe spectrum ranging from 310 to 700 nm. Pump pulses were delayed via a motorised delay line equipped with an open corner cube mirror up to 2 ns. Two complementary high-speed spectrographs (Entwicklungsbüro EB Stresing) for signal and reference recording were used. The pump and probe pulses were focussed colinearly into the sample to spot sizes of ca. 80 and 60 μm full width at half maximum, respectively. For longer delays reaching out from ns to μs time ranges, a similar spectrometer was used in which the pump laser was electronically delayed relative to the probe laser. A detailed description can be found in ref. ^64^. The relative polarisations between the pump and probe were set by a half-wave plate in the pump beam path to magic angle (54.71°) for observations of pure population changes or to either parallel (0°) or orthogonal (90°) for observation of the anisotropy. The averaged pre-*t*_0_ laser scatter signal was subtracted from the data and the ca. 1 ps chirp of the white light was corrected for prior to data analysis using the coherent artefact as an indicator for time zero at each wavelength. Throughout the probe range, the spectral resolution was better than 4 nm and the temporal resolution was better than 150 fs. Ten individual scans with averaging 100 spectra per time point were typically recorded. The time axis—within total 500 points—was linear between −1 and 2 ps and logarithmic from 2 ps to the maximum time delay ensuring that the dynamics on every time scale will have equal weighting in the fitting analysis. In the sub-ps TA set-up, 10 mL of the sample were cycled through an in-house build cell with a pathlength of 100 μm for pump and probe beams. In sub-ns TA set-up, 10 mL of the sample were cycled through a flow cell (Starna) with a pathlength of 2 mm for pump and probe beams. In all cases, all scans resulted in reproducible data sets. In addition, the integrity of the sample was checked by recording stationary absorption spectra before and after each measurement. No photocatalyst degradation was observed under the used conditions. The shown data correspond to one representative measurement. No smoothing or filtering procedures were applied to the data.

### TA data analysis and modelling

Singular value decomposition (SVD)-based rank analysis and global fitting were performed using an in-house written programme described previously^62,63^. In brief, the linear least squares problem

$$\chi^{2}=\parallel{\mathbf{\Delta}} {\mathbf{A}} - {\mathbf{FB}}\parallel^{2}={\mathrm{Min}}$$

is solved, where **∆A** is the time-resolved absorption data matrix, **F** is the matrix containing the analytical functions accounting for the temporal changes in the data, i.e. exponential decays (convoluted with the instrument response, typically a Gaussian function), and **B** is the matrix with the to be determined spectra. Further optimisation of *χ*^2^ is achieved by optimising the rate constants in **F** by a nonlinear least squares algorithm. As a result of such fits, the so-called decay-associated difference spectra (in matrix **B**) and their associated optimised rate constants are obtained. These are the unique result of the global fit, and this treatment does not require any model for the kinetics involved in the transient processes. The number of exponentials in the global fit is determined by the SVD-based rank analysis, which is described elsewhere^65^. The model that relates the actual species kinetics to the elementary function is applied afterwards resulting in SAS. The shape of the SAS in terms of identity with well-known spectra or following physical laws decides about the appropriateness of the model. This step does not change the *χ*^2^ value found in the global fit, and therefore, this procedure has the advantage that all interpretation is performed with the same quality of fit.

As an alternative analysis, known species spectra, taken either from literature or recorded in this work, were taken in order to decompose the recorded time-resolved data matrix using the transpose of the data matrix and using the basis spectra instead of analytical functions. The resulting concentration–time profiles inform about the appropriateness of the basis spectra and the physical reasonability, i.e. total sum of species being constant to 1.

### Quantum chemical calculations

Quantum chemical calculations on all molecular moieties were performed using either the Firefly QC package^66^, which is partially based on the GAMESS (US)^67^ source code, or the Orca package^68,69^. All ground state structures were optimised on the level of restricted open shell density functional theory (ROHF-DFT) using the B3LYP functional and the aug-cc-pDVZ basis set. Complete active space self-consistency field (CASSCF) theory was used in order to calculate the static correlation energy. The highest 12 contributing *π* and *n* electrons were included into the CAS, distributed over 12 molecular orbitals (MO), and energy averaging over 10 states with equal weights, i.e. CASSCF(12,12)10, was performed. In order to calculate the dynamic correlation energy, extended multi-configuration quasi-degenerate perturbation theory (XMCQDPT) was used on top of the CASSCF optimised MOs^70^. In all cases, an intruder state avoidance denominator shift of 0.02 was used. The absorption spectrum for **5**_sq_ was calculated via unrestricted open shell time-dependent DFT (TD-DFT) with the aug-cc-pDVZ basis set. Solvent effects were taken into account with the conductor-like PCM for the TD-DFT calculation and with the PCM for the XMCQDPT-CASSCF calculations. Relaxed potential energy surfaces along the C-Br bond in *p*-BA and *p*-BA^•−^ were performed on the DFT (B3LYP) level of theory with the aug-cc-pVDZ basis set.

## Supplementary information

Supplementary Information

Peer Review

## Data Availability

The authors declare that the data supporting the findings of this study are available within the paper and Supplementary Information, as well as from the authors upon reasonable request.
