# Supplementary material for: Deazaflavin reductive photocatalysis involves excited semiquinone radicals
Source: Nat Commun. 2020 Jun 23;11:3174. doi: 10.1038/s41467-020-16909-y (PMC7311442; doi:10.1038/s41467-020-16909-y)
Supplement: Supplementary file 1 — Supplementary Information [file 41467_2020_16909_MOESM1_ESM.pdf]

Supplementary Information

for

Deazaflavin reductive photocatalysis involves excited semiquinone radicals

Graml *et al.*

Supplementary Note 1

Reduction of TARF to $TARFH_2$. TARF (109 mg, 0.2 mmol) was dissolved in dimethylformamide (DMF, 10 mL) and placed in a Schlenk tube. Palladium on activated charcoal (10 mg, 10% Pd) was added and the mixture was degassed by the freeze-pump-thaw method, then evacuated and flushed with hydrogen gas. The Schlenk tube was shaken until the fluorescence of **TARF** disappeared. Subsequently, palladium was removed *via* filtration through a syringe filter in a glove box. The final solution of **$TARFH_2$** was stored inside a glove box until further use.

^{1}H NMR (400 MHz, DMF-d_7) δ 10.85 (s, 1H), 10.63 (s, 1H), 6.53 (d, *J* = 8.7 Hz, 2H), 6.14 (s, 1H), 5.44 (ddd, J = 9.5, 4.2, 2.7 Hz, 1H), 5.35 (dd, *J* = 6.3, 4.2 Hz, 1H), 5.25 (td, *J* = 6.1, 3.1 Hz, 1H), 4.34 (dd, *J* = 12.3, 3.1 Hz, 1H), 4.24 – 4.10 (m, 2H), 4.01 (dd, *J* = 16.0, 2.7 Hz, 1H), 2.10 (s, 3H), 2.06 (s, 3H), 2.05 (s, 3H), 2.02 (s, 3H), 2.00 (s, 3H), 1.94 (s, 3H).

^{13}C NMR (101 MHz, DMF-d_7) δ 171.2, 170.7, 170.7, 170.6, 158.6, 150.6, 138.9, 133.4, 129.4, 128.7, 120.1, 116.7, 106.5, 71.2, 70.7, 70.5, 62.6, 48.2, 21.1, 21.0, 21.0, 20.9, 19.4, 19.3.

Chemical oxidation of 5red. 5_{red} (100 mg, 0.24 mmol, 1.0 equiv.) was dissolved in glacial acetic acid. After addition of $NaNO_2$ (33.6 mg, 0.48 mmol, 2.0 equiv.), the reaction mixture was stirred at room temperature for a few minutes, followed by addition of NaOH (15%) and extraction with dichlormethane (DCM, 3x). Subsequently, the organic layer was washed with H_2O and dried over $MgSO_4$. The product was purified by column chromatography (PE/EE) giving a yield of 80%.

General procedure: Photocatalytic reduction of aryl halides. An aryl halide (0.15 mmol, 1 equiv.), **1-5** (8 mol%), Cs_2CO_3 (49 mg, 1 equiv.), and *N*,*N*-diisopropylethylamine (DIPEA, 52 µL, 2.0 equiv.) were dissolved in the respective solvent (2 mL) and degassed using the freeze-pump-thaw technique (3 x 3 min). The reaction mixture was irradiated at either 365 nm, 385 nm, or 455 nm overnight, while continuously stirring. Analysis was done by calibrated gas chromatography using 4-methylanisole as internal standard.

Synthesis of 10-Butyl-7,8-dimethoxy-3-methyl-5-deazaisoalloxazine (1). 8 (1.24 g, 3.7 mmol) was dissolved in dry DMF (15 mL) and phosphorus trichloride (0.77 g, 5.6 mmol) was added dropwise. The reaction mixture was heated to 110°C for 30 min. After cooling down to room temperature, the yellow precipitate was filtered off and re-crystallised from a mixture of ethanol/DCM (1:2) to give 1 in a yield of 53%. Melting point 296-298°C.

^{1}H NMR (400 MHz, $CDCl_3$) δ 8.76 (Ar-*H*, s, 1H), 7.18 (s, 1H), 6.99 (Ar-*H*, s, 1H), 4.80 (*N*-CH_2, bs, 2H), 4.10 (*O*-CH_3, s, 3H), 4.01 (*O*-CH_3, s, 3H), 3.46 (*N*-CH_3, s, 3H), 1.86 (CH_2, p, *J* = 7.8 Hz, 2H), 1.56 (CH_2, h, *J* = 7.4 Hz, 2H), 1.03 (CH_3, t, *J* = 7.3 Hz, 3H).

^{13}C NMR (101 MHz, $CDCl_3$) δ 162.6 (C_q), 157.6 (C_q), 156.8 (C_q), 155.4 (C_q), 147.6 (C_q), 140.8 (+), 137.6 (C_q), 116.4 (C_q), 112.6 (C_q), 110.0 (+), 97.5 (+), 56.7(+), 56.5 (+), 44.9 (-), 29.3 (-), 28.2 (+), 20.3 (-), 14.0 (+).

HR-MS (ESI) (m/z): $[M+H]^+$ ($C_{18}H_{22}N_3O_4$) calc.: 344.1605, exp.: 344.1608.

Synthesis of 10-Butyl-7,8-dimethoxy-3,5-dimethyl-5-deazaisoalloxazine (2). 8 (333 mg, 1 mmol) was dissolved in acetic anhydride (5 mL) and heated to gentle reflux for 3 h. After cooling to room temperature, the reaction mixture was diluted with water and extracted with DCM (2 x 25 mL). The organic phase was dried over magnesium sulphate and evaporated. The crude product was recrystallised from an isopropyl alcohol/dichloroethane mixture (1:1) to give **2** in a yield of 43%. Melting point is determined to 223-226°C.

^{1}H NMR (400 MHz, $CDCl_3$) δ 7.43 (Ar-*H*, s, 1H), 6.98 (Ar-*H*, s, 1H), 4.80 (*N*-C*H₂*, bs, 2H), 4.09 (*O*-C*H₃*, s, 3H), 4.03 (*O*-C*H₃*, s, 3H), 3.44 (*N*-C*H₃*, s, 3H), 3.23 (C*H₃*, s, 3H), 1.83 (C*H₂*, t, *J* = 8.2 Hz, 2H), 1.54 (C*H₂*, p, *J* = 7.4 Hz, 2H), 1.03 (C*H₃*, t, *J* = 7.3 Hz, 3H).

^{13}C NMR (101 MHz, $CDCl_3$) δ 157.1 (C_q), 156.1 (C_q), 155.3 (C_q), 154.9 (C_q), 147.1 (C_q), 136.1 (C_q), 117.6 (C_q), 110.8 (C_q), 110.1 (C_q), 106.7 (+), 97.6 (+), 56.5 (+), 56.4 (+), 45.2 (-), 29.3 (-), 28.2 (+), 20.3 (-), 17.1 (+), 14.1 (+).

HR-MS (ESI) (m/z): $[M+H]^+$ ($C_{19}H_{24}N_3O_4$) calc.: 358.1761, exp.: 358.1768.

Synthesis of 10-Butyl-7,8-dimethoxy-5-isopropyl-3-methyl-5-deazaisoalloxazine (3). 8 (333 mg, 1 mmol) was dissolved in butyric anhydride (4 mL) and heated to 180°C for 4 h. After cooling to room temperature, the reaction mixture was diluted with water and extracted with DCM (2 x 25 mL). The organic phase was dried over magnesium sulphate and evaporated. The crude product was recrystallised from isopropyl alcohol to give **3** in a yield of 57%. Melting point is determined to 171-175°C.

^{1}H NMR (400 MHz, $CDCl_3$) δ 7.72 (Ar-*H*, s, 1H), 6.98 (Ar-*H*, s, 1H), 5.50 – 5.35 (CH, m, 1H), 4.78 ($N-CH_2$, s, 2H), 4.07 ($O-CH_3$ s, 3H), 4.00 (CH_3, s, 3H), 3.45 ($N-CH_3$, s, 3H), 1.84 (CH_2, d, *J* = 8.5 Hz, 2H), 1.63 (CH_3, s, 3H), 1.61 (CH_3, s, 3H), 1.55 (CH_2, q, *J* = 7.5 Hz, 2H), 1.03 (CH_3, t, *J* = 7.3 Hz, 3H).

^{13}C NMR (101 MHz, $CDCl_3$) δ 165.4 (C_q), 156.9 (C_q), 155.5 (C_q), 154.9 (C_q), 145.8 (C_q), 136.8 (C_q), 116.5 (C_q), 111.2 (C_q), 109.0 (+), 97.7 (+), 56.4 (+), 56.4 (+), 45.5 (-), 29.2 (-), 28.4 (+), 28.4 (+), 22.2 (+), 20.3 (-), 14.1 (+).

HR-MS (ESI) (m/z): $[M+H]^+$ ($C_{21}H_{28}N_3O_4$) calc.: 386.2074, exp.: 386.2076.

Synthesis of 10-Butyl-7,8-dimethoxy-3-methyl-5-trifluoromethyl-5-deazaisalloxazine (4). 8 (333 mg, 1 mmol) was dissolved in *N*-methyl-2-pyrrolidone (3 mL) and trifluoroacetic anhydride (630 mg, 3 mmol) was added. The reaction mixture was heated to 150°C for 2 h. After cooling to room temperature, the reaction mixture was diluted with water and extracted with DCM (2 x 25 mL). The organic phase was dried over magnesium sulphate and evaporated. The crude product was re-crystallised from isopropyl alcohol to give **4** in a yield of 63%. Melting point is determined to 256-261°C.

^{1}H NMR (400 MHz, $CDCl_3$) δ 7.48 (Ar-*H*, q, *J* = 1.8 Hz, 1H), 7.00 (Ar-*H*, s, 1H), 4.80 (*N*-C*H₂*, bs, 2H), 4.10 (*O*-C*H₃*, s, 3H), 4.00 (*O*-C*H₃*, s, 3H), 3.44 (*N*-C*H₃*, s, 3H), 1.94 – 1.82 (C*H₂*, m, 2H), 1.56 (C*H₂*, h, *J* = 7.4 Hz, 2H), 1.04 (C*H₃*, t, *J* = 7.4 Hz, 3H).

^{13}C NMR (101 MHz, $CDCl_3$) δ 160.7 (C_q), 156.8 (C_q), 156.7 (C_q), 154.1 (C_q), 147.6 (C_q), 139.2 (q, *J* = 33.1 Hz), 137.8 (C_q), 122.6 (q, *J* = 279.3 Hz), 114.0 (C_q), 113.3 (C_q), 107.1 (q, *J* = 5.9 Hz), 97.4 (+), 56.7(+), 56.4 (+), 46.2 (-), 29.0 (-), 28.6 (+), 20.3 (-), 14.0 (+).
^{19}F NMR (282 MHz, $CDCl_3$) δ -53.72 (d, *J* = 1.9 Hz).
HR-MS (ESI) (m/z): $[M+H]^+$ ($C_{19}H_{21}N_3O_4F_3$) calc.: 412.1479, exp.: 412.1483.

Synthesis of 10-butyl-7,8-dimethoxy-3-methyl-5-phenyl-5-deazaisoalloxazine (5), 10-butyl-7,8-dimethoxy-3-methyl-5-phenyl-5,10-dihydropyrimido[4,5-b] quinoline-2,4(1H,3H)-dione (5_{red}). The procedure has been adapted from the synthesis of similar structures described by Shi *et al.*.[1] A microwave vessel was charged with *N*-butyl-3,4-dimethoxyaniline (**7**, 2.1 g, 10 mmol, 1.0 equiv.), *N*-methylbarbituric acid (**9**, 1.42 g, 10 mmol, 1.0 equiv.), and benzaldehyde (10 mL, 10 mmol, 1.0 equiv.). Without addition of a solvent the mixture was heated in a microwave to 140°C for 30 min. Subsequently, the solid reaction mixture was dissolved in DCM and extracted with H_2O. The oxidised and reduced forms were purified and separated by column chromatography, followed by recrystallisation from ethylacetate (**5_{ox}**) and from toluene (**5_{red}**), respectively. The products were obtained in a combined yield of 20%.
(**5_{ox}**) = **^{1}H NMR** (400 MHz, $CDCl_3$) δ [ppm] = 7.60 – 7.50 (Ar-*H*, m, 3H), 7.24 – 7.17 (Ar-*H*, m, 2H), 7.02 (Ar-*H*, s, 1H), 6.63 (Ar-*H*, s, 1H), 4.89 (C*H_2*, s, 2H), 4.09 (*O*-C*H_3*, s, 3H), 3.62 (*O*-C*H_3*, s, 3H), 3.30 (*N*-C*H_3*, s, 3H), 1.92 (C*H_2*, m, 2H), 1.60 (C*H_2*, m, 2H), 1.07 (C*H_3*, t, *J* = 7.3 Hz, 3H).
^{13}C NMR (101 MHz, $CDCl_3$) δ [ppm] = 162.0 (C_q), 157.2 (C_q), 156.3 (C_q), 155.5 (C_q), 155.1 (C_q), 146.8 (C_q), 136.9 (C_q), 136.8 (C_q), 128.4 (+), 128.3 (+), 127.0 (+), 117.9 (C_q), 109.7 (C_q), 109.4 (+), 97.1 (+), 56.5 (+), 55.9 (+), 45.3 (-), 29.1 (-), 28.0 (+), 20.3(-), 14.0 (+).
HRMS (ESI) (m/z): $[M + H]^+$ ($C_{24}H_{25}N_3O_4$) calc.: 420.1918, exp.: 420.1923.

(**5_{red}**) = **^{1}H NMR** (400 MHz, $CDCl_3$) δ [ppm] =10.69 (N*H*, s, 1H), 7.25 – 7.17 (Ar-H, m, 4H), 7.13 (Ar-*H*, m, 1H), 6.64 (Ar-*H*, d, *J* = 1.4 Hz, 2H), 5.26 (C*H*, s, 1H), 4.02 (C*H_2*, m, 2H), 3.90 (*O*-C*H_3*, s, 3H), 3.78 (*O*-C*H_3*, s, 3H), 3.31 (*N*-C*H_3*, s, 3H), 1.87 – 1.61 (C*H_2*, m, 2H), 1.44 (C*H_2* m, 2H), 0.97 (C*H_3* t, *J* = 7.3 Hz, 3H).
^{13}C NMR (101 MHz, $CDCl_3$) δ [ppm] = 162.4 (C_q), 152.8 (C_q), 148.3 (C_q), 146.5 (C_q), 146.1 (C_q), 144.9 (C_q), 131.2 (C_q), 128.4 (+), 127.4 (+), 126.5 (+), 119.5 (C_q), 112.9 (+), 99.9 (+), 90.1 (C_q), 56.4 (+), 56.2 (+), 44.1 (-), 40.3 (+), 30.0 (-), 27.3 (+), 20.1 (-), 13.9 (+).
HRMS (ESI) (m/z): $[M + H]^+$ ($C_{24}H_{27}N_3O_4$) calc.: 422.2074, exp.: 422.2077.

Synthesis of the flavin precursor *N*-Butyl-3,4-dimethoxyaniline (7). The procedure has been adapted from the synthesis of similar structures described by Abdel-Magid *et. al.*.[2] 3,4-dimethoxyaniline (1.53 g, 10 mmol, 1.0 equiv.) and butanal (901 µL, 10 mmol, 1.0 equiv.) were mixed in 35 mL DCE and then treated with sodium triacetoxyborohydride (2.97 g, 14 mmol, 1.4 equiv.). The mixture was stirred at room temperature for 17 h and the reaction progress was observed *via* TLC. After quenching of the reaction mixture with $NaHCO_3$ (*aq.*), the product was extracted with ethyl acetate and the organic phase was dried over magnesium sulfate. After filtration the ethyl

acetate was evaporated and the crude product was purified *via* column chromatography (PE/EE). The product was obtained in a yield of 52%.

^{1}H NMR (300 MHz, $CDCl_3$) δ 6.72 (Ar-*H*, d, *J* = 8.5 Hz, 1H), 6.22 (Ar-*H*, d, *J* = 2.7 Hz, 1H), 6.12 (Ar-*H*, dd, *J* = 8.5, 2.6 Hz, 1H), 3.80 (*O*-C*H*$_3$, s, 3H), 3.77 (*O*-C*H*$_3$, s, 3H), 3.27 (N*H*, s, 1H), 3.04 (C*H*$_2$, t, *J* = 7.1 Hz, 2H), 1.57 (C*H*$_2$, m, 2H), 1.42 (C*H*$_2$, m, 2H), 0.94 (C*H*$_3$, t, *J* = 7.3 Hz, 3H).

^{13}C NMR (75 MHz, $CDCl_3$) δ 150.0 (C_q), 143.6 (C_q), 141.3 (C_q), 113.3 (+), 103.4 (+), 98.8 (+), 56.7 (+), 55.7 (+), 44.5 (-), 31.8 (-), 20.3 (+), 14.0 (-).

Synthesis of the flavin precursor 6-(*N*-Butyl(3,4-dimethoxyphenyl)amino)-3-methyluracil (8). Aniline **7** (13.1 g, 62.5 mmol), 6-chloro-3-methyl uracil (10.0 g, 62.5 mmol) and *N,N*-dimethylaniline (30 mL) were heated to 180°C for 4 h under inert atmosphere. After cooling to *ca.* 100°C, the reaction mixture was slowly poured to diethyl ether (350 mL). After several minutes a thick dark oil precipitated. The diethyl ether layer was transferred into a clean flask and kept at 4 °C in a refrigerator for 24 h. The crude product was filtered, washed with diethyl ether and subsequently re-crystallised from hot isopropyl alcohol to give **8** as a white crystalline powder in a yield of 55%.

^{1}H NMR (300 MHz, $CDCl_3$) δ 7.52 (N*H*, s, 1H), 6.89 (Ar-*H*, d, *J* = 8.5 Hz, 1H), 6.74 (Ar-*H*, dd, *J* = 8.5, 2.4 Hz, 1H), 6.63 (Ar-*H*, d, *J* = 2.4 Hz, 1H), 4.95 (C*H*, d, *J* = 2.3 Hz, 1H), 3.90 (*O*-C*H*$_3$, s, 3H), 3.86 (*O*-C*H*$_3$, s, 3H), 3.54 – 3.39 (C*H*$_2$, m, 2H), 3.20 (*N*-C*H*$_3$, s, 3H), 1.59 (C*H*$_2$, m, 2H), 1.29 (C*H*$_2$, m, 2H), 0.89 (C*H*$_3$, t, *J* = 7.3 Hz, 3H).

^{13}C NMR (75 MHz, $CDCl_3$) δ 164.4 (C_q), 151.2 (C_q), 151.0 (C_q), 150.6 (C_q), 149.6 (C_q), 132.3 (C_q), 120.8 (+), 112.0 (+), 111.2 (+), 76.3 (+), 56.3 (+), 52.5 (-), 29.0 (-), 26.8 (+), 20.1 (-), 13.9 (+).

HRMS (ESI) (m/z): $[M + H]^+$ ($C_{17}H_{24}N_3O_4$) calc.: 334.1761, exp.: 334.1769

Synthesis of the flavin precursor *N*-methylbarbituric acid (9). *N*-methyl urea (11.1 g, 150 mmol, 1.0 equiv.) and malonic acid (15.6 g, 150 mmol, 1.0 equiv.) were dissolved in 40 mL acetic anhydride and heated to 70°C for 2 h. Subsequently, the acetic acid was evaporated and the residue stored at -40°C overnight. The precipitate was filtered and washed with cold ethanol. Purification was done by re-crystallization from ethanol and the product was obtained as an orange solid with a yield of 45%.

^{1}H NMR (300 MHz, DMSO-d_6) δ 11.33 (N*H*, s, 1H), 3.58 (C*H*$_2$, s, 2H), 3.05 (C*H*$_3$, s, 3H).

^{13}C NMR (75 MHz, DMSO-d_6) δ 166.9 (C_q), 166.4 (C_q), 151.8 (C_q), 39.6 (+), 26.7 (+).

Supplementary Note 2

Photophysical properties and the photophysical model. Stationary absorption and emission spectra in the UV/Vis range were recorded in ACN for all synthesised photocatalysts (Supplementary Figure 1 panels **a**, **c**, **e**, **g**, and **i**). Additionally, the emission decay was recorded probing at the maximum of the corresponding emission wavelength (Supplementary Figure 1 panels **b**, **d**, **f**, **h**, and **j**). For all oxidised species the emission decay is mono-exponential indicating clean excited singlet state decays *via* $k_{S_1} = k_{ic} + k_{rad} + k_{isc}$. Thus, measuring the fluorescence quantum yields of the S_1 emission of all oxidised species allowed the determination of k_{rad} *via* $k_{rad} = \Phi_{rad} k_{S_1}$. The triplet formation was recorded *via* transient absorption spectroscopy. Interestingly, only for compound $\mathbf{5_{ox}}$ we observed the formation of its triplet state (Figure 4 in the main text)**.** Its yield can be estimated as follows: For **1-5** alone in solution, we only observe the photophysical processes but no photochemistry. Thus, the rate constants κ_i, which are absolutely obtained from a bi-exponential global fit on the data, represent

$$\kappa_1 = k_{ic} + k_{rad} + k_{isc} \quad (1)$$

$$\kappa_2 = k_{bisc} \quad (2)$$

In this simple model considering the ground state contribution, S_{S_0}, one obtains the following relationship between the SAS, S_i, and the DADS, D_i:

$$S_{S_1} = \frac{(D_1 + D_2)}{c_0} + S_{S_0} \quad (3)$$

$$S_{T_1} = \frac{(\kappa_1 - \kappa_2) D_2}{c_0 \Phi_{T_1} \kappa_1} + S_{S_0} \quad (4)$$

As can be seen, there are undetermined parameters c_0 and Φ_{T_1}. However, one can at least find upper or lower bounds by the requirement that the resulting SAS must be positive, and should not show any of the characteristic bands of the other species. In particular the negative peaks from the ground state bleach should disappear in the SAS. The best values are summarised in Supplementary Table 1 and the corresponding SAS are shown in Figures 3**d** and 4**e** of the main text.

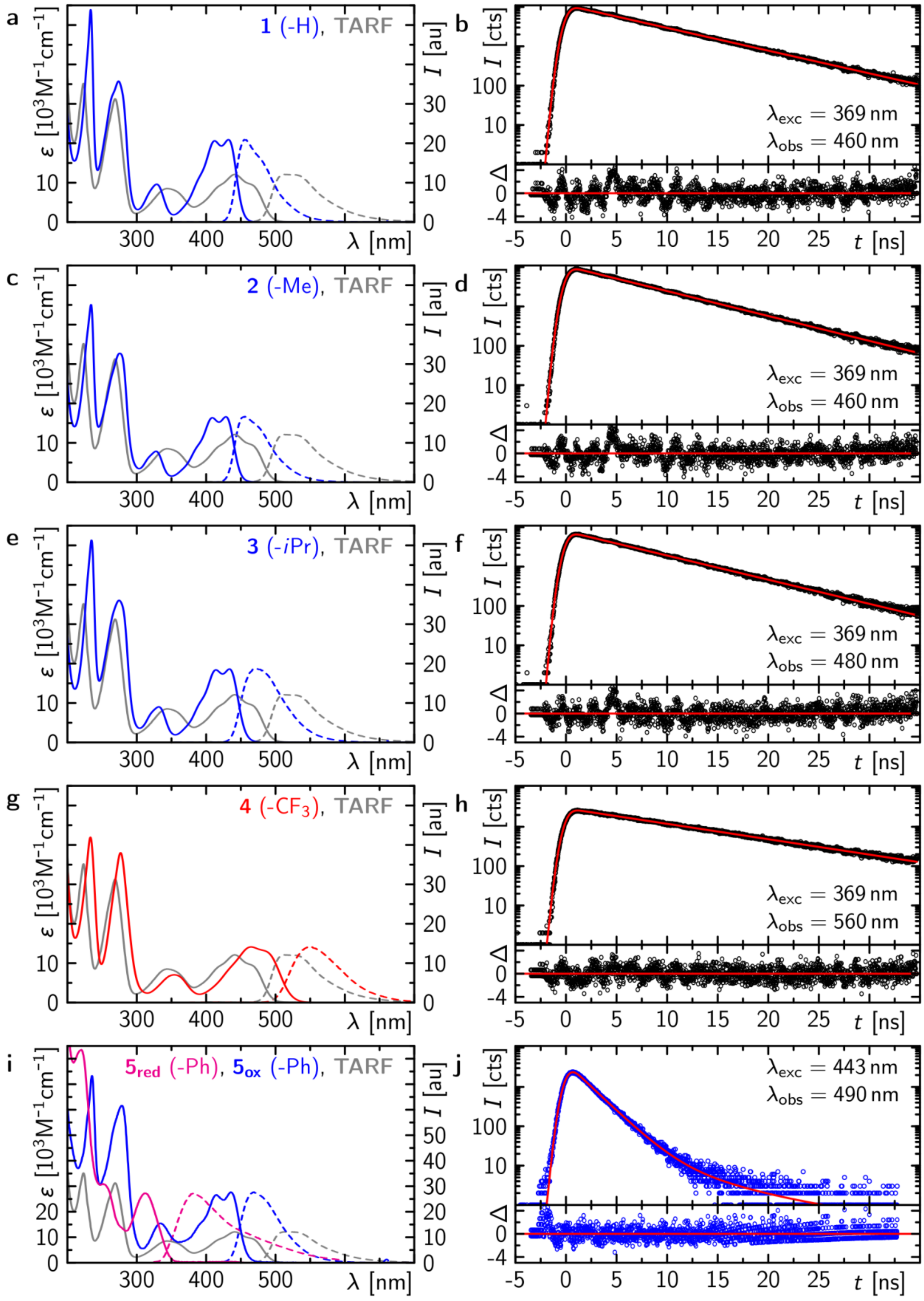

Supplementary Figure 1. **Photophysical characterisation of deazaflavins.** Stationary absorption and emission spectra as well as emission decay curves of the photocatalysts **1** (**a**-**b**), **2** (**c**-**d**), **3** (**e**-**f**), **4** (**g**-**h**), and **5** (**i**-**j**) in ACN as indicated. In case of **5**, also the spectra and decay curves of its fully reduced form $\mathbf{5_{red}}$ were recorded (magenta lines in **i**). **TARF** spectra are also presented as reference in grey.

Supplementary Table 1. Summary of the photophysical parameters of deazaflavins in ACN.

Cat.	k_{S_1}	$\Phi_{fl}{}^{a}$	$k_{rad}{}^{b}$	$\Phi_{isc}{}^{c}$	$k_{isc}{}^{d}$	$\Phi_{ic}{}^{e}$	$k_{ic}{}^{f}$	$k_{T_1}^{O_2\,g}$
	[(ns)$^{-1}$]		[(ns)$^{-1}$]		[(ns)$^{-1}$]		[s^{-1}]	[(μs)$^{-1}$]
**1**	0.132±0.002	0.85	0.112	$\ll 1$	≈ 0	0.15	0.0198	no
**2**	0.146±0.002	0.71	0.104	nd	nd	nd	nd	nd
**3**	0.141±0.002	0.68	0.096	nd	nd	nd	nd	nd
**4**	0.090±0.002	0.67	0.060	nd	nd	nd	nd	nd
$\mathbf{5_{ox}}$	0.573±0.002	0.16	0.092	0.11	0.063	0.73	0.418	6.45
$\mathbf{5_{red}}$	0.296±0.002	0.03	0.0089	nd	nd	nd	nd	nd
**TARF**	0.149±0.002	0.39	0.058	0.45	0.067	0.16	0.024	2.10

[a]The accuracy is < 10% according to the manufacturer.

[b] $k_{rad}=(\tau_{S_1})^{-1}\Phi_{fl}$.

[c] from spectra modelling.

[d] $k_{isc}=(\tau_{S_1})^{-1}\Phi_{isc}$.

[e] $\Phi_{ic}=1-\Phi_{fl}-\Phi_{isc}$.

[f] $k_{ic}=(\tau_{S_1})^{-1}\Phi_{ic}$.

[g] in non-degassed acetonitrile with expected c(O_2) = 2.4 mM.[2]

Abbreviations: S_1 = excited singlet state, T_1 = triplet state, fl = fluorescence, rad = radiative, isc = intersystem crossing, ic = internal conversion, no = not observed, nd = not determined.

Supplementary Note 3

Stability of 5_{red} against O_2 and light. The stability of freshly prepared $\mathbf{5_{red}}$ samples in ACN was tested by recording UV/Vis absorption spectra over time under the following three conditions: 1) in non-degassed ACN in the dark, 2) in non-degassed ACN and excitation of $\mathbf{5_{red}}$, and 3) in degassed ACN and excitation of $\mathbf{5_{red}}$. In the first experiment, spectra were recorded in the dark over a period of 19 h. As shown in Supplementary Figure 2, no significant reduction of $\mathbf{5_{red}}$ and correspondingly no formation of $\mathbf{5_{ox}}$ is observed. Thus, the oxidation rate of the ground state $\mathbf{5_{red}}$ is $\ll$ 6.1 10^{-3} $M^{-1}s^{-1}$ considering a O_2 concentration of 2.4 mM in ACN[3] under atmospheric conditions. Correspondingly, the activation energy barrier is $\gg k_BT$.

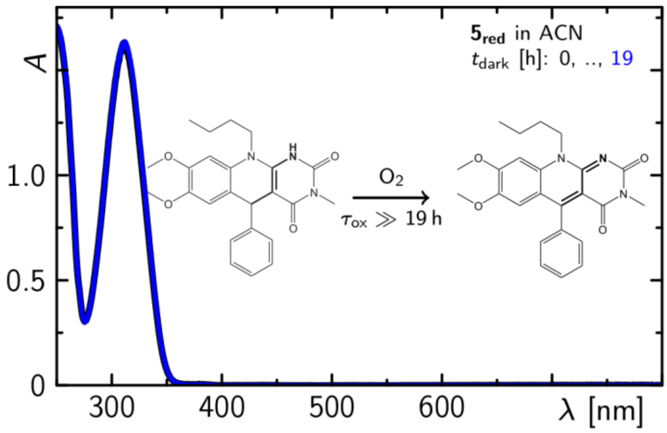

Supplementary Figure 2. **Stability of 5_{red} towards molecular oxygen.** Sequence of stationary UV/Vis absorption spectra of a freshly prepared sample of $\mathbf{5_{red}}$ in ACN recorded over 19 h incubation in the dark as indicated.

The situation is different when $\mathbf{5_{red}}$ is excited. Excitation of $\mathbf{5_{red}}$ in O_2 saturated ACN at either 254 nm (Supplementary Figure 3 panels **a** and **b**) or 365 nm (Supplementary Figure 3 panels **c** and **d**) resulted in a clean conversion into its fully oxidised form $\mathbf{5_{ox}}$. However, the conversion was not complete, since the oxidised form also absorbs substantially in the UV region. In case of excitation at 254 nm, the system reaches the photo-stationary point under the used conditions within 150 s. Under otherwise identical geometrical illumination conditions but with an excitation spectrum peaking at 365 nm, which has much less overlap with the absorption spectrum of $\mathbf{5_{red}}$ and is 500 times as intensive compared to the 254 nm spectrum of the excitation source, the photo-stationary point is not reached even after 2600 s. However, significant conversion is still observed. Considering the fact that $\mathbf{5_{red}}$ in O_2 saturated ACN is stable for days in the dark (Supplementary Figure 2), this provides proof of a light-induced re-oxidation. The only potential oxidant present in the system is O_2. This should be proofed by a complete inhibition of the re-oxidation or at least a significantly reduced turnover rate when O_2 is removed or reduced in concentration from the system. As can be readily seen in Supplementary Figure 3 panels **e** and **f** the turnover rate of $\mathbf{5_{red}}$ is indeed significantly reduced as the photo-stationary point is reached only after 4000 s compared to 150 s when O_2 is present. Under the used conditions for our freeze-pump-thaw apparatus, we estimate a reduction of the O_2 concentration only down to the μM

range, which explains the observation of almost quantitative conversion under these conditions. To note, under reduced O_2 concentration conditions illumination also leads to decomposition pathways that become available when oxidation by O_2 is significantly reduced. However, we do not want to address these decomposition pathways in this work.

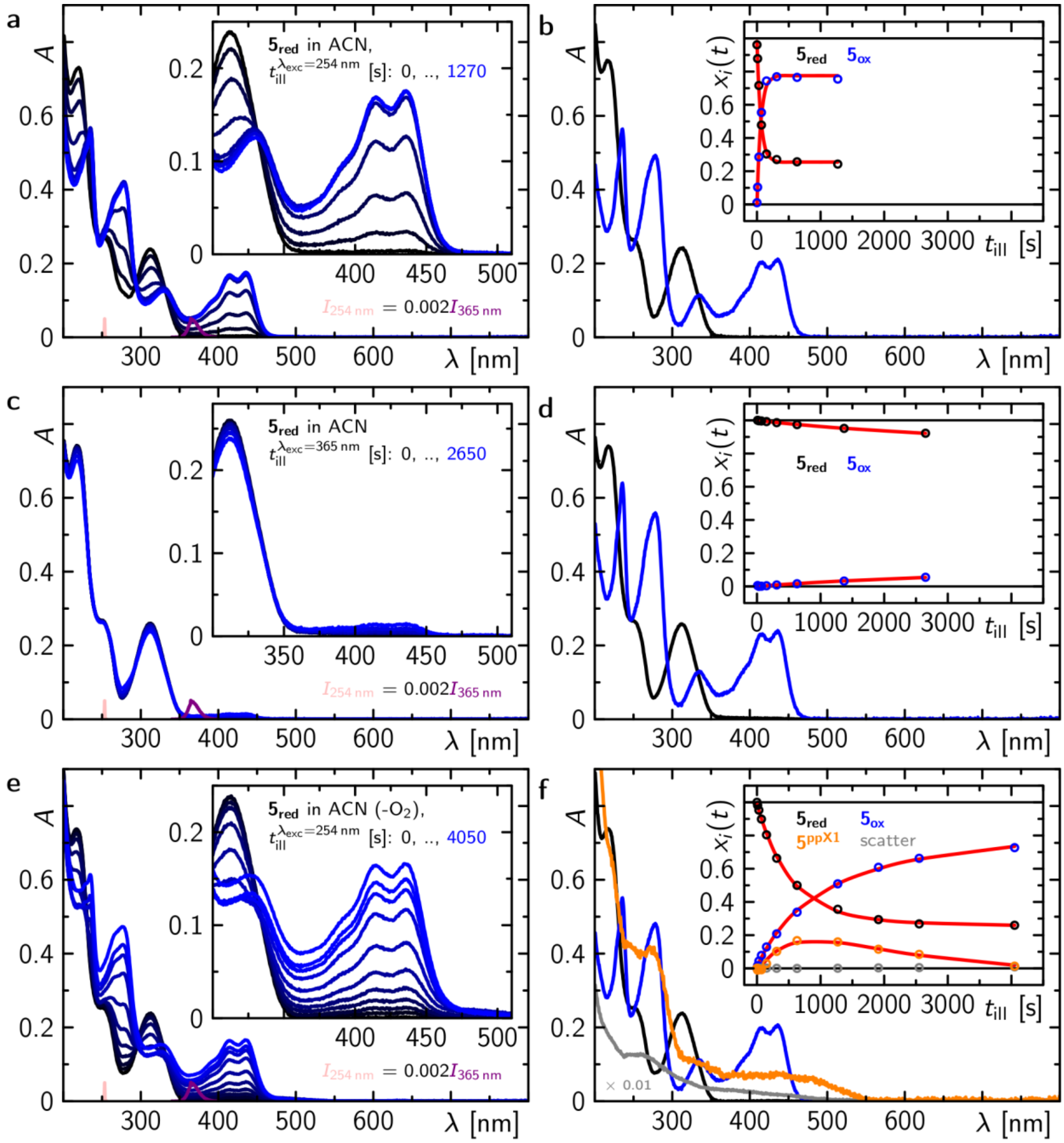

Supplementary Figure 3. Photooxidation of $\mathbf{5_{red}}$. Sequences of stationary absorption spectra of $\mathbf{5_{red}}$ in non-degassed (**a**-**d**) ACN and in degassed (**e**-**f**) ACN after stepwise illumination at wavelengths as indicated under identical geometrical illumination conditions. The panels on the left (**a**, **c**, and **e**) show the raw data. The corresponding panels on the right (**b**, **d**, and **f**) show the decomposed species spectra and corresponding concentration-time profiles (insets).

Supplementary Note 4

Electrochemical characterisation. Cyclic voltammograms for **TARF**, **1**, **2**, **3**, **4**, and **5** in ACN were recorded against ferrocene (Supplementary Figure 4). The corresponding redox potentials were calculated as follows: potential of sample against electrode minus potential of reference system against electrode plus correction of +0.38 V for conversion to the potential against saturated calomel electrode (SCE). These values are summarised in Supplementary Table 2.

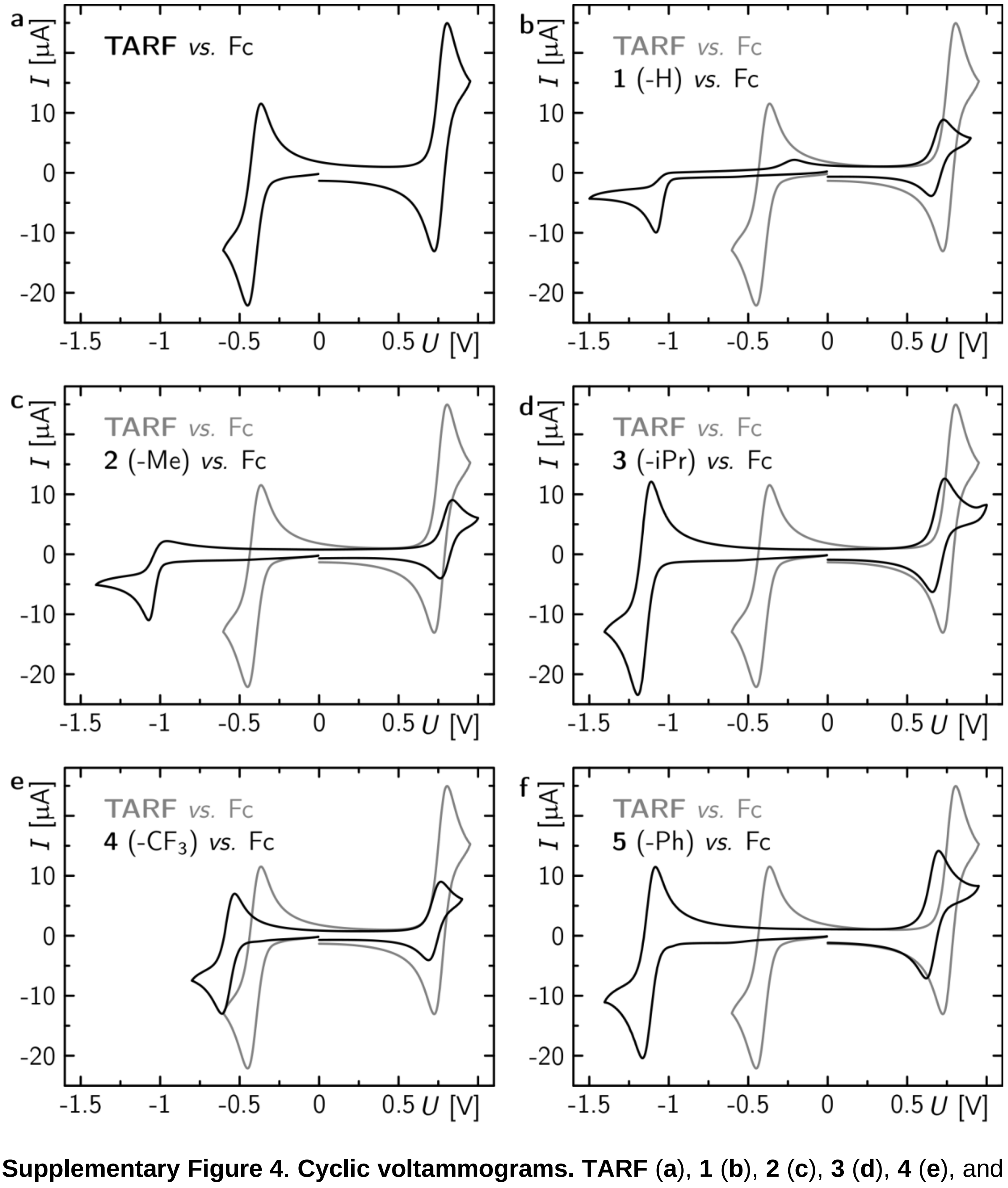

Supplementary Figure 4. **Cyclic voltammograms. TARF** (**a**), **1** (**b**), **2** (**c**), **3** (**d**), **4** (**e**), and **5** (**f**) in ACN *vs.* ferrocene as indicated.

Supplementary Table 2. Calculated $E_{1/2}$ values for TARF, 1, 2, 3, 4, and 5 in ACN.

Catalyst	$E_{1/2}$ [V]
**TARF**	-0.79
**1**	-1.34
**2**	-1.44
**3**	-1.45
**4**	-0.92
**5**	-1.41

Supplementary Note 5

Screening of the reaction conditions. Supplementary Table 3 summarises the results of the investigations on the influence of the solvent and the excitation wavelength on the total product yield.

Supplementary Table 3. **Screening of the reaction conditions**. Photocatalytic dehalogenation of *p*-bromoanisole or *p*-chloroanisole by deazaflavin in dependence on the solvent and the excitation wavelength for $\mathbf{5_{ox}}$ in ACN.

c(**5**) [mM]	*t* [h]	solvent	Cs_2CO_3 [equiv.]	λ_{exc} [nm]	DIPEA [mM]	Substrate ***p*-XA**	product yield[b]
6	16	DMF	**0**	365	150	Br	**29**
6	16	ACN	**0**	365	150	Br	**55**
6	16	**DMF**	1	365	150	Br	**44**
6	16	**ACN**	**1**	365	150	Br	**80**
6	16	ACN	**0.1**	365	150	Br	**58**
6	18	ACN	1	**455**	150	Br	**50**
6	18	ACN	1	**385**	150	**Br**	**80**
**6**	18	ACN	1	**365**	150	Br	**78**
**3**	18	ACN	1	365	150	Br	**60**
**0.75**	18	ACN	1	365	150	Br	**33**
**0**	16	ACN	1	365	150	Br	**5**
6	16	ACN	1	**dark**	150	Br	**trace**
3	16	ACN	1	385	**0**	Br	**3**
3	16	ACN	1	385	**0**	Br	**trace**
6	15	ACN	1	385	150	**Cl**	**80**

[a]Reaction conditions: ***p*-XA** (75 mM (0.15 mmol)), DIPEA (150 mM (0.3 mmol)), ACN (2 mL), 25°C, nitrogen atmosphere.
[b]Yields determined *via* calibrated GC-analysis with 4-methylanisole as internal standard.

Supplementary Note 6

Theoretical absorption spectra of all protonation states of $\mathbf{5_{sq}}$. The total photocatalytic conversion from ***p*-BA** to ***p*-A** (anisole) shows a dependence on the used solvent, the used excitation wavelength, and the basicity. Since the semiquinone form, $\mathbf{5_{sq}}$, of the photocatalyst is one key intermediate for the successful dehalogenation, we

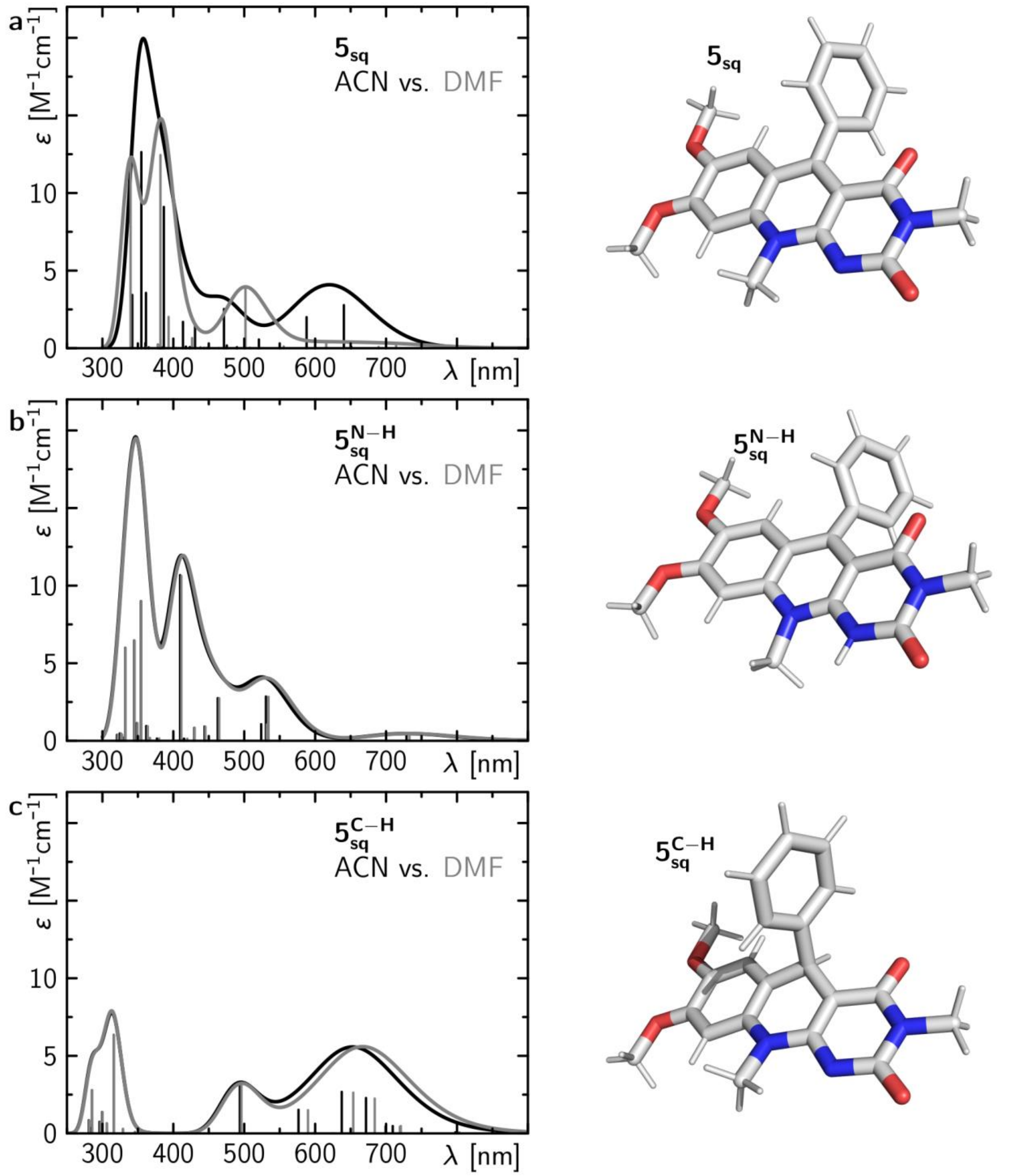

Supplementary Figure 5. **Calculated electronic absorption spectra of $\mathbf{5_{sq}}$ radicals.** Electronic transitions (sticks) for all possible protonation states of $\mathbf{5_{sq}}$ (**a**-**c**) convoluted with Gaussians (FWHM = 0.2 eV) calculated at the UHF-TD-DFT(B3LYP)-CPCM(ACN,DMF)//aug-cc-pVDZ level of theory. The corresponding optimised structures are on the right.

calculated quantum chemically absorption spectra of $\mathbf{5_{sq}}$ and its two potential protonation states, *i.e.* $\mathbf{5_{sq}^{C\text{-}H}}$ and $\mathbf{5_{sq}^{N\text{-}H}}$ in PCM for ACN and DMF (Supplementary Figure 5). As evident, the theoretical and experimental absorption spectra of $\mathbf{5_{sq}}$ in ACN are in excellent agreement (Supplementary Note 9, Supplementary Figure 5**a** and Figure 4**f** in the main text). Considering the significant deviations of the absorption spectra of both protonated forms (Supplementary Figure 5 panels **b** and **c**) from the experimentally recorded transient spectrum (Figure 4**f** in the main text and Supplementary Note 9) in the absence of base, we can already exclude the involvement of these species under neutral conditions and, thus, identify $\mathbf{5_{sq}}$ as the key intermediate of the consecutive photo-induced electron transfer (conPET) reaction. Furthermore, this is in accord with the observation that with excitation wavelength < 400 nm the total conversion under otherwise identical reaction conditions increased by a factor of 1.6, since $\mathbf{5_{sq}}$ has its highest absorption probability at around 360 nm. Moreover, the calculation shows that the most intense transitions around 365 nm of the $\mathbf{5_{sq}}$ in DMF shift apart resulting in an expected overall decreased extinction coefficient compared to the situation in ACN (Supplementary Figure 5**a**). Therefore, this might explain the observed decreased conversion yield by a factor of *ca.* 2 in DMF (Supplementary Table 3).

In order to get more insights on the impact of the basicity on the reaction mechanism further studies are currently ongoing in our lab. At present, the working hypothesis explaining the observed enhanced yields under basic conditions are given by the following possible scenarios: 1) enhanced triplet yield; 2) longer triplet lifetime; 3) enhanced $\mathbf{dFl_{sq}}$ yield; or 4) longer lifetime of $\mathbf{dFl_{sq}}$. However, an enhancement of the final dehalogenation step after initiation by the second electron transfer can already be excluded since this part of the reaction is identical, thus, independent on the tested deazaflavin.

Supplementary Note 7

Testing the excited singlet state reactivity. The Smoluchowski theory[4,5] is used in order to quantify, on the one hand, the diffusion limits for a bi-molecular reaction and, on the other hand, its efficiency on encounter of the reacting species. In accordance to the Smoluchowski theory, one would not expect to observe a mono-exponential kinetic behaviour for bi-molecular reactions under pseudo-first order conditions, but instead an $e^{\sqrt{t}}$ dependence. The diffusion-controlled quenching rate using the sum of molecular radii $R = R_{0,A} + R_{0,B}$ and the sum of diffusion constants $D = D_A + D_B$ of two reacting species A and B is given by

$$k_{\text{diff}}(t) = 4\,\pi\,R\,D\,N_A\,p\left(1 + \frac{p\,R}{\sqrt{\pi\,D\,t}}\right) \tag{5}$$

Here, p is a factor for the interaction probability upon encounter of the photocatalyst and the substrate. In case $p = 1$, the reaction is totally diffusion controlled. According to the Stokes-Einstein relation the diffusion coefficient of a molecule can be estimated to:

$$D = \frac{k_B T}{6\,\pi\,\eta\,R_{0,\text{molecule}}} \tag{6}$$

where $R_{0,\text{molecule}}$ might be estimated as the radius of a sphere with the molecular volume. The molecular volume itself might be estimated by the volume enclosed by the solvent-excluded surface (SES). Strategies to determine the SES might be found in.[6] Supplementary Table 4 summarises the corresponding parameters for the description of diffusion controlled processes taking ACN as solvent, which has a viscosity of $3.5\cdot10^{-5}$ kg·(dm)$^{-1}$·s^{-1} at a temperature of $T = 293.15$ K.[7] In case of diffusion-controlled quenching of the excited singlet states of **1**$_{\mathbf{ox}}$ and **5**$_{\mathbf{ox}}$ by DIPEA due to electron transfer from DIPEA to the excited singlet state, the non-linear contribution

Supplementary Table 4. Parameter for the Smoluchowski model. The diffusion coefficients are for ACN at 293.15 K.

Cat/Sub	$r_{0,\text{Cat}}$ [dm]	$r_{0,\text{Sub}}$ [dm]	D_{Cat} [(dm)^{2}s^{-1}]	D_{Sub} [(dm)^{2}s^{-1}]	$4\,\pi\,r_0 D N_A$ [M^{-1}s^{-1}]	$\frac{r_0}{\sqrt{\pi\,D}}$ $\left[\frac{1}{\sqrt{s^{-1}}}\right]$
**TARF**/ DIPEA	$4.78\cdot10^{-9}$	$3.21\cdot10^{-9}$	$1.28\cdot10^{-7}$	$1.91\cdot10^{-7}$	$1.93\cdot10^{10}$	$7.99\cdot10^{-6}$
**1**/ DIPEA	$4.12\cdot10^{-9}$	$3.21\cdot10^{-9}$	$1.49\cdot10^{-7}$	$1.91\cdot10^{-7}$	$1.89\cdot10^{10}$	$7.10\cdot10^{-6}$
**5**$_{\mathbf{ox}}$/ DIPEA	$4.45\cdot10^{-9}$	$3.21\cdot10^{-9}$	$1.38\cdot10^{-7}$	$1.91\cdot10^{-7}$	$1.91\cdot10^{10}$	$7.54\cdot10^{-6}$
**TARF**/ O_2	$4.78\cdot10^{-9}$	$1.59\cdot10^{-9}$	$1.28\cdot10^{-7}$	$3.86\cdot10^{-7}$	$2.48\cdot10^{10}$	$5.01\cdot10^{-6}$
**1**/ O_2	$4.12\cdot10^{-9}$	$1.59\cdot10^{-9}$	$1.49\cdot10^{-7}$	$3.86\cdot10^{-7}$	$2.31\cdot10^{10}$	$4.41\cdot10^{-6}$
**5**$_{\mathbf{ox}}$/ O_2	$4.45\cdot10^{-9}$	$1.59\cdot10^{-9}$	$1.38\cdot10^{-7}$	$3.86\cdot10^{-7}$	$2.40\cdot10^{10}$	$4.71\cdot10^{-6}$

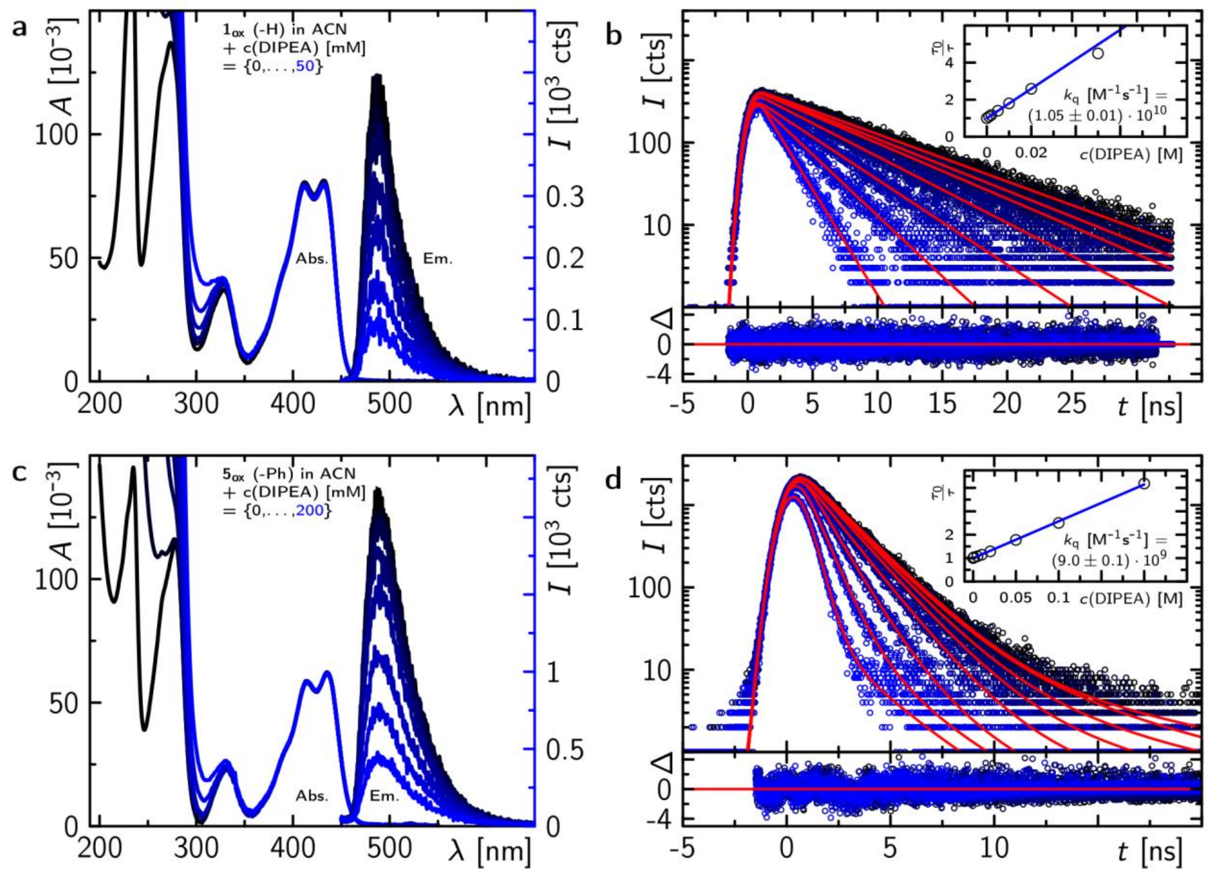

Supplementary Figure 6. **Excited singlet state quenching of $\mathbf{1_{ox}}$ and $\mathbf{5_{ox}}$.** Quenching of the excited singlet state of $\mathbf{1_{ox}}$ (**a**-**b**) and $\mathbf{5_{ox}}$ (**c**-**d**) by DIPEA in O_2 saturated ACN. **a** and **c**: Stationary absorption and emission spectra in dependence on the DIPEA concentration as indicated. **b** and **d**: Time-resolved emission after excitation at λ_{exc} = 443 nm and detection at λ_{det} = 490 nm (Δt_{IRF} < 1 ns) in dependence on the DIPEA concentration as indicated. The red lines represent exponential fits. The inset shows the Stern-Volmer analysis.

drops below 25% after 1 ns. Therefore, the emission decay data obtained from TCSPC with an instrument response function (IRF) of *ca.* 1 ns can sufficiently be analysed by the pseudo-first order approximation. The bi-molecular rate constants for the excited singlet state quenching of either $\mathbf{1_{ox}}$ or $\mathbf{5_{ox}}$ by DIPEA was determined by time-resolved emission spectroscopy. As can be seen in Supplementary Figure 6, the excited singlet of both photocatalysts is increasingly quenched in the presence of increasing DIPEA concentration. In both cases, a Stern-Volmer based bi-molecular quenching model describes the data well under low DIPEA concentrations (insets in panels **b** and **d** of Supplementary Figure 6). The resulting bi-molecular rate constants are $(1.05\pm0.01)\cdot10^{10}$ $M^{-1}\cdot s^{-1}$ and $(9.0\pm0.1)\cdot10^{9}$ $M^{-1}\cdot s^{-1}$ for $\mathbf{1_{ox}}$ and $\mathbf{5_{ox}}$, respectively. However, using higher DIPEA concentrations, deviations from the Stern-Volmer analysis are observed. For instance, in the case of $\mathbf{5_{ox}}$ as presented in Figure 3 panels **b** and **d** in the main text, a longer lifetime of 210 ps for 800 mM is observed although a lifetime of 138 ps is theoretically expected. This illustrates that the solvent properties are affected by the high DIPEA concentration (DIPEA as pure solvent is 5.8 mol/L).

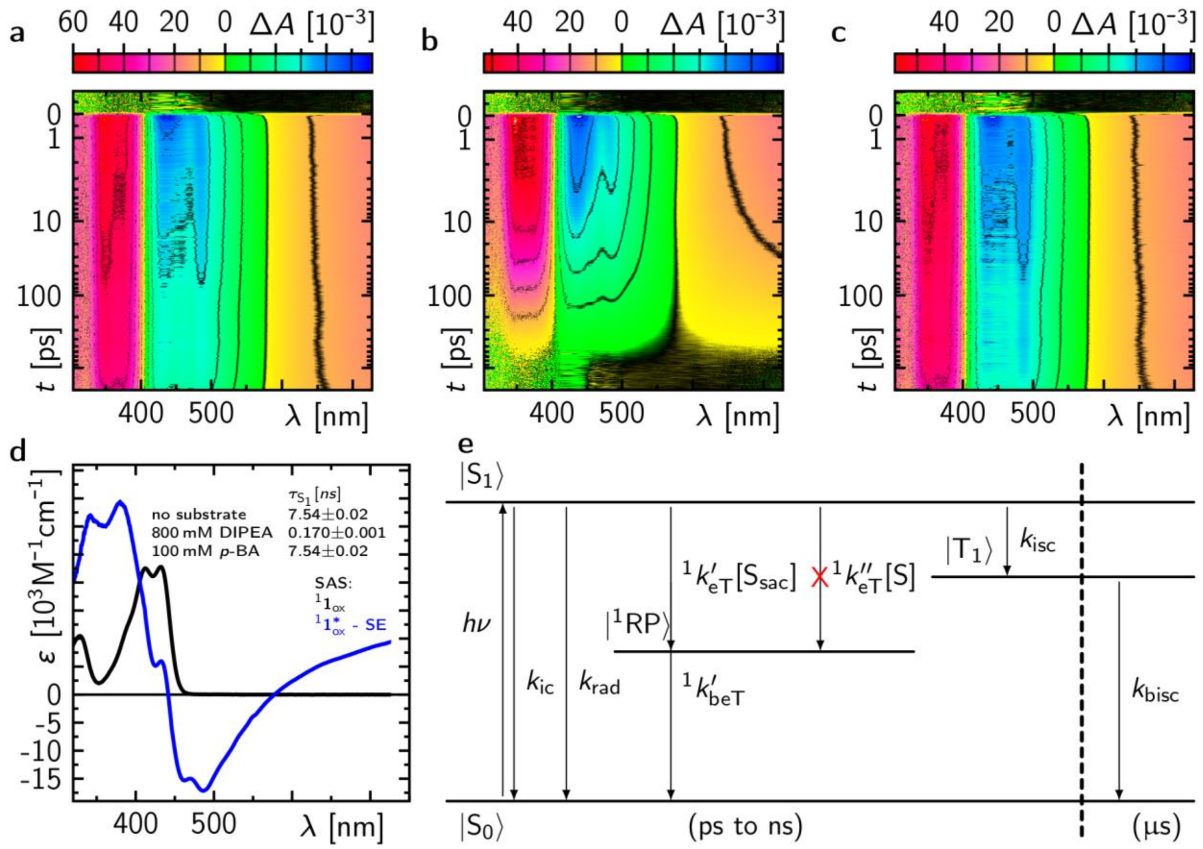

Supplementary Figure 7. **Excited singlet state dynamics of 1. a**-**c**: False colour representation of the time-resolved absorption spectra of **1** (1 mM) in non-degassed ACN in the absence (**a**), or presence of either 800 mM DIPEA (**b**), or 100 mM ***p*-BA** (**c**) excited at λ_{exc} = 450 nm. **d**: Species associated spectra (SAS) that contribute to the time-resolved absorption signals in panels **a**-**c** with excited singlet state lifetimes as indicated. **e**: Model used to describe the time-resolved absorption data that results in physically reasonable SAS. On this time window k_{isc} is negligible and since no further species spectrum is detectable the assumption ${}^{1}k'_{beT} \gg {}^{1}k'_{eT}[S_{sac}]$ is justified. Abbreviations: SE = stimulated emission, rad = radiative, ic = internal conversion, (b)eT = (back) electron transfer, (b)isc = (back) intersystem crossing, S_0 = singlet ground state, S_1 = excited singlet state, T_1 = triplet state, ^{1}RP = singlet born radical pair, S_{sac} = sacrificial electron donor, and S = substrate.

Comparison of these rates with the theoretical totally diffusion controlled bi-molecular rate constants estimated by the Smoluchowski theory (see Supplementary Table 4), shows a preference on encounter in both cases so that only 56% and 47% of all encounters lead to a reaction, respectively. As already discussed in the main article, the excited singlet state reaction of **1** with DIPEA results in a pure loss channel as also observed for $\mathbf{5_{ox}}$. In the presence of ***p*-BA**, also no reaction with the excited singlet state of **1** is observed. Supplementary Figure 7 shows the corresponding transient absorption data for compound **1** in ACN. In case of the stable fully reduced form $\mathbf{5_{red}}$ no reaction between the excited singlet state and the substrate ***p*-BA** is observed, even

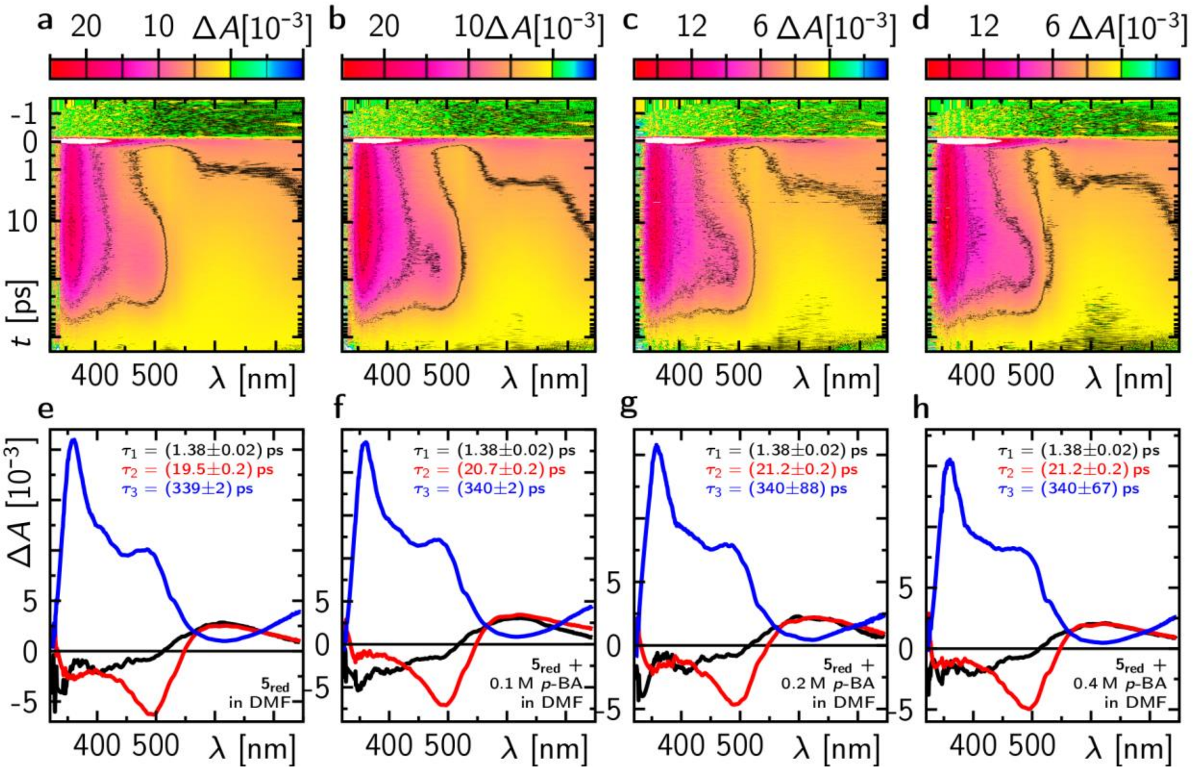

Supplementary Figure 8. **Excited singlet state dynamics of $\mathbf{5_{red}}$.** False colour representation of the time-resolved absorption spectra of $\mathbf{5_{red}}$ (500 μM) in O_2 saturated DMF exciting at λ_{exc} = 340 nm with (**b**-**d** and **f**-**h**) or without (**a** and **e**) *p*-bromoanisole (***p*-BA**); **b** and **f**: 100 mM; **c** and **g**: 200 mM; **d** and **h**: 400 mM. The lower panels **e**-**f** show the decay associated difference spectra (DADS) from a global tri-exponential fit.

at substrate concentrations up to 400 mM (Supplementary Figure 8). As can be seen, the excited state dynamics of $\mathbf{5_{red}}$ do not only show a single spectral component with some minor spectral shifts due to internal conversion and vibrational relaxation that might be attributed to the excited singlet state alone, but, interestingly, show more transient intermediates. However, since the presence of ***p*-BA** does not change these dynamics, we do not address this point any further in this work. A more detailed elucidation of these dynamics is currently in progress in our labs and will be presented in a separate article.

Supplementary Note 8

Testing the triplet state reactivity and the photochemical model. In the presence of the sacrificial electron donor DIPEA we observe, additionally to the unproductive excited singlet state reaction, a reaction with the triplet state of **5ox** as seen by a reduced triplet state lifetime and the semiquinone **5sq** (Figure 4 in the main text). Thus, in the kinetic model an additional rate constant enters. The global tri-exponential fit absolutely determines the three rate constants, which represent

$$\kappa_1 = k_{\mathrm{ic}} + k_{\mathrm{rad}} + k_{\mathrm{isc}} + {}^1k_{\mathrm{eT}} \tag{7}$$

$$\kappa_2 = k_{\mathrm{bisc}} + {}^3k_{\mathrm{eT}} \tag{8}$$

$$\kappa_3 = {}^3k_{\mathrm{beT}} \tag{9}$$

The model shown in Figure 4**g** in the main text leads to the following relationship between the SAS and the DADS:

$$S_{\mathrm{S}_1} = \frac{(D_1 + D_2 + D_3)}{c_0} + S_{\mathrm{S}_0} \tag{10}$$

$$S_{\mathrm{T}_1} = \frac{(\kappa_1 - \kappa_2)D_2 + (\kappa_1 - \kappa_3)D_3}{c_0 \Phi_{\mathrm{T}_1} \kappa_1} + S_{\mathrm{S}_0} \tag{11}$$

$$S_{\mathrm{D}_0} = \frac{(\kappa_2 - \kappa_3)(\kappa_1 - \kappa_3)D_3}{c_0 \Phi_{\mathrm{T}_1} \kappa_1 \Phi_{\mathrm{D}_0} \kappa_2} + S_{\mathrm{S}_0} \tag{12}$$

Here, c_0 is the contribution of the ground state spectrum and the Φ_i are the yields for the conversion from state i to the subsequent state $i + 1$. As can be seen, there are undetermined parameters c_0, Φ_{T_1}, and Φ_{D_0}. However, one can at least find upper or lower bounds by the requirement that the resulting SAS must be positive, and should not show any of the characteristic bands of the other species. In particular the negative peaks from the ground state bleach should disappear in the SAS. In case of Φ_{T_1} one knows from data sets with and without DIPEA that the yield of the unproductive excited singlet state reaction is given by $\Phi_{^1\mathrm{RP}} = 1 - k_{1,0}k_1^{-1}$, where $k_{1,0}$ is the excited singlet decay rate without DIPEA and k_1 is the excited singlet decay rate in the presence of DIPEA, respectively. Thus, for 50 mM DIPEA the loss yield *via* the excited singlet reaction is 29%. Consequently, the triplet yield reaches only 7.6% under these conditions. Analogously, $\Phi_{\mathrm{D}_0} = 1 - k_{2,0}k_2^{-1}$ is valid, where $k_{2,0}$ is the triplet decay rate without DIPEA and k_2 is the triplet decay rate in the presence of DIPEA, respectively. Thus, for 50 mM DIPEA the yield of **5sq** is 87%.

Supplementary Note 9

Probing the 5_{sq} *via* spectro-electrochemistry. Photocatalyst **5** in either its fully oxidised or its fully reduced form was electrochemically converted by application of either increasingly negative (Supplementary Figure 9) or positive (Supplementary Figure 10) potentials, respectively, and recording the UV/Vis absorption spectrum at the corresponding potential. Starting from $\mathbf{5}_{ox}$ and applying increasingly negative potentials, one observes initially on the expense of $\mathbf{5}_{ox}$ a build-up of a spectrum, that is similar to the pure species spectrum of $\mathbf{5}_{red}$ (black to red in Supplementary Figure 9**a**). However, a comparison with the pure species spectrum of $\mathbf{5}_{red}$ (Supplementary Figure 9 panels **b** and **d**) reveals significant differences. Furthermore, the first step does not show the formation of a spectrum known for semiquinones. Thus, it is tempting to speculate that in the initial conversion phase the main fraction of semiquinone molecules disproportionate faster than they are formed. Due to the lack of matching shape to the spectrum of $\mathbf{5}_{red}$, this conversion is accompanied by partial degradation. Interestingly, decreasing the potential further results in a clean build-up of the semiquinone form on the expense of $\mathbf{5}_{ox}$ as seen in clear isosbestic points (red to blue in Supplementary Figure 9**a**). Finally, going down to -2 V, $\mathbf{5}_{sq}$ converts to $\mathbf{5}_{red}$

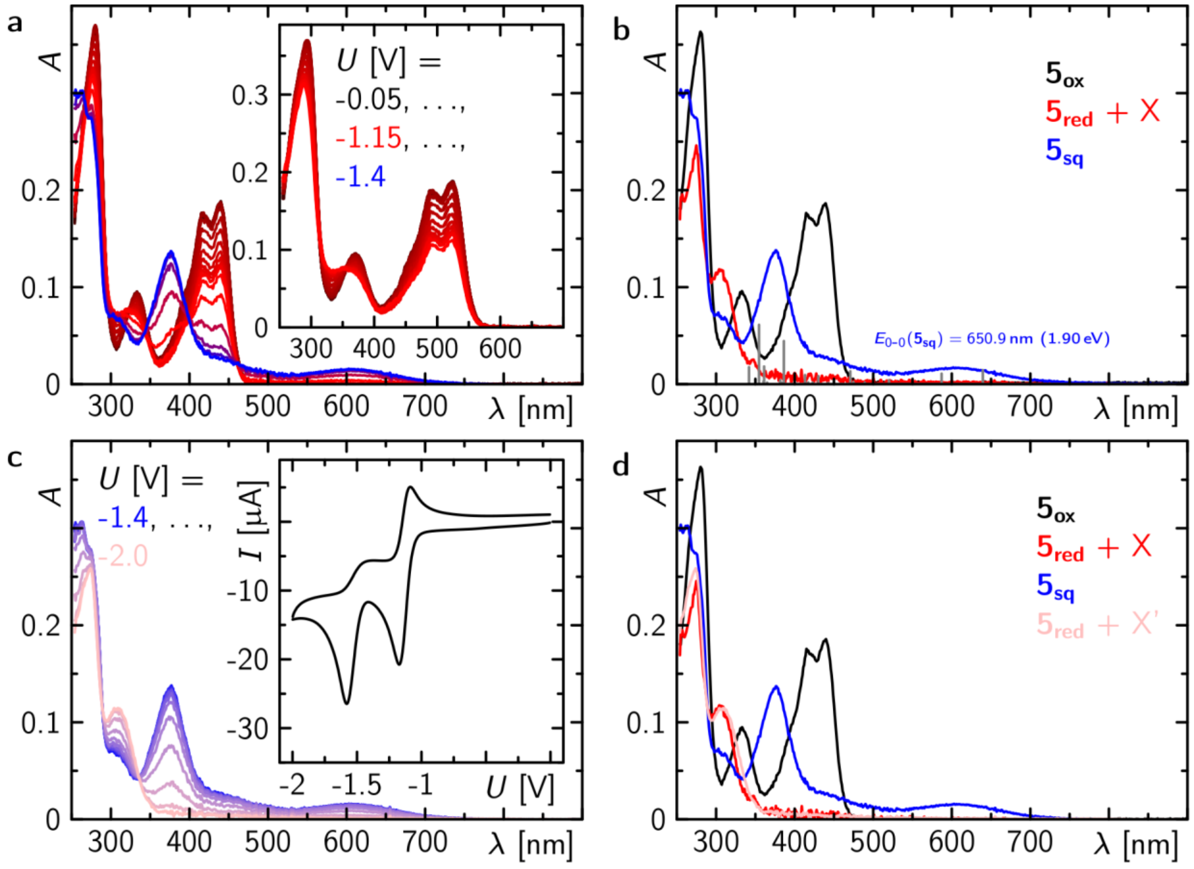

Supplementary Figure 9. **Electrochemical reduction of $\mathbf{5}_{ox}$ in degassed ACN. a** and **c**: Sequence of UV/Vis absorption spectra after stepwise application of increasingly negative voltage to the sample in 0.05 V increments as indicated. The inset in **c** shows the corresponding cyclic voltammogram. **b** and **d**: Species spectra contributing to corresponding data in **a** and **c**. The grey vertical lines in **b** correspond to the quantum chemically calculated stick spectrum of $\mathbf{5}_{sq}$ as described in the methods.

(blue to pink in Supplementary Figure 9**c**) by taking up a second electron. This step, again, occurs with clear isosbestic points indicating a clean conversion. Thus, the electrochemical reduction of $\mathbf{5_{ox}}$ in degassed ACN solution shows the transient formation of $\mathbf{5_{sq}}$, which indicates a higher stability compared to isoalloxazine semiquinones under similar conditions.[8]

In the next step, it was started from $\mathbf{5_{red}}$ and applied increasingly positive potentials. Initially, only small spectral changes in the UV region below 300 nm are observed (black to red in Supplementary Figure 10**a**). These might be attributed to small impurities in the sample that degrade. Subsequently, between 0.65 V and 1.25 V a clean conversion from $\mathbf{5_{red}}$ to $\mathbf{5_{ox}}$ with clear isosbestic points is observed (red to blue in Supplementary Figure 10**a**). Further increase of the potential results in complete degradation of the sample (blue to pink in Supplementary Figure 10**c**). Interestingly, no formation of $\mathbf{5_{sq}}$ is detected following this electrochemical route.

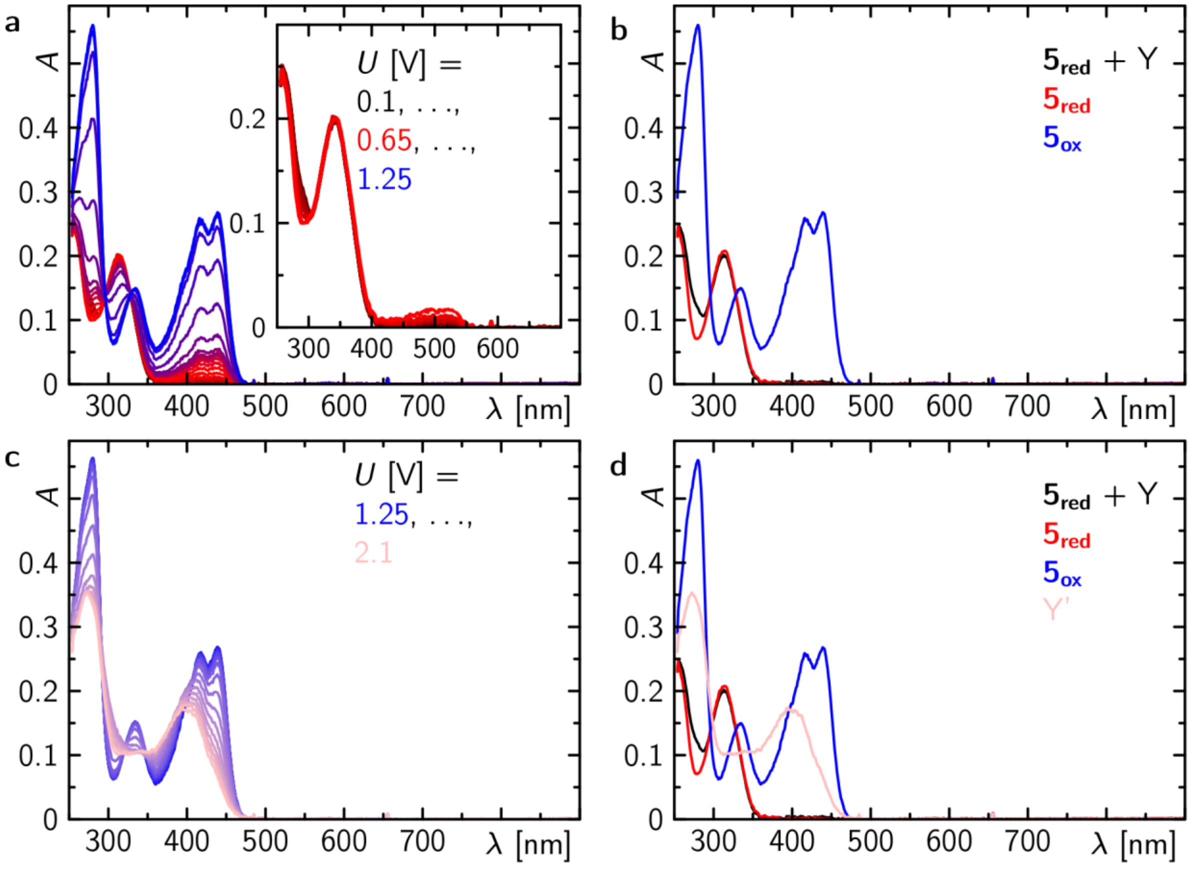

Supplementary Figure 10. **Electrochemical oxidation of $\mathbf{5_{red}}$ in ACN. a** and **c**: Sequence of UV/Vis absorption spectra after stepwise application of increasingly positive voltage to the sample in 0.05 V increments. **b** and **d**: Species spectra contributing to corresponding data in **a** and **c**.

Supplementary Note 10

Probing $\mathbf{5_{sq}}$ *via* electron paramagnetic resonance (EPR). $\mathbf{5_{ox}}$ in deaerated ACN in the presence of DIPEA was irradiated at 455 nm forming the semiquinone form, $\mathbf{5_{sq}}$, to a considerable amount which allowed the recording of its EPR spectrum (MiniScope MS400, Magnettech, 9.45 MHz, see Supplementary Figure 11). Additionally, the structure of $\mathbf{5_{sq}}$ was calculated quantum chemically with the programme package Orca[9,10] using DFT/B3LYP level of theory with EPR-II basis set in order to obtain the spin densities and the isotropic *g* value. A model including only hyperfine couplings between three nuclei, for instance representing ^{1}H-43, ^{1}H-44, and ^{1}N-14, and the electron was used to fit the data *via* the EasySpin toolbox[11] (Supplementary Figure 11).

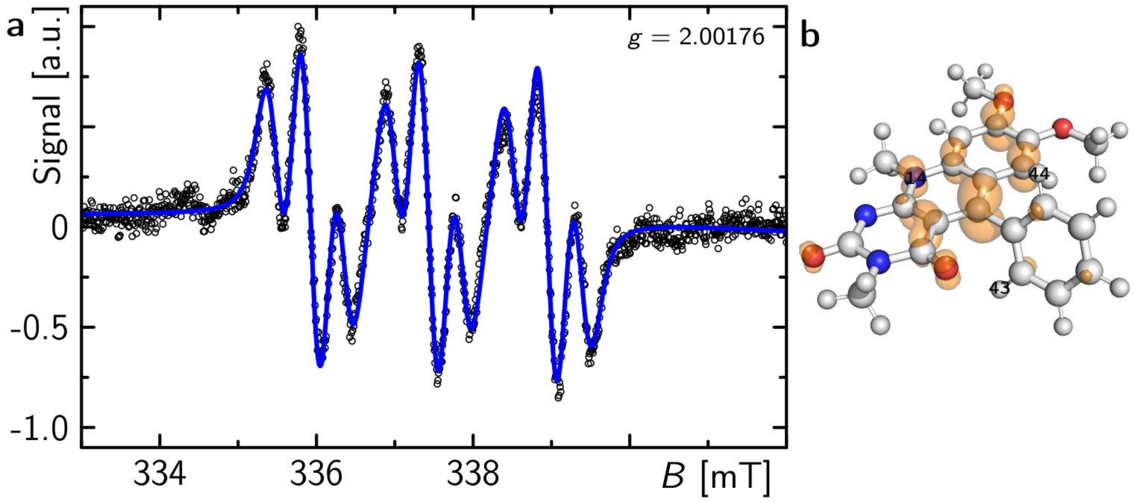

Supplementary Figure 11. **Electron paramagnetic resonance spectrum of $\mathbf{5_{sq}}$ in ACN.** Data plus fit (blue line) (**a**) and quantum chemically calculated structure (**b**). The nuclei used in the fitting model are ^{14}N-14 (Spin = 1, A_{iso} = 42.37 mHz), ^{1}H-43 (Spin = ½, A_{iso} = 11.35 mHz), and ^{1}H-44 (Spin = ½, A_{iso} = 12.38 mHz).

Supplementary Note 11

Potential reaction intermediates of *p*-BA. On the conversion from ***p*-BA** to ***p*-A** (anisole) two reaction intermediates should be theoretically observed. These are the radical anion, ***p*-BA**$^{\bullet-}$, and the neutral radical, ***p*-A**$^{\bullet}$. In our experimental data we observed at least one intermediate arising from the conversion of ***p*-BA**, which shows a prominent absorption band peaking at 330 nm that is shown in Figure 5**g** of the main text. In order to make a structural assignment to this transiently observed absorption spectrum, we calculated the absorption spectra of the substrate, ***p*-BA**, and the final product, ***p*-A**, as well as the potential two reaction intermediates ***p*-BA**$^{\bullet-}$ and ***p*-A**$^{\bullet}$ in the PCM(ACN) on the XMCQDPT-CASSCF level of theory using the aug-cc-pVDZ basis set. As evident from the calculations (Supplementary Figure 12**a**), the absorption spectrum of ***p*-BA**$^{\bullet-}$ is in very good agreement with the experimentally observed transient spectrum. This proofs electron transfer from the excited deazaflavin radical anion to the substrate ***p*-BA** forming the ***p*-BA**$^{\bullet-}$. Furthermore, calculation of the relaxed potential energy surface along the C-Br bond for ***p*-BA** and ***p*-BA**$^{\bullet-}$ (Supplementary Figure 12**b**) shows that on the one hand the bond length is considerably enlarged from 1.92 to 2.86 Å and on the other hand the dissociation energy is significantly reduced from 4.3 to 0.09 eV, respectively. Although the C-Br bond is clearly weakened in ***p*-BA**$^{\bullet-}$, its dissociation energy is still within the thermal energy range allowing its transient observation which is in excellent agreement with our experimental data (Figure 5**g** in the main text).

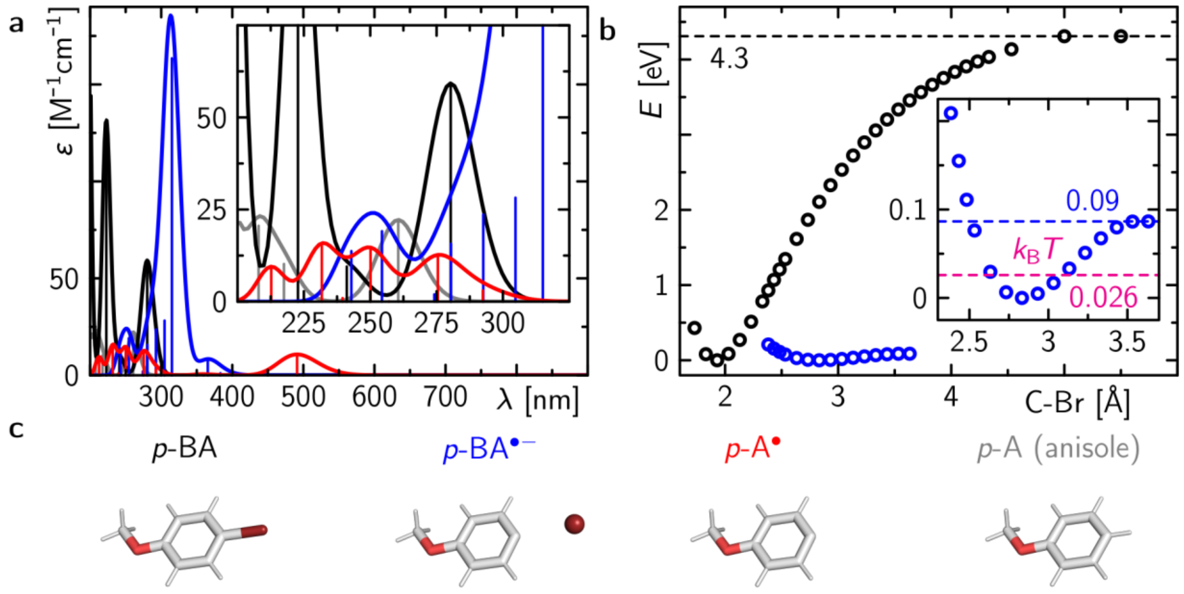

Supplementary Figure 12. **Quantum chemical investigation on potential intermediates.** Quantum chemical calculations of all species potentially involved in the conversion from ***p*-BA** to ***p*-A** (anisole). **a**: Electronic transitions (sticks) for the substrate, ***p*-BA** (black), and the final product, ***p*-A** (grey), as well as the two potential reaction intermediates ***p*-BA**$^{\bullet-}$ (blue) and ***p*-A**$^{\bullet}$ (red) convoluted with Gaussians (FWHM = 0.2 eV) calculated at the state-averaged XMCQDPT-CASSCF(12,12)-PCM(ACN)//aug-cc-pVDZ level of theory. **b**: Relaxed potential energy surface along the C-Br bond for ***p*-BA** and ***p*-BA**$^{\bullet-}$ using DFT(B3LYP) level of theory with the aug-cc-pVDZ basis set. The indicated thermal energy k_BT is plotted for 300 K. **c**: The corresponding optimised structures on the DFT(B3LYP) level of theory with aug-cc-pVDZ basis set.

Supplementary Figures

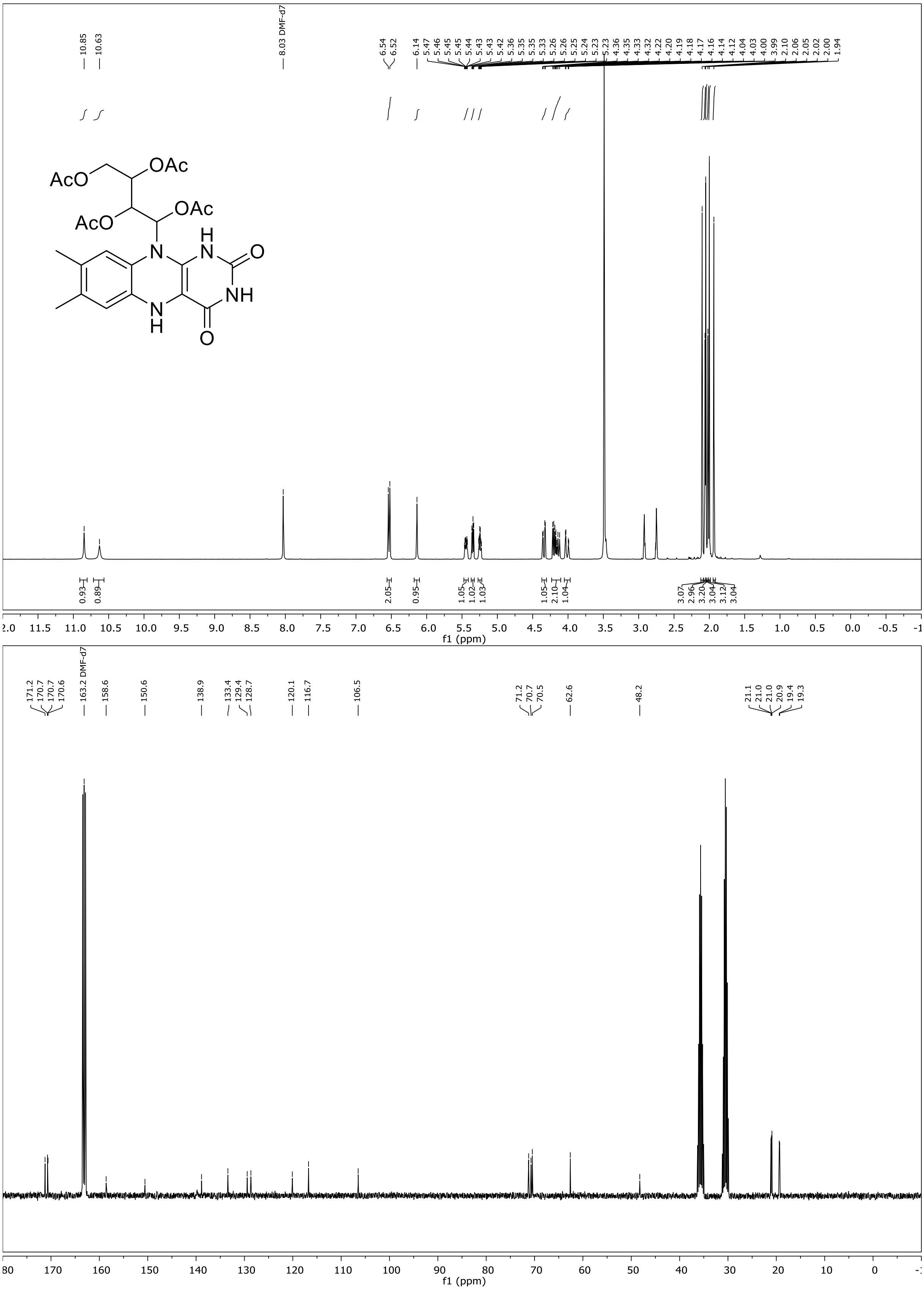

Supplementary Figure 13. NMR spectra of TARF-H_2.

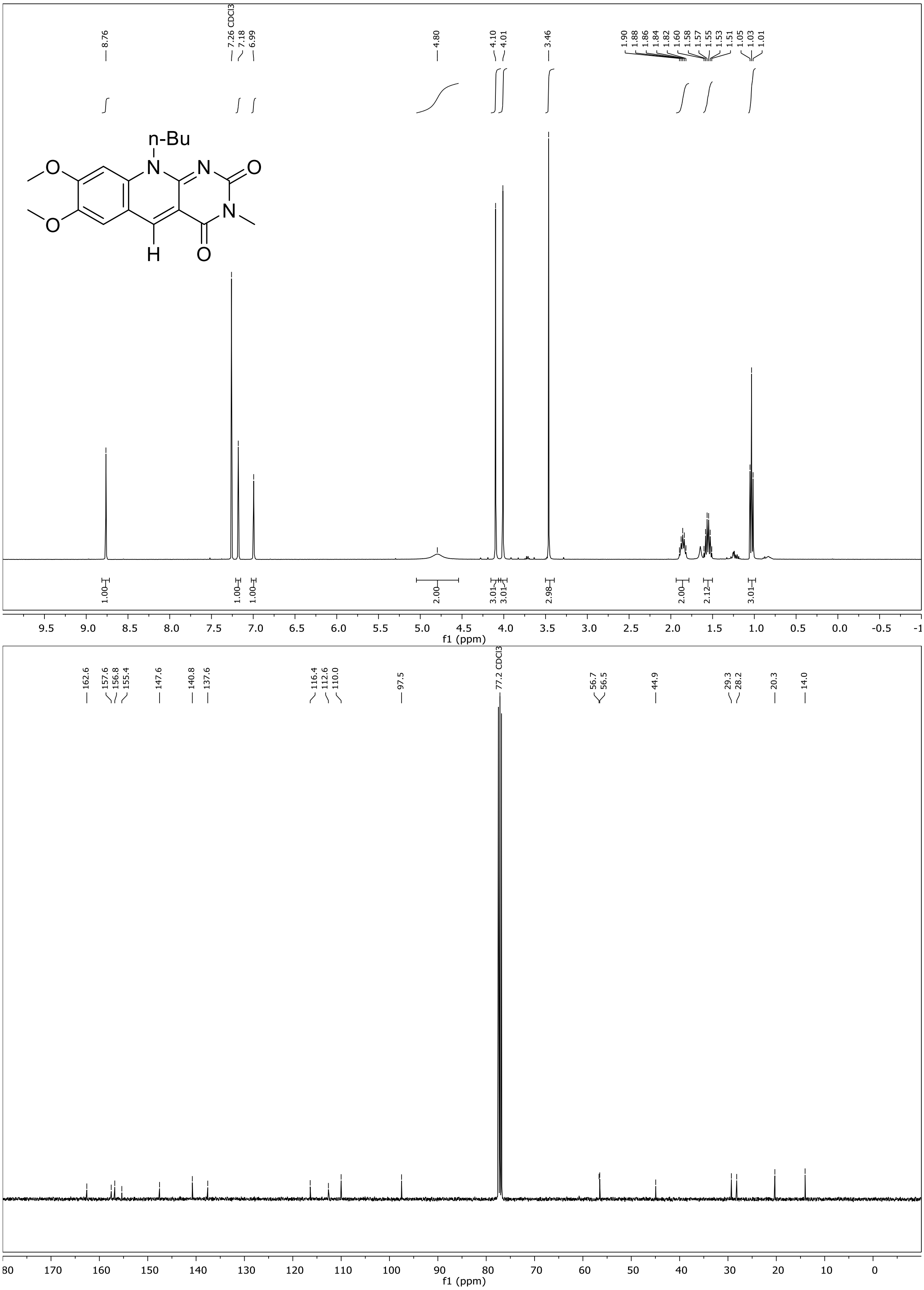

Supplementary Figure 14. NMR spectra of 10-Butyl-7,8-dimethoxy-3-methyl-5-deazaisoalloxazine (1).

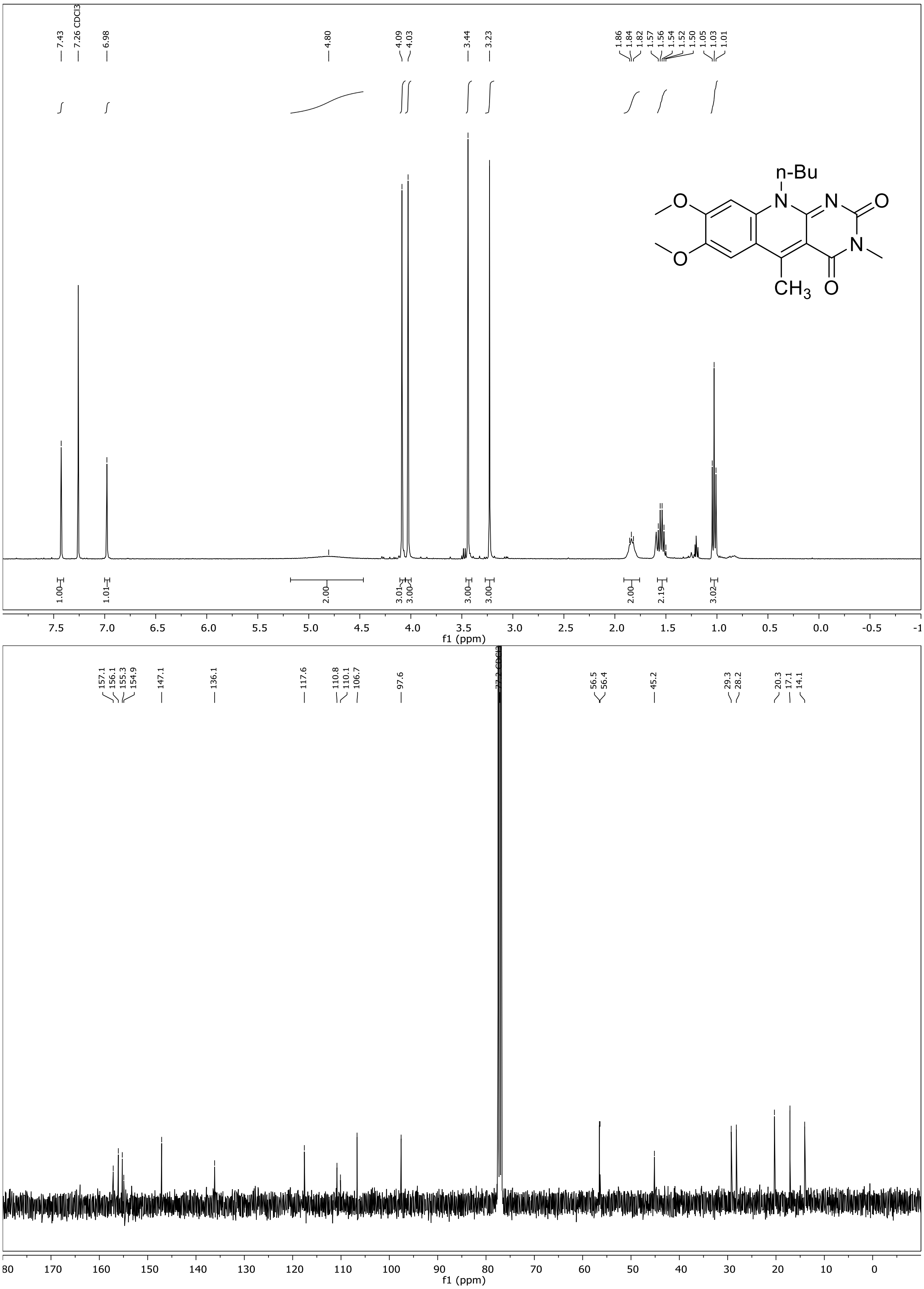

Supplementary Figure 15. NMR spectra of 10-Butyl-7,8-dimethoxy-3,5-dimethyl-5-deazaisoalloxazine (2).

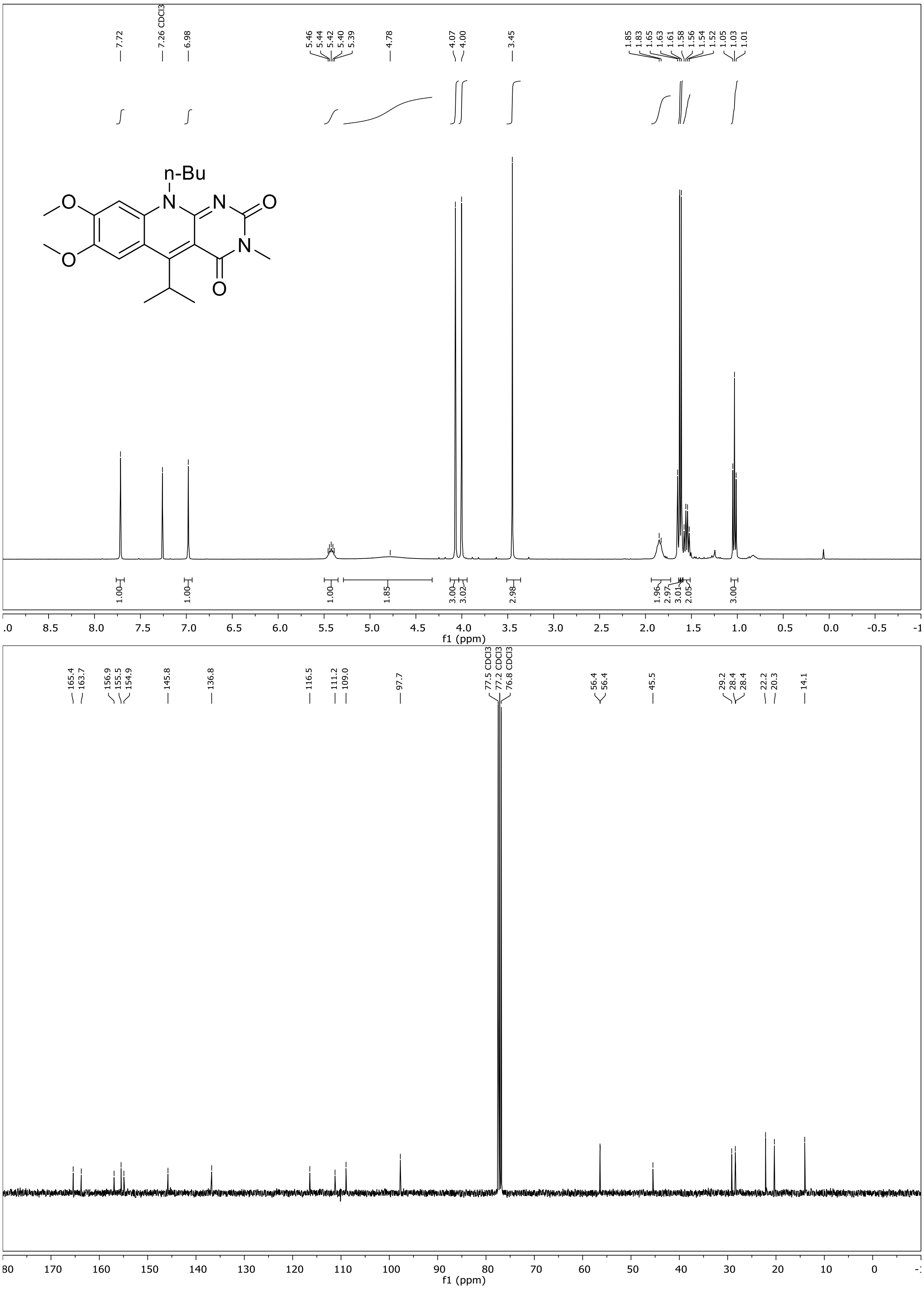

Supplementary Figure 16. NMR spectra of 10-Butyl-7,8-dimethoxy-5-isopropyl-3-methyl-5-deazaisoalloxazine (3).

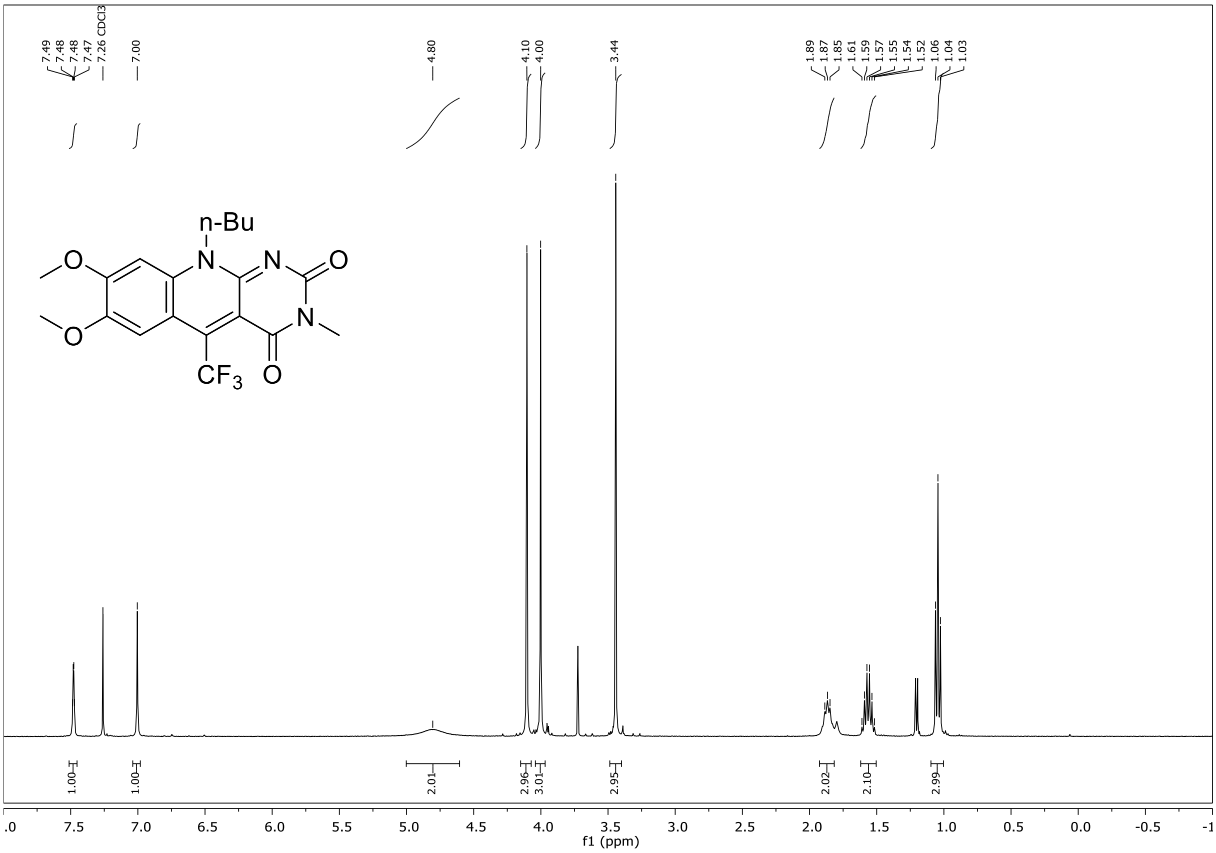
7.49
7.48
7.48
7.47
7.26 CDCl3
7.00
4.80
4.10
4.00
3.44
1.89
1.87
1.85
1.61
1.59
1.57
1.55
1.54
1.52
1.06
1.04
1.03
n-Bu
O
N
N
O
N
O
CF3
O
1.00
1.00
2.01
2.96
3.01
2.95
2.02
2.10
2.99
f1 (ppm)

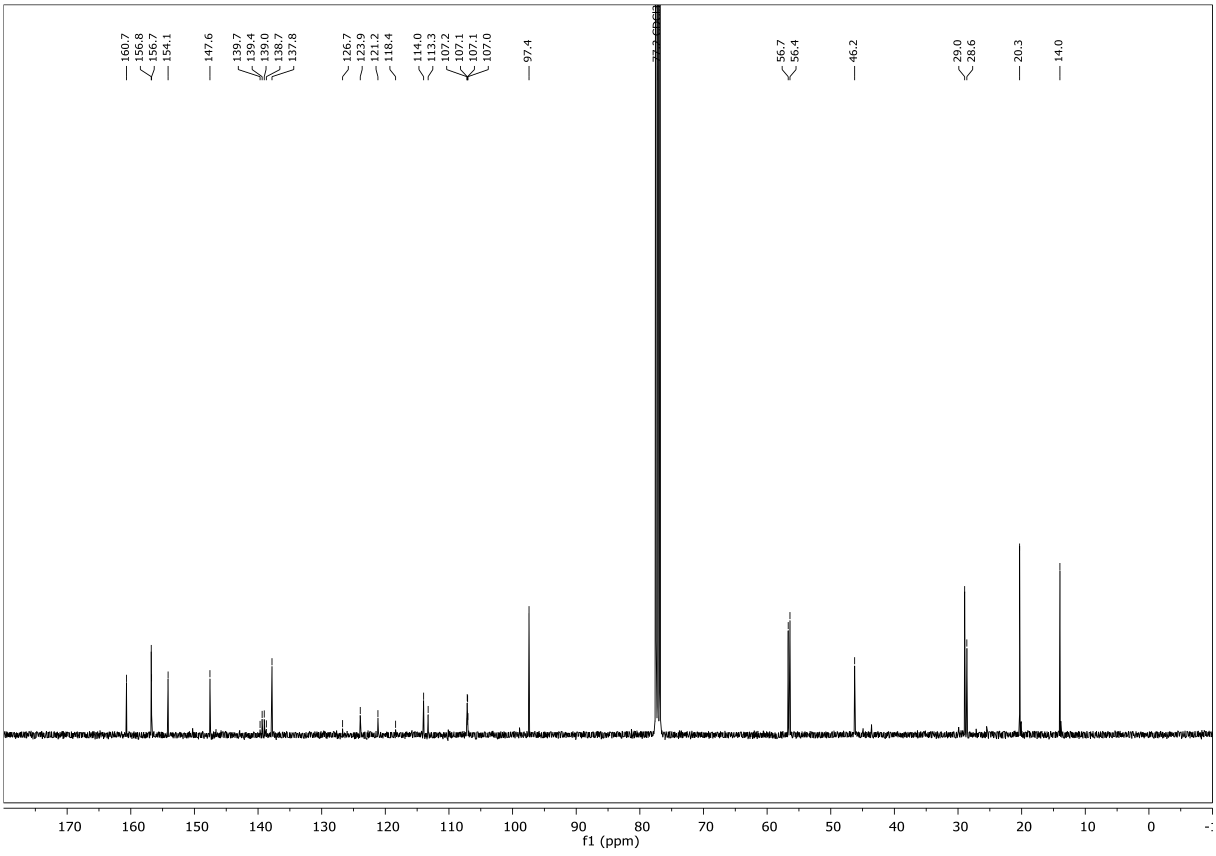
160.7
156.8
156.7
154.1
147.6
139.7
139.4
139.0
138.7
137.8
126.7
123.9
121.2
118.4
114.0
113.3
107.2
107.1
107.1
107.0
97.4
56.7
56.4
46.2
29.0
28.6
20.3
14.0
f1 (ppm)

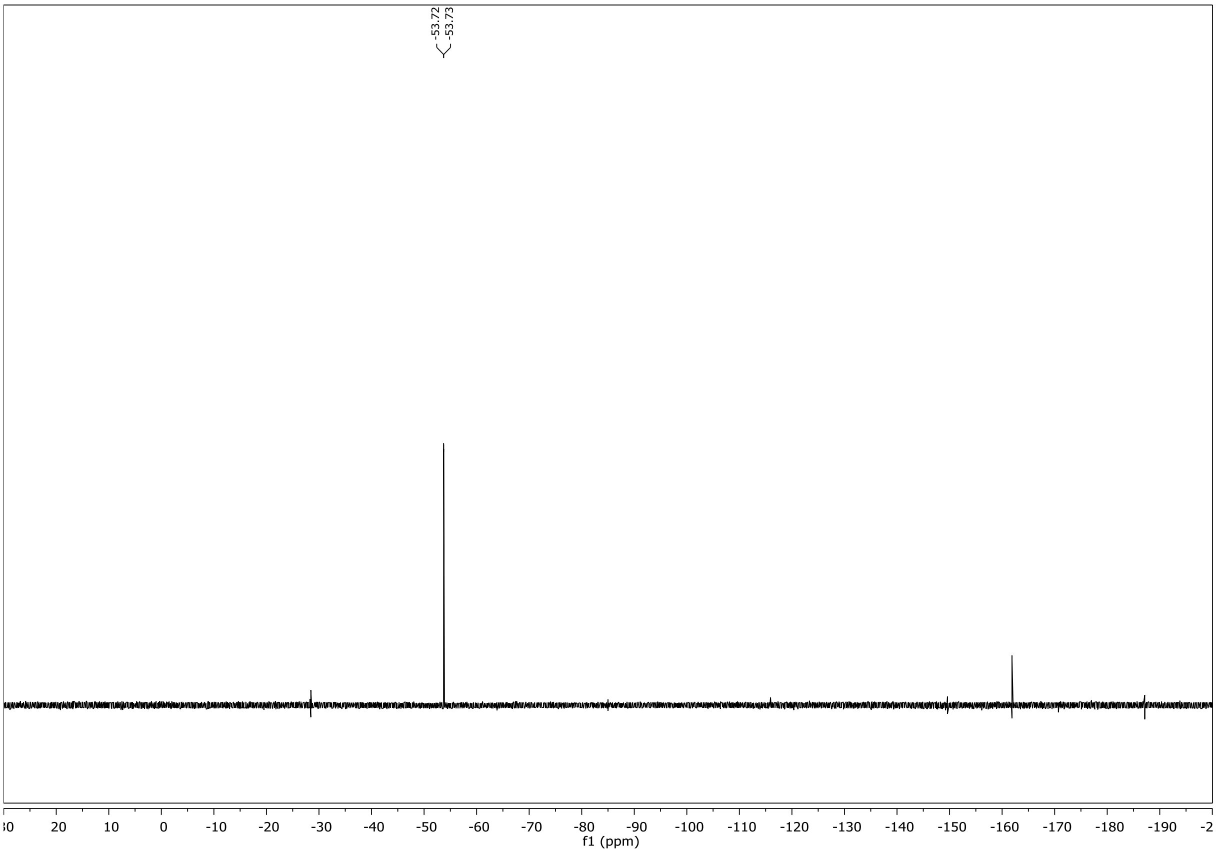

Supplementary Figure 17. NMR spectra of 10-Butyl-7,8-dimethoxy-3-methyl-5-trifluoromethyl-5-deazaisoalloxazine (4).

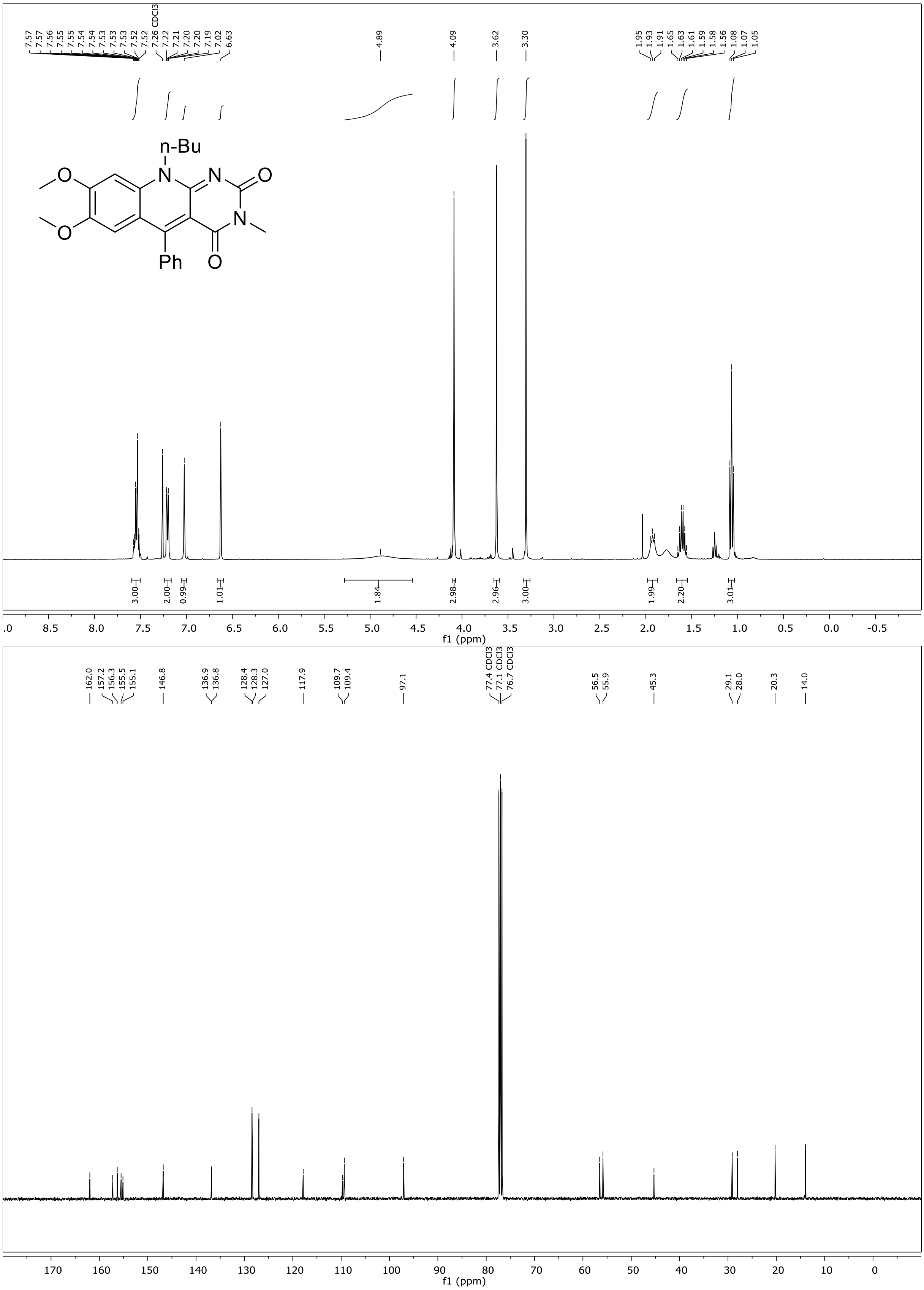

Supplementary Figure 18. NMR spectra of 10-butyl-7,8-dimethoxy-3-methyl-5-phenyl-5-deazaisoalloxazine (5_{ox}).

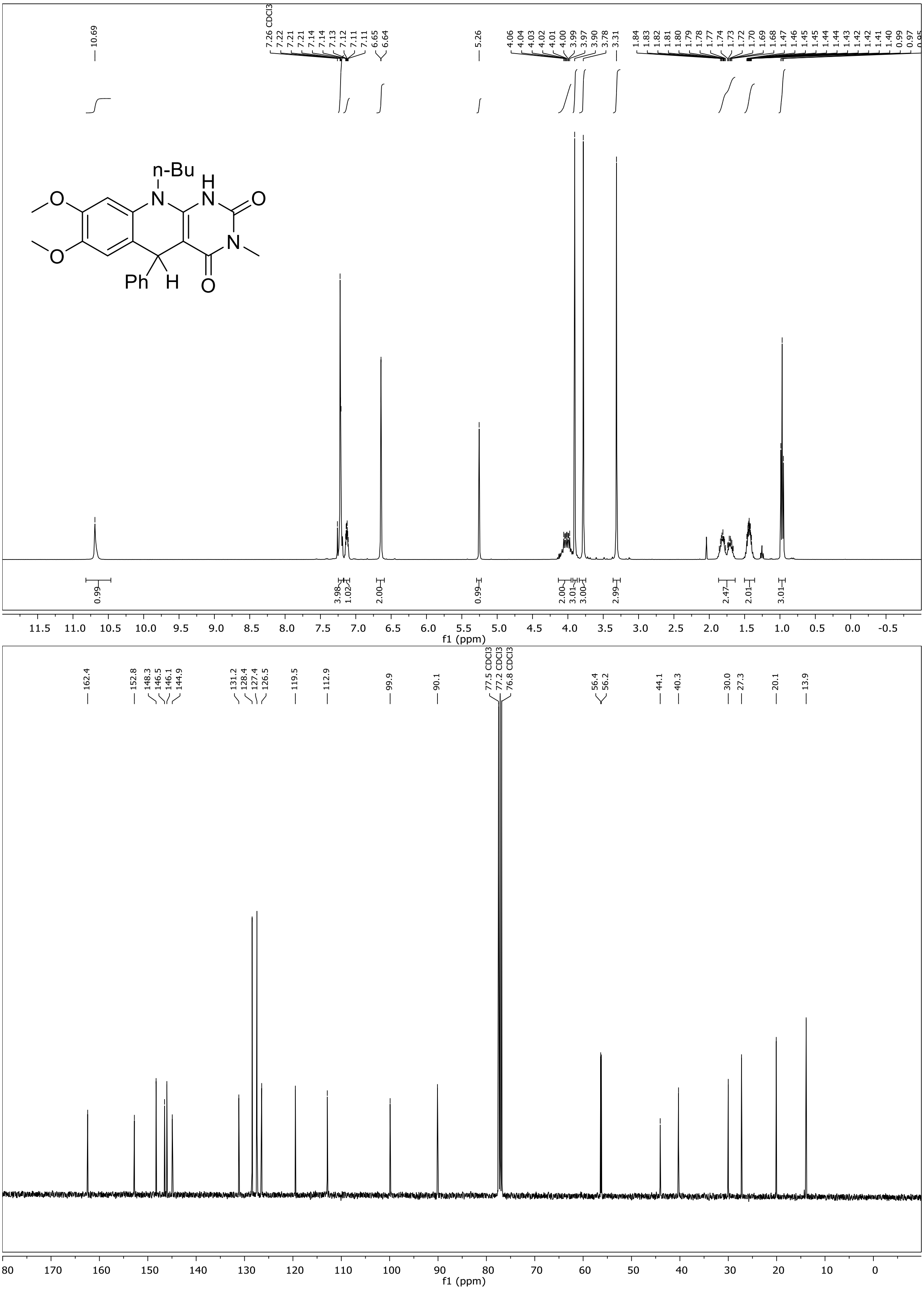

Supplementary Figure 19. NMR spectra of 10-butyl-7,8-dimethoxy-3-methyl-5-phenyl-5,10-dihydropyrimido[4,5-b]quinoline-2,4(1H,3H)-dione (5_{red}).

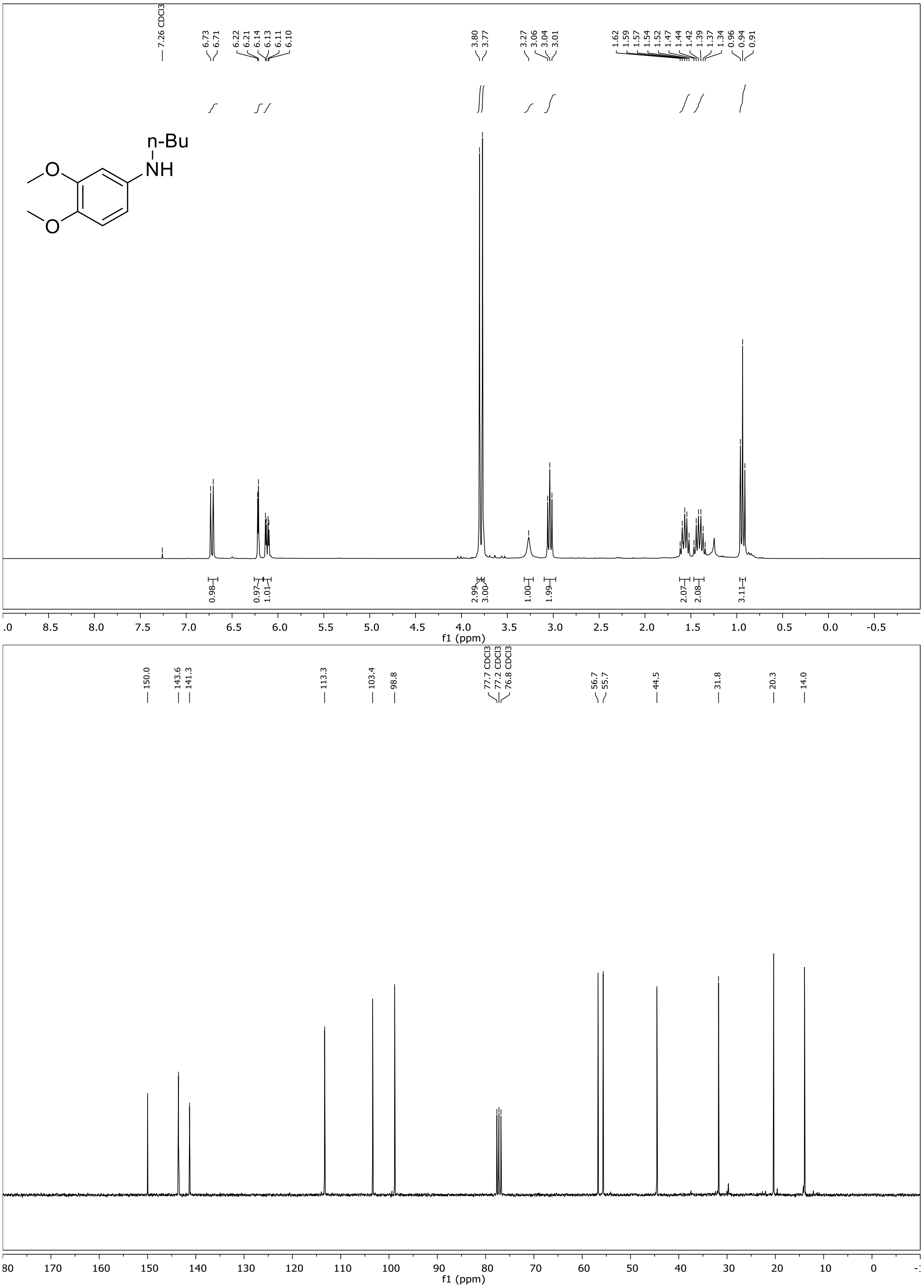

Supplementary Figure 20. NMR spectra of *N*-butyl-3,4-dimethoxyaniline (7).

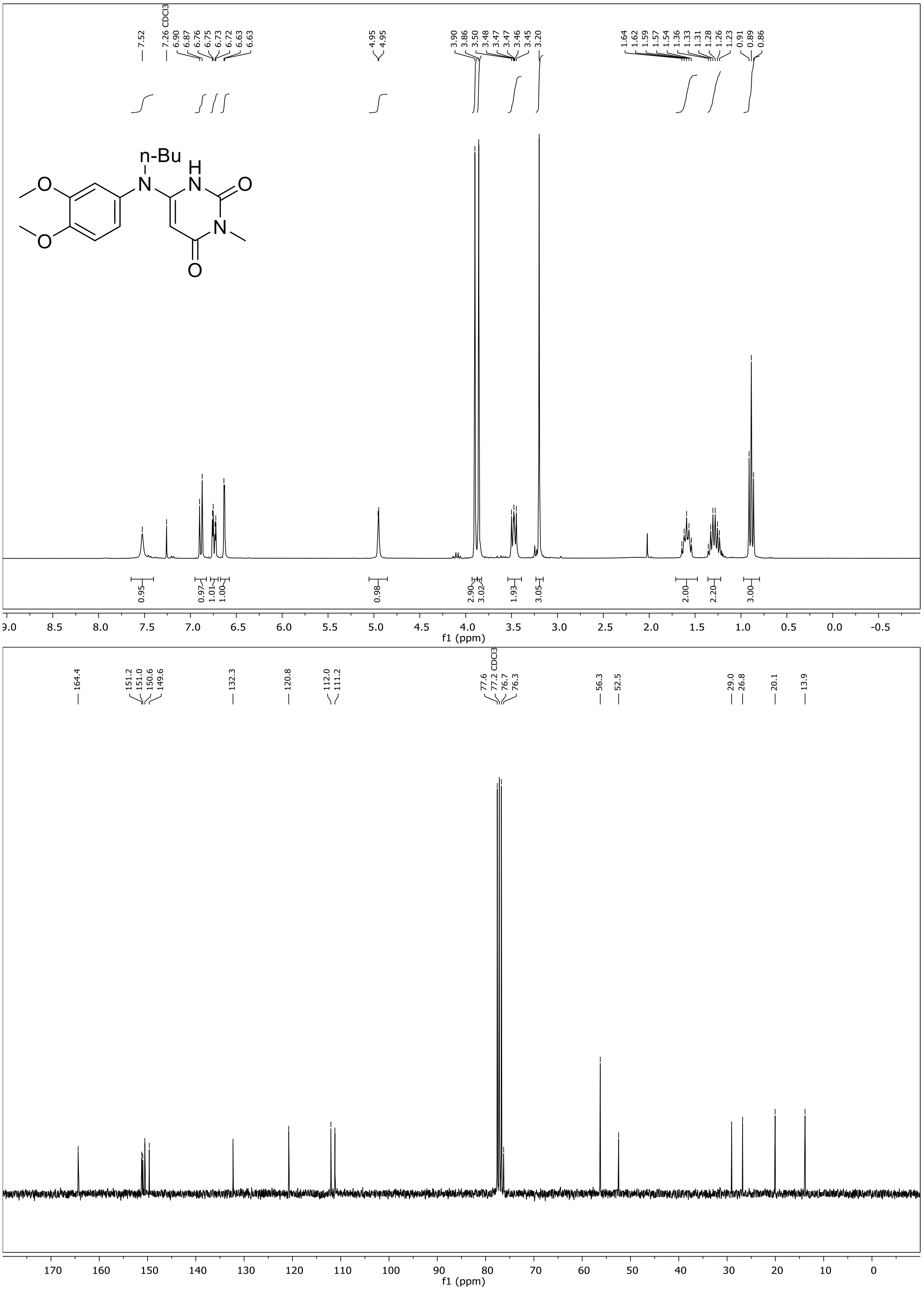

Supplementary Figure 21. NMR spectra of 6-(*N*-Butyl(3,4-dimethoxyphenyl)amino)-3-methyluracil (8).

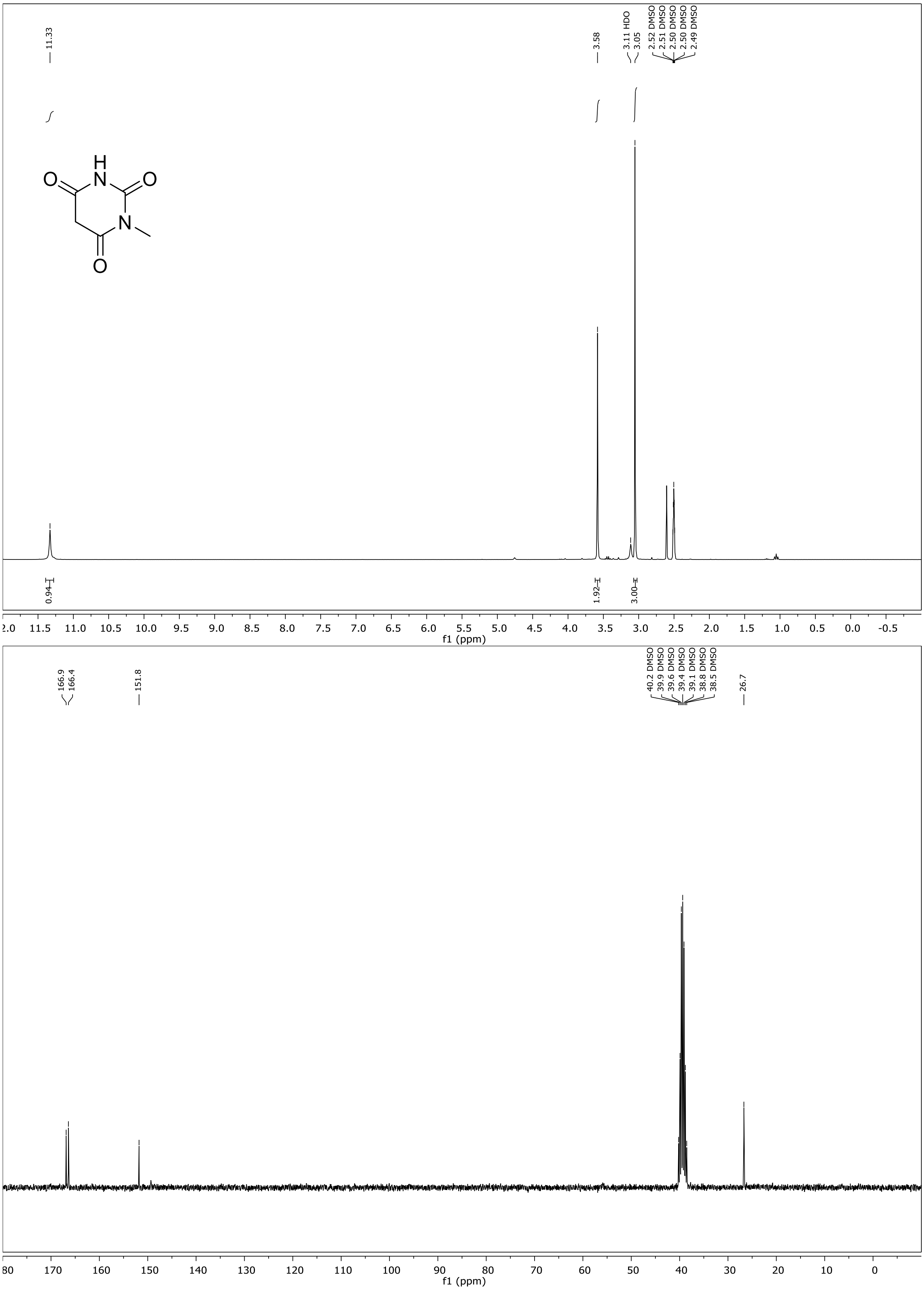

Supplementary Figure 22. NMR spectra of 1-methylpyrimidine-2,4,6(1H,3H,5H)-trione (9).

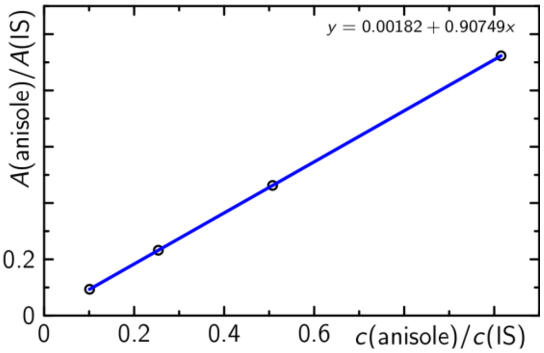

Supplementary Figure 23. **Gas chromatography calibration curve.** 4-methylanisole was used as internal standard (IS) for the quantitative analysis.

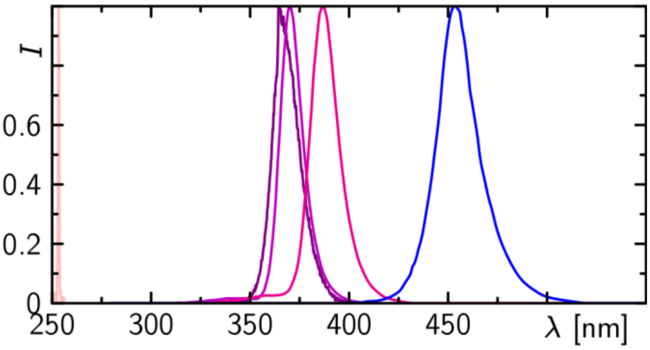

Supplementary Figure 24. **Emission profiles of all used LEDs for sample irradiation.** From left to right: λ_{max} = 254 nm, λ_{max} = 365 nm, λ_{max} = 370 nm, λ_{max} = 385 nm, and λ_{max} = 455 nm.

Supplementary References

1 Shi, F. *et al.* A facile and efficient synthesis of novel pyrimido[5,4-b][4,7]phenanthroline-9,11(7H,8H,10H,12H)-dione derivatives via microwave-assisted multicomponent reactions. *J. Heterocycl. Chem.* **46**, 563–566 (2009).

2 Abdel-Magid, A. F., Carson, K. G., Harris, B. D., Maryanoff, C. A. & Shah, R. D. Reductive Amination of Aldehydes and Ketones with Sodium Triacetoxyborohydride. Studies on Direct and Indirect Reductive Amination Procedures. *J. Org. Chem.* 61, 3849–3862 (1996).

3 Franco, C. & Olmsted, J.-I. Photochemical determination of the solubility of oxygen in various media. *Talanta* 37, 905–909 (1990).

4 Smoluchowski, M. V. Drei Vorträge über Diffusion, Brownsche Bewegung und Koagulation von Kolloidteilchen. *Zeitschrift für Physik* 17, 557–585 (1916).

5 Birks, J. B. Photophysics of Aromatic Molecules. (Wiley-Interscience, 1970).

6 Pavanï, R. & Ranghino, G. A method to compute the volume of a molecule. *Computers & Chemistry* 6, 133–135 (1982).

7 Dean, J. A. & Lange, N. A. Lange's Handbook of Chemistry. (McGraw-Hill, 1999).

8 Nöll, G. et al. Redox Properties of LOV Domains: Chemical versus Photochemical Reduction, and Influence on the Photocycle. *ChemBioChem* 8, 2256–2264 (2007).

9 Neese, F. The ORCA program system. *WIREs Comput. Mol. Sci.* 2, 73–78 (2012).

10 Neese, F. Software update: the ORCA program system, version 4.0. *WIREs Comput. Mol. Sci.* 8, e1327 (2018).

11 Stoll, S. & Schweiger, A. EasySpin, a comprehensive software package for spectral simulation and analysis in EPR. *J. Magn. Reson.* 178, 42–55 (2006).
