# Supplementary material for: Deazaflavin reductive photocatalysis involves excited semiquinone radicals
Source: Nat Commun. 2020 Jun 23;11:3174. doi: 10.1038/s41467-020-16909-y (PMC7311442; doi:10.1038/s41467-020-16909-y)
Supplement: Supplementary file 2 — Peer Review [file 41467_2020_16909_MOESM2_ESM.pdf]

Reviewer #1 (Remarks to the Author):

This work demonstrates reductive photocatalysis of deazaflavin compounds, and in particular the 5-phenyl derivative, allowing dehalogenation of p-BA substrates. This reductive flavin photochemistry is novel. First the overall photoreaction is shown to occur under continuous long-lasting illumination conditions. Subsequently, using various time-resolved spectroscopy approaches, it is convincingly demonstrated that this process involves two separate one-electron photoreactions: photoreduction of the starting oxidized compound to the unprotonated semiquinone form via triplet formation in the presence of a sacrificial electron donor, followed by photooxidation of this form in the presence of the substrate, generating the p-BA- form, precursor of dehalogenation. I think this is interesting work that merits to be published in Nat. Comm. The paper is written in a very concise way and the origin of assessments is not always straightforward to find; the internal referencing is sometimes sloppy. I have a number of points that need to be improved/clarified, partly concerning the latter issues.

1. In general, it would help if more and more specific (to sections or figures) reference to SI is given, for instance in lines 86, 88, 145.
2. In l 140, reference to Fig. 3 is given for spectroscopy on both compounds 1 and 5, but only compound 5 is shown.
3. The time constant of ET from 800 mM DIPEA in Fig. 3 (210ps) is somewhat longer than expected from the bimolecular rate constant apparently determined from Fig. S6 (should be referred to). Can the authors comment?
4. According to section S1.10 the pump-probe experiments of Fig. 3 were performed with 575 nm pump pulses. This is very surprising as the 5ox starting compound does not absorb at this wavelength.
5. Concentrations of the dFl compounds in all spectroscopic experiments should be given. The experiments of Fig. 3c have a much higher amplitude than those in Figs. 3a and 3b. What is the origin of this?
6. Is the negative signal at <450 nm in the t<0 delay zone of Fig. 3 due to long-lived bleaching?
7. L164, how was this bimolecular rate determined?
8. Fig. 4, I guess the time axis should be in us, not ps.
9. In Fig. 5, the formed species absorbing around 320 nm is assigned to p-BA-. In l 241-242 it is stated that this form should immediately dissociate. If this is true p-BA- should not be observed.
10. As two photoreactions are involved in this mechanism, one would expect the action spectrum to be some sort of combination of the 5ox and 5sq spectra, and/or optimal illumination to be with two distinct colors. Also, given the formation and decay time of the semiquinone intermediate, using intense pulses in the order of 100-ns rather than continuous illumination could be more efficient. Some discussion/perspective of this issue would be relevant.

Reviewer #2 (Remarks to the Author):

The manuscript by Graml et al. describes a detailed mechanistic study of a complex, multi-step photochemical reaction exploiting deazaflavins as reductive photocatalysts. In an impressive body of work, the mechanism is persuasively unravelled by a series of rigorous measurements using a combination of electrochemical, spectroelectrochemical, photoelectrochemical, and transient absorption spectroscopy methods. The study serves as an excellent demonstration of how an arsenal of cutting-edge physical methods can be used to unravelcomplex photochemical mechanisms, and the outcome is a useful addition to the synthetic photochemistry toolbox. It should therefore be of wide interest and suitable for Nature Communications. The manuscript is clearly written and illustrated (although the information content in some figures is perhaps too dense for easy legibility, e.g. Fig. 4f, Fig. 5f) and I recommend publication subject to consideration of the following points.

The authors quote several excited state lifetimes (e.g. Fig. 3d, Table S1) and bimolecular rate coefficients (e.g. page 5) without quoting uncertainties in the determinations. These uncertainties

should be added.

Fig. 3e and Fig. 4g would benefit from adding a "time" label to the x-axis to make clear that time increases to the right.

The time axis in Fig. 4a is incorrectly labelled as having ps units. I think the intended time unit is microseconds.

Fig. 4f labels the magenta trace as D1 but this should be D0 for the doublet ground state of the 5sq radical. Correcting this will avoid confusion with the 5sq* state encountered later.

There appears to be an element of fortune in the success of the photochemical pathway because the absorption spectrum of 5sq (Fig. 4f and Fig. 5) peaks in the same wavelength region used to photoexcite 5ox (around 385 nm). This is a necessary coincidence because both absorption steps are required to drive the reduction chemistry of aryl halides. Can the wavelength dependent results described in the Photodehalogenation section on page 4, and perhaps also the effects of use of DMF as a solvent or of a preference for basic conditions (either of which may shift the absorption spectra), be understood on the basis of optimal wavelengths for excitation of both the dFlox and dFlsq species? Does this requirement for overlapping absorptions place any limitations on the wider use of the authors' deazaflavin photocatalysis method for photocatalysts other than 5?

On page 10 the authors explain that the potential surface for the C-Br bond in p-BA·- is repulsive leading to immediate dissociation into Br- and an aryl radical. I agree, but then find their discussion in the text above about the p-BA·- spectrum to be misleading. The structure they isolate in quantum chemical calculations (Fig. 5) with a 2.86 A C-Br separation appears better described as a weakly associating complex of a p-anisole radical and Br-, and the carrier of the absorption band is then more appropriately the p-anisole radical, perhaps slightly perturbed by proximity to Br-. Did the authors calculate the spectrum of the p-anisole radical to see if it also agrees with the derived spectrum shown in Fig. 5g?

In the caption to Fig. 1, "no typical" should be "not typical".

Page 5, lines 147 – 148, the meaning of "indicating a preference for reactive encounters" is not clear. I think the authors intend to say that only about 50% of encounters are reactive because the reaction rate coefficient is approx. half that for the diffusion limit.

On page 6 of the supporting information there are a couple of missing citations in the section on transient absorption.

Reviewer #3 (Remarks to the Author):

This manuscript by Kutta, Cibulka, König, and coworkers showed a nice and interesting piece of work on reductive photocatalysis of deazaflavins. Various flavins and their analogues have been developed as redox- and photo-catalysts enabling diverse organic transformation, however, their synthetic applications have been generally limited to oxygenations and oxidations. In this work, the authors focused on deazaisoalloxazine ring systems with relatively lower reduction potentials in comparison with the commonly used isoalloxazines, and revealed their potentials for reductive photocatalysis by using the reductive dehalogenation of haloanisoles as an example. The investigation of the photocatalytic reaction mechanism is well executed, and the proposed mechanism (Figure 6) is supported by several experimental and theoretical proofs, which seem to be convincing. The findings would contribute to the advancement of novel photo-induced reductive transformations, therefore, this manuscript is worthy of publishing in Nature Communications. However, the following comments

should be taken into consideration before acceptance of this interesting manuscript.

1) The authors used the electrochemical method to elucidate the reactivity of 5sq towards the dehalogenation of p-BA, and the remaining spectrum with a peak at 330 nm, that was observed in the presence of p-BA and light (magenta line in Figure 5g), was identified as p-BA•−. Before this discussion, the authors should show that the dehalogenated anisole can also be yielded using the electrochemical method.

2) Lines 135-136, "Thus, a deprotonated form of the key intermediate is beneficial.": The authors should explain the reason why the base was not added in the absorption measurements (Figures 3-5). By the comparison with the results of the basic condition, it may possible to confirm what is the deprotonated key intermediate.

3) The present conPET system of the deazaflavins seems to be attractive, but the flavin-type catalysts are often unstable in the strongly basic reaction condition. Is there any information on the stability of the catalysts 1-5 in the reaction condition, e.g. in the condition of Table 2?

4) The abbreviated names of deazaflavins should be unified in the main-text and Supporting Information. For examples, dFl-Phox should be changed to 5ox.

We have revised the manuscript addressing all reviewers and editorial comments. The following changes were made in detail:

Response to the REVIEWER COMMENTS

Reviewer #1 (Remarks to the Author):

This work demonstrates reductive photocatalysis of deazaflavin compounds, and in particular the 5-phenyl derivative, allowing dehalogenation of p-BA substrates. This reductive flavin photochemistry is novel. First the overall photoreaction is shown to occur under continuous long-lasting illumination conditions. Subsequently, using various time-resolved spectroscopy approaches, it is convincingly demonstrated that this process involves two separate one-electron photoreactions: photoreduction of the starting oxidized compound to the unprotonated semiquinone form via triplet formation in the presence of a sacrificial electron donor, followed by photooxidation of this form in the presence of the substrate, generating the p-BA- form, precursor of dehalogenation. I think this is interesting work that merits to be published in Nat. Comm. The paper is written in a very concise way and the origin of assessments is not always straightforward to find; the internal referencing is sometimes sloppy. I have a number of points that need to be improved/clarified, partly concerning the latter issues.

1. In general, it would help if more and more specific (to sections or figures) reference to SI is given, for instance in lines 86, 88, 145.

**Reply:** We agree with the reviewer and appreciate her/his recommendation. Thus, we added specific section numbers in the corresponding references to the SI.

2. In l 140, reference to Fig. 3 is given for spectroscopy on both compounds 1 and 5, but only compound 5 is shown.

**Reply:** We thank the reviewer for indicating that we missed to present the corresponding transient absorption data for compound **1**. Now these data are presented in the SI and corresponding references to the Figure in the SI are given in the main text.

3. The time constant of ET from 800 mM DIPEA in Fig. 3 (210ps) is somewhat longer than expected from the bimolecular rate constant apparently determined from Fig. S6 (should be referred to). Can the authors comment?

**Reply:** The reviewer is entirely right. Considering the time-dependence of the bi-molecular reaction rate (as described in Section 3.3 in the SI) one would expect an even shorter lifetime under these conditions. However, using 800 mM of DIPEA (DIPEA as pure solvent is 5.8 mol/L) the solvent properties change significantly. The bi-molecular rate constant was determined under more diluted conditions via a Stern-Volmer analysis which gives a linear relationship up to 200 mM (see Figure S6d). For concentrations > 200 mM deviations from the Stern-Volmer analysis are observed as presented in Figure 3b and d illustrating that the solvent properties are affected by the high DIPEA concentration. Thus, we appreciate that the reviewer pointed out this discrepancy and added the following sentence in the main manuscript: [..] In case of the reaction between DIPEA and the excited singlet of the deazaflavins the bi-molecular reactions rates for **1** and $\mathbf{5_{ox}}$ under low substrate concentrations (< 200 mM DIPEA) are $(1.05\pm0.01)\cdot10^{10}$ $M^{-1}\cdot s^{-1}$ and $(9.0\pm0.1)\cdot10^{9}$ $M^{-1}\cdot s^{-1}$, respectively (see Stern-Volmer analysis in section 3.3 in the SI) [..] In SI we added the following sentences in section 3.3: [..] In both cases, a Stern-Volmer based bi-molecular quenching model describes the data well under low DIPEA concentrations (insets in panels **b** and **d** of **Figure S6**). The resulting bi-molecular rate constants are $(1.05\pm0.01)\cdot10^{10}$ $M^{-1}\cdot s^{-1}$ and $(9.0\pm0.1)\cdot10^{9}$ $M^{-1}\cdot s^{-1}$ for $\mathbf{1_{ox}}$ and $\mathbf{5_{ox}}$, respectively. However, using higher

DIPEA concentrations, deviations from the Stern-Volmer analysis are observed. For instance, in the case of **5**$_{ox}$ as presented in Figure 3b and d, a longer lifetime of 210 ps for 800 mM is observed although a lifetime of 138 ps is theoretically expected. This illustrates that the solvent properties are affected by the high DIPEA concentration (DIPEA as pure solvent is 5.8 mol/L). Comparison [..]

4. According to section S1.10 the pump-probe experiments of Fig. 3 were performed with 575 nm pump pulses. This is very surprising as the 5ox starting compound does not absorb at this wavelength.

**Reply:** The reviewer is completely right and we thank him/her for spotting this. We apologize for this. This has now been corrected to: [..] tuned to pump pulses centred at *ca.* 450 nm (*ca.* 100 fs, *ca.* 400 nJ at the sample position) for sample excitation [..]. Furthermore, we added the corresponding excitation wavelength used in the experiment where it was not already stated.

5. Concentrations of the dFl compounds in all spectroscopic experiments should be given. The experiments of Fig. 3c have a much higher amplitude than those in Figs. 3a and 3b. What is the origin of this?

**Reply:** We are grateful for spotting this inconsistency. Unfortunately, the wrong data set was loaded into panel **c**. In the past we also performed other experiments. Those data that were presented in Figure 3**c** in the initially submitted version were recorded in a different solvent (DMF) under different experimental conditions. The authors apologize for this mistake. The correct data set is now in place. All other data are correct as presented after double checking. Now all concentrations used in the transient absorption experiments are given in the corresponding Figure captions.

6. Is the negative signal at <450 nm in the t<0 delay zone of Fig. 3 due to long-lived bleaching?

**Reply:** The entire pre-t0 signal is flat and fluctuating around zero indicating clean transient absorption recording without any long-lived bleaching. The reviewer might have the impression that the signal at < 450 nm in the pre-t0 region is negative, because the noise level for probing at < 450 nm is simply higher than elsewhere. The false colour representation clearly fluctuates between yellow and green spots surrounded by the black value for zero.

7. L164, how was this bimolecular rate determined?

**Reply:** We are very grateful that the reviewer's question gives us the opportunity to correct and improve our manuscript. As we stated in section 3.4 of the SI, the yield for eT from DIPEA to the triplet can be determined from the corresponding decay rates to $\Phi_{\mathrm{eT}} = 1 - {k_0}/{k_\mathrm{q}}$, where $k_0$ is the total decay rate constants of the triplet in the absence and $k_\mathrm{q}$ in the presence of DIPEA. Finally, considering the concentration of DIPEA, the corresponding bi-molecular reaction rate with the triplet state is given to $k_{\mathrm{eT}} = \Phi_{\mathrm{eT}} k_q\,([\mathrm{DIPEA}])^{-1}$. We added the following sentence to the main manuscript: [..] The yield for eT can be determined from the corresponding decay rates to $\Phi_{\mathrm{eT}} = 1 - {k_0}/{k_\mathrm{q}} = 87\%$, where $k_0$ is the total decay rate constant of the triplet in the absence and $k_\mathrm{q}$ in the presence of DIPEA. Considering the concentration of DIPEA (50 mM), the corresponding bi-molecular reaction rate with the triplet state is given to $(8.7 \pm 0.1) \cdot 10^8$ $M^{-1}$ $s^{-1}$ ($k_{\mathrm{eT}} = \Phi_{\mathrm{eT}} k_q\,([\mathrm{DIPEA}])^{-1}$). [..]

8. Fig. 4, I guess the time axis should be in us, not ps.

**Reply:** The reviewer is absolutely right and we apologize for this. We thank him/her for spotting and changed it accordingly.

9. In Fig. 5, the formed species absorbing around 320 nm is assigned to p-BA-. In l 241-242 it is stated that this form should immediately dissociate. If this is true p-BA- should not be observed.

**Reply:** We are very grateful that the reviewer pointed out this putative contradiction, which gave us the opportunity to investigate this point in more detail and, thus, allowed us to improve on our manuscript. We performed further detailed quantum chemical calculations on all potential contributing species involved in the conversion from ***p*-BA** to ***p*-A** (anisole). The calculation of the absorption spectra clearly shows that only the spectrum of ***p*-BA**$^{\bullet-}$ agrees well with our experimental data. Furthermore, to address the dissociation issue, we calculated relaxed potential energy surfaces along the C-Br bonds for ***p*-BA** and ***p*-BA**$^{\bullet-}$. As expected, the dissociation energy in ***p*-BA** is 4.3 eV confirming its stability. In contrast, the singly reduced form, ***p*-BA**$^{\bullet-}$, shows a significantly reduced C-Br bond dissociation of only 0.09 eV. This is within the thermal energy range allowing its transient observation which is in excellent agreement with our experimental data. Thus, we added further computational details in section 1.11 of the SI and added a new section 5 in the SI including a new Figure S14: [..]

## 5. Potential reaction intermediates of *p*-BA

On the conversion from ***p*-BA** to ***p*-A** (anisole) two reaction intermediates should be theoretically observed in the spectra. These are the radical anion, ***p*-BA**$^{\bullet-}$, and the neutral radical, ***p*-A**$^{\bullet}$. In our experimental data we observed at least one intermediate arising from the conversion of ***p*-BA**, which shows a prominent absorption band peaking at 330 nm that is shown in **Figure 5 g** of the main text. In order to make a structural assignment to this transiently observed absorption spectrum, we calculated the absorption spectra of the substrate, ***p*-BA**, and the final product, ***p*-A**, as well as the potential two reaction intermediates ***p*-BA**$^{\bullet-}$ and ***p*-A**$^{\bullet}$ in the PCM(ACN) on the XMCQDPT-CASSCF level of theory using the aug-cc-pVDZ basis set. As evident from the calculations (**Figure S14 a**), the absorption spectrum of ***p*-BA**$^{\bullet-}$ is in very good agreement with the experimentally observed transient spectrum. This proofs electron transfer from the excited deazaflavin radical anion to the substrate ***p*-BA** forming the ***p*-BA**$^{\bullet-}$. Furthermore, calculation of the relaxed potential energy surface along the C-Br bond for ***p*-BA** and ***p*-BA**$^{\bullet-}$ (**Figure S14 b**) shows that on the one hand the bond length is considerably enlarged from 1.92 to 2.86 Å and on the other hand the dissociation energy is significantly reduced from 4.3 to 0.09 eV, respectively. Although the C-Br bond is clearly weakened in ***p*-BA**$^{\bullet-}$, its dissociation energy is still within the thermal energy range allowing its transient observation which is in excellent agreement with our experimental data (**Figure 5 g**).

[..]

Furthermore, we made additions in the main text as follows: [..] Considering … for ACN (see also section 5 in the SI). As seen in **Figures 5 g** and **S14 a**, the calculated spectrum matches the measured spectrum accurately, so that we assign this spectrum to ***p*-BA**$^{\bullet-}$. … Furthermore, the quantum chemical calculation revealed a significantly longer bonding length between the bromine and the adjacent carbon atom in ***p*-BA**$^{\bullet-}$ (2.86 Å) than in ***p*-BA** (1.92 Å) as well as a significantly reduced dissociation energy being close to thermal energy fluctuations (**Figure S14 b**), which explains well the observed final dissociation of the bromine during the dehalogenation which is triggered by a single electron reduction step as summarised in **Figure 5 h** (Further details are given in section 5 of the SI). … As a consequence, $\mathbf{5_{sq}}$ can be excited by a second photon ($E$*(**5**/$\mathbf{5_{sq}}$) = -3.3 V), which enables a consecutive photo-induced electron transfer (conPET)[58] from $\mathbf{5_{sq}}$* to the substrate, ***p*-BA**, regenerating $\mathbf{5_{ox}}$ in its ground state and closing the photocatalytic cycle. The dissociation energy for the bond between the bromine and the adjacent carbon in ***p*-BA**$^{\bullet-}$ is significantly reduced leading to thermally driven dissociation into $Br^-$ and the aryl radical. [..]

10. As two photoreactions are involved in this mechanism, one would expect the action spectrum to be some sort of combination of the 5ox and 5sq spectra, and/or optimal illumination to be with two distinct colors. Also, given the formation and decay time of the semiquinone intermediate, using

intense pulses in the order of 100-ns rather than continuous illumination could be more efficient. Some discussion/perspective of this issue would be relevant.

**Reply:** We thank the reviewer for giving us the opportunity to improve further on the manuscript by giving a discussion with future perspectives for improving that kind of conPET reaction. We added the following lines in the conclusion of the manuscript: [..] The action spectrum of the conPET reaction comprises the absorption of two different coloured photons by the two key photocatalytic forms of deazaflavins, where one is formed from the other having a distinct lifetime in the ns regime. Therefore, a concrete timed illumination of the photocatalytic system with short and intense pulses at the appropriate excitation wavelengths, which are temporally optimised to the lifetime of the key intermediates, should allow a further improvement on the efficiency. This aspect is of general importance for all conPET type reactions and should be addressed in future work. [..]

Reviewer #2 (Remarks to the Author):

The manuscript by Graml et al. describes a detailed mechanistic study of a complex, multi-step photochemical reaction exploiting deazaflavins as reductive photocatalysts. In an impressive body of work, the mechanism is persuasively unravelled by a series of rigorous measurements using a combination of electrochemical, spectroelectrochemical, photoelectrochemical, and transient absorption spectroscopy methods. The study serves as an excellent demonstration of how an arsenal of cutting-edge physical methods can be used to unravelcomplex photochemical mechanisms, and the outcome is a useful addition to the synthetic photochemistry toolbox. It should therefore be of wide interest and suitable for Nature Communications. The manuscript is clearly written and illustrated (although the information content in some figures is perhaps too dense for easy legibility, e.g. Fig. 4f, Fig. 5f) and I recommend publication subject to consideration of the following points.

The authors quote several excited state lifetimes (e.g. Fig. 3d, Table S1) and bimolecular rate coefficients (e.g. page 5) without quoting uncertainties in the determinations. These uncertainties should be added.

**Reply:** We thank the reviewer for pointing towards the missing uncertainties. We have now added all uncertainties in the text as well as figures where corresponding values are presented.

Fig. 3e and Fig. 4g would benefit from adding a “time” label to the x-axis to make clear that time increases to the right.

**Reply:** We thank the reviewer for his suggestion to add a ʹʹtimeʹʹ label to the x-axis. However, these panels represent energy state diagrams with rate constants for corresponding reaction steps, so that there is no actual time axis. There is only temporal information along the y-axis (which is itself an energy axis) going from top to bottom, where the corresponding rate constants include the temporal information. Thus, we decided to simply divide the corresponding reaction processes occurring on the two mentioned different time scales by the dashed line. We hope that the reviewer can accept this point of view.

The time axis in Fig. 4a is incorrectly labelled as having ps units. I think the intended time unit is microseconds.

**Reply:** The reviewer is absolutely right and we apologize for this. We thank him/her for spotting and changed it accordingly.

Fig. 4f labels the magenta trace as D1 but this should be D0 for the doublet ground state of the 5sq radical. Correcting this will avoid confusion with the 5sq* state encountered later.

**Reply:** The reviewer is absolutely right and we appreciate his/her support. We changed it accordingly.

There appears to be an element of fortune in the success of the photochemical pathway because the absorption spectrum of 5sq (Fig. 4f and Fig. 5) peaks in the same wavelength region used to photoexcite 5ox (around 385 nm). This is a necessary coincidence because both absorption steps are required to drive the reduction chemistry of aryl halides. Can the wavelength dependent results described in the Photodehalogenation section on page 4, and perhaps also the effects of use of DMF as a solvent or of a preference for basic conditions (either of which may shift the absorption spectra), be understood on the basis of optimal wavelengths for excitation of both the dFlox and dFlsq species? Does this requirement for overlapping absorptions place any limitations on the wider use of the authors' deazaflavin photocatalysis method for photocatalysts other than 5?

**Reply:** We appreciate the reviewer's questions on these important points. We performed further computational calculations and provided an additional section 4.4 in the SI including one additional Figure (Figure S 13) to address the wavelength, solvent, and basicity dependence on the total reaction yield. This now reads: [..]

**4.4 Theoretical absorption spectra of all protonation states of $\mathbf{5_{sq}}$**

The total photocatalytic conversion from ***p*-BA** to ***p*-A** (anisole) shows a dependence on the used solvent, the used excitation wavelength, and the basicity. Since the semiquinone form, $\mathbf{5_{sq}}$, of the photocatalyst is one key intermediate for the successful dehalogenation, we calculated quantum chemically absorption spectra of $\mathbf{5_{sq}}$ and its two potential protonation states, *i.e.* $\mathbf{5_{sq}^{C\text{-}H}}$ and $\mathbf{5_{sq}^{N\text{-}H}}$ in PCM for ACN and DMF (**Figure S13**). As evident, the theoretical and experimental absorption spectra of $\mathbf{5_{sq}}$ in ACN are in excellent agreement (see section 4.1 and **Figures S10** and **S13 a**). Considering the significant deviations of the absorption spectra of both protonated forms (**Figure S13 b** and **c**) from the experimentally recorded transient spectrum (**Figures 4** in the main text and **S10**) in the absence of base, we can already exclude the involvement of these species under neutral conditions and, thus, identify $\mathbf{5_{sq}}$ as the key intermediate of the conPET reaction. Furthermore, this is in accord with the observation that with excitation wavelength < 400 nm the total conversion under otherwise identical reaction conditions increased by a factor of 1.6, since $\mathbf{5_{sq}}$ has its highest absorption probability at around 360 nm. Moreover, the calculation shows that the most intense transitions around 365 nm of the $\mathbf{5_{sq}}$ in DMF shift apart resulting in an expected overall decreased extinction coefficient compared to the situation in ACN (**Figure S13 a**). Therefore, this might explain the observed decreased conversion yield by a factor of *ca.* 2 in DMF (**Table S4**).

In order to get more insights on the impact of the basicity on the reaction mechanism further studies are currently ongoing in our lab. At present, the working hypothesis explaining the observed enhanced yields under basic conditions are given by the following possible scenarios: 1) enhanced triplet yield; 2) longer triplet lifetime; 3) enhanced $\mathbf{dFl_{sq}}$ yield; or 4) longer lifetime of $\mathbf{dFl_{sq}}$. However, an enhancement of the final dehalogenation step after initiation by the second electron transfer can already be excluded since this part of the reaction is identical, thus, independent on the tested deazaflavin.

[..]

Furthermore, we modified the end of the ''Photodehalogenation'' section that now reads as follows: [..] Thus, a deprotonated form of one key intermediate seems to be beneficial (A more detailed discussion can be found in section 4.4 in the SI). [..].

Furthermore, in this particular system the spectrally broad excitation spectra of the used LEDs and the proximity of the absorption bands of the two key components of the conPET reaction surely were

beneficial. However, using two separate excitation sources, which potentially are short in time and intense at the appropriate excitation wavelength can be used for any kind of conPET system including the described deazaflavin based system. Furthermore, the two separate excitation sources might be temporally optimized to account for the particular lifetimes of the contributing species. We thank the reviewer for giving us the opportunity for this discussion which allows us to improve further on our manuscript. We added the following lines in the conclusion of the manuscript: [..] The action spectrum of the conPET reaction comprises the absorption of two different coloured photons by the two key photocatalytic forms of deazaflavins, where one is formed from the other having a distinct lifetime in the ns regime. Therefore, a concrete timed illumination of the photocatalytic system with short and intense pulses at the appropriate excitation wavelengths, which are temporally optimised to the lifetime of the key intermediates, should allow a further improvement on the efficiency. This aspect is of general importance for all conPET type reactions and should be addressed in future work. [..]

On page 10 the authors explain that the potential surface for the C-Br bond in p-BA·- is repulsive leading to immediate dissociation into Br- and an aryl radical. I agree, but then find their discussion in the text above about the p-BA·- spectrum to be misleading. The structure they isolate in quantum chemical calculations (Fig. 5) with a 2.86 A C-Br separation appears better described as a weakly associating complex of a p-anisole radical and Br-, and the carrier of the absorption band is then more appropriately the p-anisole radical, perhaps slightly perturbed by proximity to Br-. Did the authors calculate the spectrum of the p-anisole radical to see if it also agrees with the derived spectrum shown in Fig. 5g?

**Reply:** We are very grateful that the reviewer pointed out this putative contradiction, which gave us the opportunity to investigate this point in more detail and, thus, allowed us to improve on our manuscript. We performed further detailed quantum chemical calculations on all potential contributing species involved in the conversion from **_p_-BA** to **_p_-A** (anisole). The calculation of the absorption spectra clearly shows that only the spectrum of **_p_-BA$^{\bullet-}$** agrees well with our experimental data. Furthermore, to address the dissociation issue, we calculated relaxed potential energy surfaces along the C-Br bonds for **_p_-BA** and **_p_-BA$^{\bullet-}$**. As expected, the dissociation energy in **_p_-BA** is 4.3 eV confirming its stability. In contrast, the singly reduced form, **_p_-BA$^{\bullet-}$**, shows a significantly reduced C-Br bond dissociation of only 0.09 eV. This is within the thermal energy range allowing its transient observation which is in excellent agreement with our experimental data. Thus, we added further computational details in section 1.11 of the SI, added a new section 5 in the SI including a new Figure S14: [..]

**5. Potential reaction intermediates of _p_-BA**

On the conversion from **_p_-BA** to **_p_-A** (anisole) two reaction intermediates should be theoretically observed. These are the radical anion, **_p_-BA$^{\bullet-}$**, and the neutral radical, **_p_-A$^{\bullet}$**. In our experimental data we observed at least one intermediate arising from the conversion of **_p_-BA**, which shows a prominent absorption band peaking at 330 nm that is shown in **Figure 5 g** of the main text. In order to make a structural assignment to this transiently observed absorption spectrum, we calculated the absorption spectra of the substrate, **_p_-BA**, and the final product, **_p_-A**, as well as the potential two reaction intermediates **_p_-BA$^{\bullet-}$** and **_p_-A$^{\bullet}$** in the PCM(ACN) on the XMCQDPT-CASSCF level of theory using the aug-cc-pVDZ basis set. As evident from the calculations (**Figure S14 a**), the absorption spectrum of **_p_-BA$^{\bullet-}$** is in very good agreement with the experimentally observed transient spectrum. This proofs electron transfer from the excited deazaflavin radical anion to the substrate **_p_-BA** forming the **_p_-BA$^{\bullet-}$**. Furthermore, calculation of the relaxed potential energy surface along the C-Br bond for **_p_-BA** and **_p_-BA$^{\bullet-}$** (**Figure S14 b**) shows that on the one hand the bond length is considerably enlarged from 1.92 to 2.86 Å and on the other hand the dissociation energy is significantly reduced from 4.3 to 0.09 eV,

respectively. Although the C-Br bond is clearly weakened in ***p*-BA**$^{•−}$, its dissociation energy is still within the thermal energy range allowing its transient observation which is in excellent agreement with our experimental data (**Figure 5 g**).

[..]

Furthermore, we made additions in the main text as follows: [..] Considering … for ACN (see also section 5 in the SI). As seen in **Figures 5 g** and **S14 a**, the calculated spectrum matches the measured spectrum accurately, so that we assign this spectrum to ***p*-BA**$^{•−}$. … Furthermore, the quantum chemical calculation revealed a significantly longer bonding length between the bromine and the adjacent carbon atom in ***p*-BA**$^{•−}$ (2.86 Å) than in ***p*-BA** (1.92 Å) as well as a significantly reduced dissociation energy being close to thermal energy fluctuations (**Figure S14 b**), which explains well the observed final dissociation of the bromine during the dehalogenation which is triggered by a single electron reduction step as summarised in **Figure 5 h** (Further details are given in section 5 of the SI). … As a consequence, $\mathbf{5_{sq}}$ can be excited by a second photon ($E$*($\mathbf{5}$/$\mathbf{5_{sq}}$) = -3.3 V), which enables a consecutive photo-induced electron transfer (conPET)[58] from $\mathbf{5_{sq}}$***** to the substrate, ***p*-BA**, regenerating $\mathbf{5_{ox}}$ in its ground state and closing the photocatalytic cycle. The dissociation energy for the bond between the bromine and the adjacent carbon in ***p*-BA**$^{•−}$ is significantly reduced leading to thermally driven dissociation into $Br^{−}$ and the aryl radical. [..]

In the caption to Fig. 1, “no typical” should be “not typical”.

**Reply:** The authors agree that this wording might not be ideal. We decided to change it to ‘’untypical’’.

Page 5, lines 147 – 148, the meaning of “indicating a preference for reactive encounters” is not clear. I think the authors intend to say that only about 50% of encounters are reactive because the reaction rate coefficient is approx. half that for the diffusion limit.

**Reply:** The reviewer is right in that we intend to say that only about 50% of encounters are reactive. By stating that this indicates a preference for reactive encounters we provide one explanation for the deviation from the theoretically expected fully diffusion-controlled reaction rate limit on a structural level. In other words, for a reaction to occur the two reacting species need to encounter from specific sites which provide the necessary geometry, interactions, and, thus, probability. However, we acknowledge that this statement was probably not clear enough. Thus, we added the following statement: [..] These values are smaller by 56% and 47%, respectively, compared to the theoretical diffusion limited bi-molecular reaction limit (see section 3.3 in the SI for a detailed discussion) which might indicate a geometrical preference for reactive encounters allowing for necessary interactions, and thus, enabling a high probability for the reaction to proceed.[..]

On page 6 of the supporting information there are a couple of missing citations in the section on transient absorption.

**Reply:** We thank the reviewer for spotting this. This is a compatibility issue between different word versions used for editing this manuscript, which we unfortunately have overlooked. We apologize for this. In the revised version we carefully double checked the main manuscript and SI for any formatting issues.

Reviewer #3 (Remarks to the Author):

This manuscript by Kutta, Cibulka, König, and coworkers showed a nice and interesting piece of work on reductive photocatalysis of deazaflavins. Various flavins and their analogues have been developed as redox- and photo-catalysts enabling diverse organic transformation, however, their synthetic applications have been generally limited to oxygenations and oxidations. In this work, the authors focused on deazaisoalloxazine ring systems with relatively lower reduction potentials in comparison with the commonly used isoalloxazines, and revealed their potentials for reductive photocatalysis by using the reductive dehalogenation of haloanisoles as an example. The investigation of the photocatalytic reaction mechanism is well executed, and the proposed mechanism (Figure 6) is supported by several experimental and theoretical proofs, which seem to be convincing. The findings would contribute to the advancement of novel photo-induced reductive transformations, therefore, this manuscript is worthy of publishing in Nature Communications. However, the following comments should be taken into consideration before acceptance of this interesting manuscript.

1) The authors used the electrochemical method to elucidate the reactivity of 5sq towards the dehalogenation of p-BA, and the remaining spectrum with a peak at 330 nm, that was observed in the presence of p-BA and light (magenta line in Figure 5g), was identified as p-BA•–. Before this discussion, the authors should show that the dehalogenated anisole can also be yielded using the electrochemical method.

**Reply:** We thank the reviewer for his/her suggestion to record the intermediate form ***p*-BA**$^{\bullet-}$ electrochemically. We tried to perform this experiment, but failed due to experimental issues. Unfortunately, under our experimental conditions we observed gas evolution in the cell starting at *ca.* -2 V for ACN and -2.5 V for DMF. This made the recording of any useable absorption spectrum impossible. Since the first reduction potential of ***p*-BA** is at -2.75 V we could not reach to the point where the reaction would have started. We are aware that the electrochemical [1]window of thoroughly dried ACN and DMF should be broad enough to cover the potential range. However, it seems that the ottle cell setup with a thin sample film and high surface of the electrode causes remaining impurities of water to react at its electrode surface.

For further strengthening that we identified *p*-BA$^{\bullet-}$as an intermediate, we performed further detailed quantum chemical calculations on all potential contributing species involved in the conversion from ***p*-BA** to ***p*-A** (anisole). The calculation of the absorption spectra clearly shows that only the spectrum of ***p*-BA**$^{\bullet-}$ agrees well with our experimental data. Furthermore, to address the dissociation issue, we calculated relaxed potential energy surfaces along the C-Br bonds for ***p*-BA** and ***p*-BA**$^{\bullet-}$. As expected, the dissociation energy in ***p*-BA** is 4.3 eV confirming its stability. In contrast, the singly reduced form, ***p*-BA**$^{\bullet-}$, shows a significantly reduced C-Br bond dissociation of only 0.09 eV. This is within the thermal energy range allowing its transient observation which is in excellent agreement with our experimental data. Thus, we added further computational details in section 1.11 of the SI, added a new section 5 in the SI including a new Figure S14: [..]

**5. Potential reaction intermediates of *p*-BA**

On the conversion from ***p*-BA** to ***p*-A** (anisole) two reaction intermediates should be theoretically observed. These are the radical anion, ***p*-BA**$^{\bullet-}$, and the neutral radical, ***p*-A**$^{\bullet}$. In our experimental data we observed at least one intermediate arising from the conversion of ***p*-BA**, which shows a prominent absorption band peaking at 330 nm that is shown in **Figure 5 g** of the main text. In order to make a structural assignment to this transiently observed absorption spectrum, we calculated the absorption spectra of the substrate, ***p*-BA**, and the final product, ***p*-A**, as well as the potential two reaction

intermediates ***p*-BA**$^{\bullet-}$ and ***p*-A**$^{\bullet}$ in the PCM(ACN) on the XMCQDPT-CASSCF level of theory using the aug-cc-pVDZ basis set. As evident from the calculations (**Figure S14 a**), the absorption spectrum of ***p*-BA**$^{\bullet-}$ is in very good agreement with the experimentally observed transient spectrum. This proofs electron transfer from the excited deazaflavin radical anion to the substrate ***p*-BA** forming the ***p*-BA**$^{\bullet-}$. Furthermore, calculation of the relaxed potential energy surface along the C-Br bond for ***p*-BA** and ***p*-BA**$^{\bullet-}$ (**Figure S14 b**) shows that on the one hand the bond length is considerably enlarged from 1.92 to 2.86 Å and on the other hand the dissociation energy is significantly reduced from 4.3 to 0.09 eV, respectively. Although the C-Br bond is clearly weakened in ***p*-BA**$^{\bullet-}$, its dissociation energy is still within the thermal energy range allowing its transient observation which is in excellent agreement with our experimental data (**Figure 5 g**).

[..]

2) Lines 135-136, "Thus, a deprotonated form of the key intermediate is beneficial.": The authors should explain the reason why the base was not added in the absorption measurements (Figures 3-5). By the comparison with the results of the basic condition, it may possible to confirm what is the deprotonated key intermediate.

**Reply:** We totally agree with the reviewer. However, in the present study we first of all focused on the general understanding of the photocatalytic reaction mechanism for the dehalogenation, which turned out to be of conPET type. Additional time-resolved studies investigating the role of the protonation state are currently ongoing in our labs and will be the topic of subsequent manuscripts. We added in the main text the following line: [..] The reactivity of the excited states of compound **1** and **5** with the sacrificial substrate DIPEA was investigated by time-resolved absorption and emission spectroscopy in the absence of any additional base, since the main focus was on the understanding of the underlying photocatalytic reaction mechanism (Further studies on the role of specific deprotonation states is currently ongoing in our labs and will be presented elsewhere). [..]

Furthermore, we performed further computational calculations and provided an additional section 4.4 in the SI including one additional Figure (Figure S 13) to address the protonation state of $\mathbf{5_{sq}}$. This now reads: [..]

**4.4 Theoretical absorption spectra of all protonation states of $\mathbf{5_{sq}}$**

The total photocatalytic conversion from ***p*-BA** to ***p*-A** (anisole) shows a dependence on the used solvent, the used excitation wavelength, and the basicity. Since the semiquinone form, $\mathbf{5_{sq}}$, of the photocatalyst is one key intermediate for the successful dehalogenation, we calculated quantum chemically absorption spectra of $\mathbf{5_{sq}}$ and its two potential protonation states, *i.e.* $\mathbf{5_{sq}^{C\text{-}H}}$ and $\mathbf{5_{sq}^{N\text{-}H}}$ in PCM for ACN and DMF (**Figure S13**). As evident, the theoretical and experimental absorption spectra of $\mathbf{5_{sq}}$ in ACN are in excellent agreement (see section 4.1 and **Figures S10** and **S13 a**). Considering the significant deviations of the absorption spectra of both protonated forms (**Figure S13 b** and **c**) from the experimentally recorded transient spectrum (**Figures 4** in the main text and **S10**) in the absence of base, we can already exclude the involvement of these species under neutral conditions and, thus, identify $\mathbf{5_{sq}}$ as the key intermediate of the conPET reaction. Furthermore, this is in accord with the observation that with excitation wavelength < 400 nm the total conversion under otherwise identical reaction conditions increased by a factor of 1.6, since $\mathbf{5_{sq}}$ has its highest absorption probability at around 360 nm. Moreover, the calculation shows that the most intense transitions around 365 nm of the $\mathbf{5_{sq}}$ in DMF shift apart resulting in an expected overall decreased extinction coefficient compared to the situation in ACN (**Figure S13 a**). Therefore, this might explain the observed decreased conversion yield by a factor of *ca.* 2 in DMF (**Table S4**).

In order to get more insights on the impact of the basicity on the reaction mechanism further studies are currently ongoing in our lab. At present, the working hypothesis explaining the observed enhanced yields under basic conditions are given by the following possible scenarios: 1) enhanced triplet yield; 2) longer triplet lifetime; 3) enhanced **$dFl_{sq}$** yield; or 4) longer lifetime of **$dFl_{sq}$**. However, an enhancement of the final dehalogenation step after initiation by the second electron transfer can already be excluded since this part of the reaction is identical, thus, independent on the tested deazaflavin. [..]

Correspondingly, we modified the end of the "Photodehalogenation'' section that now reads as follows: [..] Thus, a deprotonated form of one key intermediate seems to be beneficial (A more detailed discussion can be found in section 4.4 in the SI). [..].

3) The present conPET system of the deazaflavins seems to be attractive, but the flavin-type catalysts are often unstable in the strongly basic reaction condition. Is there any information on the stability of the catalysts 1-5 in the reaction condition, e.g. in the condition of Table 2?

**Reply:** We thank the reviewer for this very important question. Unfortunately, we did not check the degradation of the photocatalysts *via* stationary absorption spectroscopy, since we did not recognize any significant colour change after the preparative experiments that could indicate strong degradation. While with $Cs_2CO_3$ no obvious colour change was observed, the colour changed slightly from yellow to orange to the end of the experiment when using the stronger base KO$^t$Bu. Thus, we can only estimate that the degradation under the used conditions is not severe on a preparative scale. In all time-resolved spectroscopic experiments we did not detect any degradation of the catalysts.

4) The abbreviated names of deazaflavins should be unified in the main-text and Supporting Information. For examples, dFl-Phox should be changed to 5ox.

**Reply:** We agree entirely with the reviewer and thank him/her for his/her support. We made the nomenclature in the SI consistent with that in the main text.

[1] W. Zhang, K. L. Carpenter, S. Lin, *Angew Chem Int Ed Engl* **2020**, *59*, 409-417.

REVIEWERS' COMMENTS:

Reviewer #1 (Remarks to the Author):

The authors have satisfactorily addressed my remarks and I recommend publication.

Reviewer #2 (Remarks to the Author):

I am satisfied that the authors have carefully addressed the comments raised in my review and by the other reviewers. I am therefore happy to support publication of this very interesting article.

Reviewer #3 (Remarks to the Author):

The manuscript entitled "Deazaflavin reductive photocatalysis involves excited semiquinone radicals" by Graml et al. have given the satisfactory response to the queries raised by the reviewers. In my opinion, this interesting manuscript is suitable for publication in Nature Communications.
